# Modelling environmentally suitable areas for the potential introduction and cultivation of the emerging oil crop *Paeonia ostii* in China

**DOI:** 10.1038/s41598-019-39449-y

**Published:** 2019-03-01

**Authors:** Li-Ping Peng, Fang-Yun Cheng, Xian-Ge Hu, Jian-Feng Mao, Xing-Xing Xu, Yuan Zhong, San-Yuan Li, Hong-Li Xian

**Affiliations:** 1 Beijing Key Laboratory of Ornamental Plants Germplasm Innovation & Molecular Breeding, National Engineering Research Center for Floriculture, Beijing Laboratory of Urban and Rural Ecological Environment, School of Landscape Architecture, Beijing Forestry University, Beijing, 100083 China; 2 Key Laboratory of Genetics and Breeding in Forest Trees and Ornamental Plants, Ministry of Education, College of Biological Sciences and Technology, Beijing Forestry University, Beijing, 100083 China; 3 Forestry Department of Shaanxi Province, Xi’an, Shaanxi 710082 China

## Abstract

*Paeonia ostii* is a traditional ornamental and medicinal species that has attracted considerable interest for its high oil value. To facilitate the effective and rational cultivation and application of *P*. *ostii* in China, it is necessary to determine its potential spatial habitat distribution and environmental requirements. Using high-resolution environmental data for current and future climate scenarios, the potential suitable area and climatic requirements of *P*. *ostii* were modelled. Among the 11 environmental variables investigated, growing degree days, precipitation of the wettest month, mean temperature of the coldest quarter, global UV-B radiation, annual precipitation, and soil pH played major roles in determining the suitability of a habitat for the cultivation of *P*. *ostii*. Under the current environmental conditions in China, a total area of 20.31 × 10^5^ km^2^ is suitable for growing *P*. *ostii*, accounting for 21.16% of the country’s total land area. Under the two future climate scenario/year combinations (i.e., representative concentration pathways [RCPs], RCP2.6 and RCP8.5 in 2050), this species would increase its suitable area at high latitudes while decrease at low latitudes. These results present valuable information and a theoretical reference point for identifying the suitable cultivation areas of *P*. *ostii*.

## Introduction

*Paeonia ostii*, a species of tree peony (Sect. *Moutan*, Paeonia, Paeoniaceae; 2n = 10)^1,2^, has been traditionally cultivated for medicinal purposes throughout Asia for more than 1,600 years because of its antispasmodic value^3,4^. Recently, the seeds of this species have been shown to be rich in unsaturated fatty acids, especially *α*-linolenic acid^5–8^, which indicates that oil from its seeds can be used as a novel source of high-quality edible oils. Due to its great potential for producing edible oils, *P*. *ostii* has been recognized in the national project to relieve the perpetual oil crisis in China^9^, and the cultivated area of this species has rapidly increased to over 33.3 × 10^4^ ha since 2013^10^.

*P*. *ostii* native to China is a deciduous, multi-stemmed, woody shrub with a preference for sun and suitability for both dry-cold and wet-warm climate conditions. It is widely cultivated in Bozhou and Tongling Cities in Anhui province, and sporadically in Hubei, Shaanxi, and Sichuan provinces in China as a traditional medicine^3^. In recent years, *P*. *ostii* has been cultivated for multiple purposes in many new regions, including Hebei, Yunnan, Sichuan, Xinjiang, Inner Mongolia, and Gansu Provinces, among others. The growth and development of *P*. *ostii* are affected by various environmental variables such as temperature, moisture, light, soil conditions, and landscape properties^8,11–14^. Previous research showed that the oil content, *α*-linolenic acid content and seed yield vary with geographical environment and changes in climatic conditions: the *α*-linolenic acid content in Xunyang (Shaanxi Province) is significantly higher than that in Tongling (Anhui Province)^8^, and the seed yield per plant in BoZhou (Anhui Province) is about four times that of Shaoyang (Hunan Province)^15^. Temperatures were reported to affect the vegetative growth^16^, flower bud differentiation^17^, photosynthetic characteristics^13^ and seed dormancy and germination^18^ of *P*. *ostii*. Although *P*. *ostii* is comparably resistant to cool temperatures, heat, wet conditions, disease, and exhibits wide adaptability^3,19^, it is still unclear whether all these new cultivation regions are suitable for the growth of this species. The selection of suitable cultivation areas has important implications for sustainable use, so as to avoid planting blindly and ensure good growth. Therefore, mapping the climatically suitable areas for *P*. *ostii* and identifying key environmental factors are urgently needed for the introduction and cultivation of this species in China.

Species distribution modeling (SDM) is widely used in ecology, biogeography, and evolutionary studies^20^. Whilst there are other methods available for modelling species distributions, (such as Grap, Bioclim, and Domain), MaxEnt is one of the most widely applied approaches in the field of crop science and has been used by agricultural industries for modelling crop plant distributions under both current and future environmental conditions^21,22^. MaxEnt uses presence-only data and the associated environmental variables to project the potential habitat distribution of a target species based on the principles of the maximum entropy algorithm^22,23^. This process has been widely applied to estimate the ecological requirements of medicinal plants (e.g., H. *riparia*, Lour)^24^, major forest tree species (e.g., *Platycladus orientalis* and *Davidia involucrata*)^25,26^, invasive plants (e.g. *Mimosa diplotricha* and *Mikania micrantha*)^27^ and endangered relic species (*Schisandra sphenanthera* and *Ammopiptanthus mongolicus*)^28,29^, and then to characterize and map their suitable growing areas at a landscape scale.

To evaluate the environmental variables and properties of habitat distribution that shape habitat suitability, MaxEnt was used to model and predict distributions of *P*. *ostii* based on an extensive collection of geo-referenced occurrence records and associated current and future environmental data. This study aims to: (1) identify the main environmental variables that constrain the distribution of *P*. *ostii*, (2) map *P*. *ostii*’*s* climatically suitable areas and estimate its climatic thresholds, (3) determine different areas degrees of suitability for cultivating *P*. *ostii* nationally, and, (4) predict the spatial patterns of changes in the suitable cultivation areas of *P*. *ostii* under two future climate change scenario/year combinations: RCP2.6-2050 (average for the years 2041–2060 under the RCP2.6 scenario) and RCP8.5-2050 (average for the years 2041–2060 under the RCP8.5 scenario). This work provides valuable information on the distribution of *P*. *ostii* and reference data to assist land managers in the development of effective plans for large-scale increases in cultivation across China.

## Methods

### Occurrence and distribution of *P*. *ostii*

**Figure 1:**
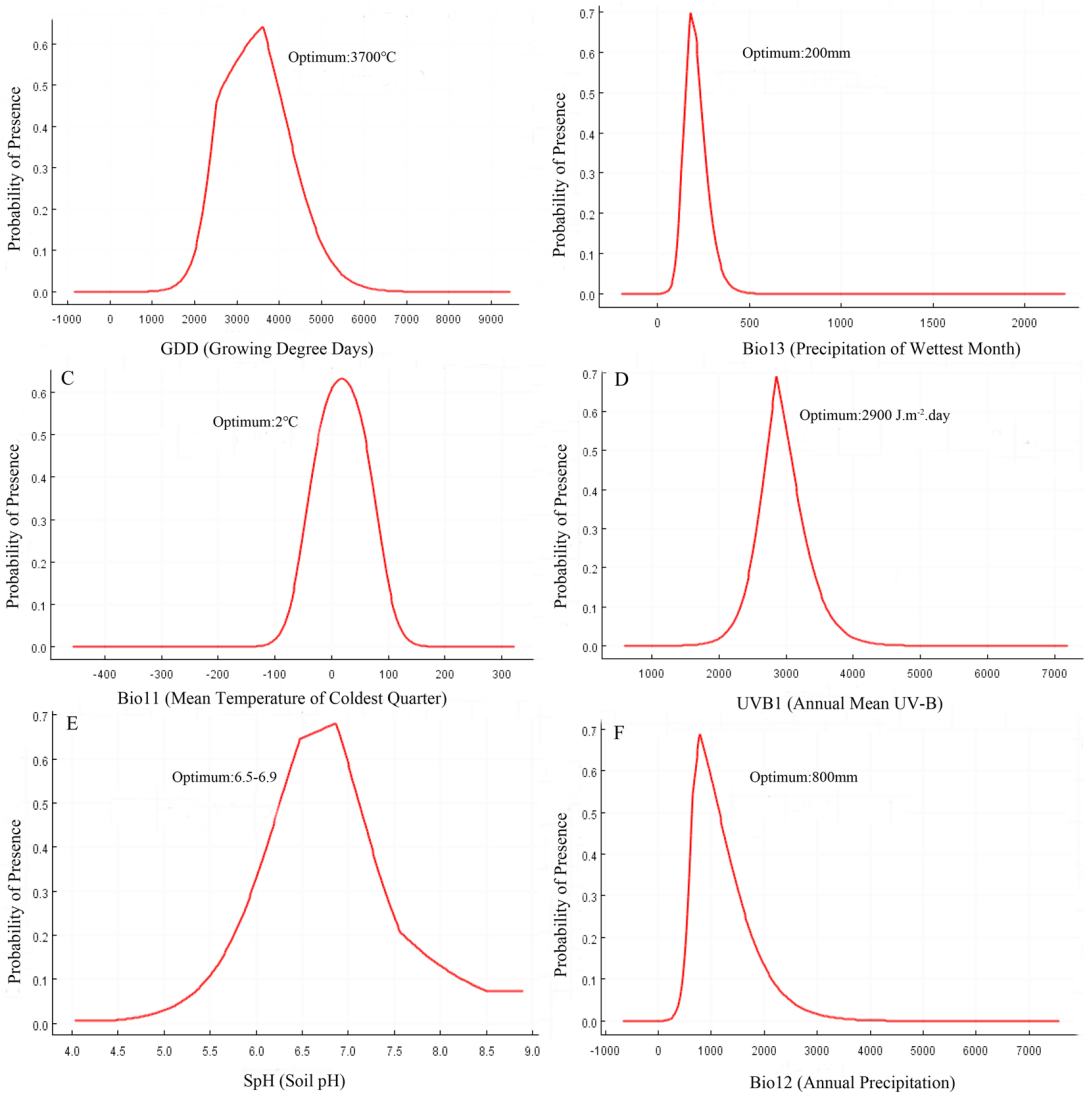
The distribution of occurrence records and the current spatial distribution map of *P*. *ostii* in China. Blue triangle indicates the occurrence records of *P*. *ostii*. Modelling of the current distribution of *P*.*ostii* was performed by MaxEnt v3.3.3 (http://www.cs.princeton.edu/~schapire/maxent/), and the whole map was generated using the tool of ArcMap 10.0 (ESRI, Redlands, CA, USA, http://www.esri.com/).

The occurrence records of *P*. *ostii* were obtained from three resources: (1) published literature^30–32^, (2) the Chinese Virtual Herbarium (CVH, http://v5.cvh.org.cn/ ^33^, a free and open access database that holds plant distribution records from all main herbaria across the country) and (3) our systematic fieldwork carried out in China in 2014. When occurrence records lacked exact geo-coordinates, Google Earth was used (http://ditu.google.cn/) to determine the latitude and longitude. In total, we collected a total of 87 geo-referenced occurrence records of the species within its known native distribution (Table S1 and Fig. 1).

### Current environmental data

In the current study, 29 environmental variables (Table S2) that may influence the distribution of *P*. *ostii* were initially selected to model the current species distribution patterns. These included 19 climatic variables (Bio1-Bio19) at spatial resolutions of 1 km obtained from the WorldClim website, which uses observed monthly temperature and precipitation data sourced from globally distributed weather stations, averaged over the period 1950–2000 (www.worldclim.org)^34^. The other ten environmental variables were obtained as follows: soil organic carbon (SC), soil pH (SpH), and growing degree days (GDD) from the Center for Sustainability and the Global Environment (http://www.sage.wisc.edu/atlas/index.php)^35^, ground-frost frequency (FRS), wet-day frequency (WET) and vapor pressure (VAP) from the IPCC database (http://www.ipccdata.org/obs/cru_ts2_1.html), and global UV-B radiation (UVB1, UVB2, UVB3, UVB4) from the gIUV database (http://www.ufz.de/gluv/)^36^. These data were preprocessed to a general spatial resolution of 30 s latitude/longitude (ca. 1 km^2^ at ground level).

Strong collinearity between the environmental variables could artifically inflate the accuracy of the model^37^. To avoid the potential problem of multi-collinearity in the meteorological and environmental factors, all the variables were subjected to a principal component analysis (PCA) to reduce the dimensionality of the models and avoid multi-collinearity (see the summary of variable correlations in Table S3). After evaluation, we retained 11 environmental variables (Bio3, Bio6, Bio11, Bio12, Bio13, Alt_China, GDD, UVB1, SpH, SC, WET) with pairwise Pearson correlation coefficients of r ≤ 0.70, for subsequent analyses.

### Future climate scenarios and data

To predict potential changes of the geographic distribution of *P*. *ostii*, we used the BCC-CSM1.1 GCM, one of the most-used models currently available for simulating the global climate’s response to increasing greenhouse gas concentrations^38^, and simulated two of the four RCPs (i.e., RCP2.6, RCP4.5, RCP6.0 and RCP8.5) that were used in the 5^th^ IPCC Assessment Report^39,40^. RCP 2.6 represents the most “benign” scenario (i.e., a likely average global temperature increase of 0.3–1.7 °C for ca. 2081–2100), whereas RCP 8.5 is the most extreme scenario (a likely global temperature increase of 2.6–4.8 °C for ca. 2081–2100). For modeling purposes, climate variables were selected for simulation in two climate change scenario/year combinations: RCP2.6-2050 (average for the years 2041–2060 under the RCP2.6 scenario) and RCP8.5-2050 (average for the years 2061–2080 under the RCP8.5 scenario). Climate variables for future climate scenarios were also downloaded from the WorldClim website. The ten environmental parameters (SC, SpH, GDD, FRS, WET, VAP and four UV-B radiation parameters) remained unchanged for the following analyses of SDM projections under future climate conditions.

### Evaluation of current and future potential habitat

MaxEnt v3.3.3^22,23^ was used as the algorithm to model the current and future habitat distribution of *P*. *ostii* in China. Model prediction parameters for the baseline model (representing current environmental conditions) were: convergence threshold = 10^−5^, maximum iterations = 1000, regularization value *β* = 10^−4^, and use of linear, quadratic, product and binary features^23^.75% of the location point data were used for training the model, while the remaining 25% were used to validate the MaxEnt model. The algorithm was set to run either 1000 iterations of these processes or to continue until convergence (threshold 0.00001). The logistic output of each MaxEnt run was then interpreted as a probability map. In addition to visual inspection of the output maps, model performance was evaluated using the area under the receiver operating characteristic curve (AUC), where an AUC = 0.5 indicated that the model predicted outcomes no better than random chance, an AUC ≥ 0.7 indicated strong predictive power, and an AUC = 1.0 indicated a perfectly fitted model^41^. The Jackknife test was used to assess the relative importance of the variables. The distribution of *P*. *ostii* in terms of each environmental variable was displayed in kernel density plots.

### Area suitability mapping and output divisions

The outputs were transformed into raster format using the ArcMap tool in ArcGIS v10.0 software^42^ for further analysis. To define habitat versus non-habitat for *P*. *ostii*, we used the “maximum training sensitivity plus specificity” threshold that produced highly accurate predictions^43^, and classified the appropriate level of division by using the “reclassify” function of the spatial analysis tools in ArcGIS. According to the weighted average method, spatial analysis in ArcGIS was applied to calculate the rasters, producing maps that can be used to assess habitat suitability at resolutions of a 1 km × 1 km grid cell^28^. The calculated raster data were then exported into a spreadsheet.

## Results

### Model performance and important environmental variables

Model performance for *P*. *ostii* was classified as being better than random, with a training AUC of 0.983 and test AUC of 0.979 (Fig. S1). The test AUC was close to 1, which indicated that the model performed better than random and therefore showed the high accuracy of the model.

**Figure 2:**
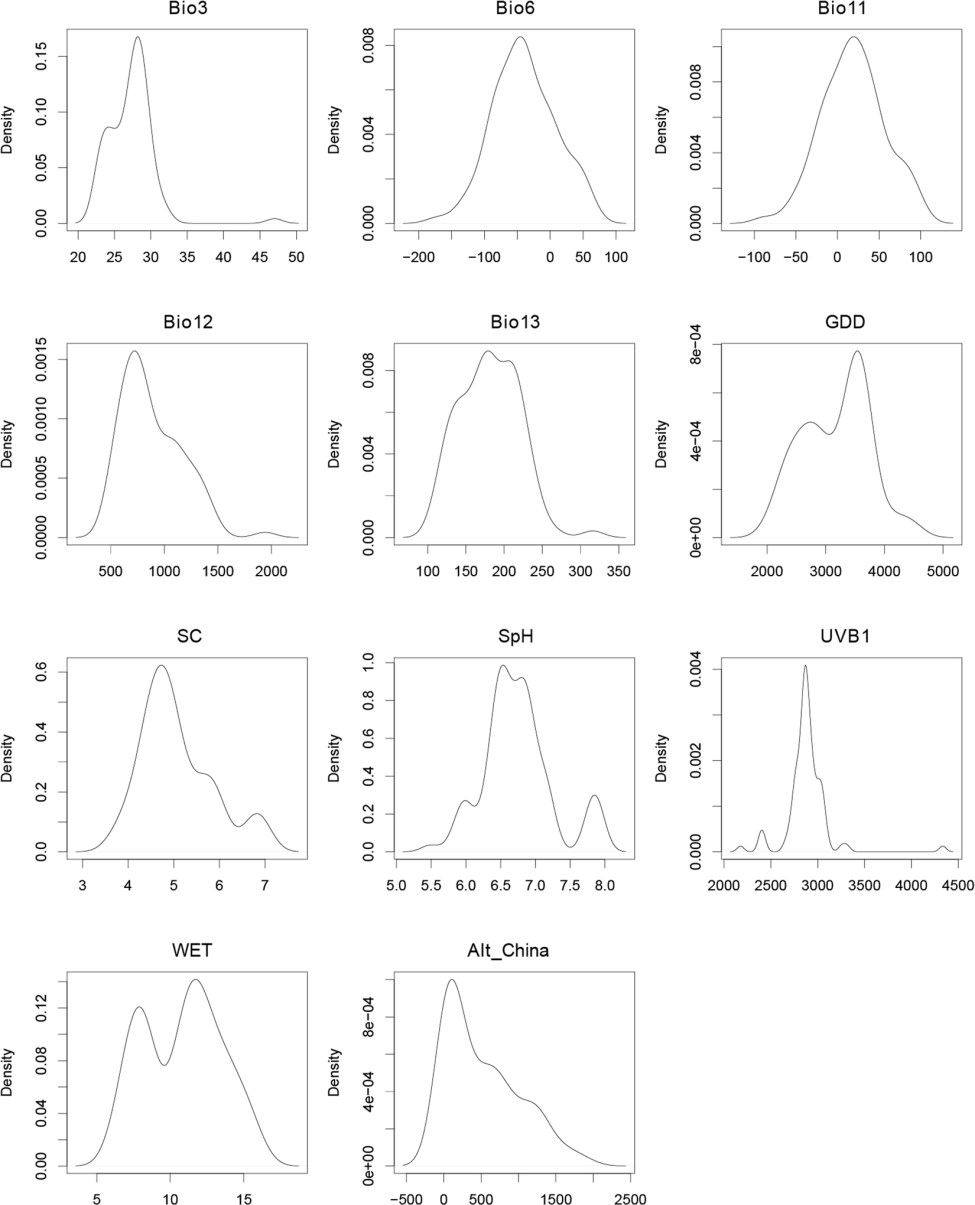
Response curves for important environmental variables in the species distribution model for *P*. *ostii*.

The estimates of relative contributions of the environmental variables to the MaxEnt model and the ranges of the 11 environmental variables across the four habitat classes is shown in Table 1. Among the 11 environmental variables, growing degree days (GDD, 26.4%), precipitation of the wettest month (Bio13, 17.7%), mean temperature of the coldest quarter (Bio11, 16.9), annual mean UV-B radiation (UV-B, 12.2%), annual precipitation (Bio12, 9.7%), and soil pH (SpH, 6.1%) played major roles in determining the suitability of a habitat for the cultivation of *P*. *ostii*, with cumulative contributions as high as 89% (Table 1). The response curves of these six predictor variables showed unimodal relationships, with the response peak representing the optimum environmental conditions for *P*. *ostii*: GDD at 3700 °C, Bio13 at 200 mm, Bio11 at 2 °C, UVB1 at 2900 J.m^−2^.day, Bio12 at 800 mm and SpH at 6.5–6.9 (Fig. 1). The frequency distribution of *P*. *ostii* for each environmental variable is presented in the kernel density plots in Fig. 2.

**Table 1:** Percentage of variable contribution to the model construction derived from the permutation importance analysis and the ranges of the 11 environmental variables across the four habitat classes.

Variable	Unit	Relative importance %	Climatic suitable habitat map			
			Highly suitable area (0.6–1.0)	Moderately suitable area (0.4–0.6)	Marginally suitable area (0.166–0.4)	Unsuitable area (0.0–0.166)
Growing Degree Days (GDD)	°C	26.4	3500–3779	2812–3412, 3779–4385	2003–2753, 4385–4850	<2003, >4850
Precipitation of the Wettest Month (Bio13)	mm	17.7	180–242	150–180, 242–264	111–147, 264–316	<111, >316
Mean Temperature of the Coldest Quarter (Bio11)	°C	16.9	0–4	−2.6–0.4, 4.1–7.9	7.9–9.8, −3–8.8	<−8.8, >9.8
Annual Mean UV-B (UVB 1)	J m^−2^ · day^−1^	12.2	2806–3107	2709–2781	2177–2691, 2781–4334	<2177, >4334
Annual Precipitation (Bio12)	mm	9.7	709–1094	605–698, 1104–1466	200–598, 1466–1942	<200, >1942
Soil pH (SpH)	—	6.1	6.42–7.1	6.0–6.38, 7.1–7.22	5.5–6.0, 7.7–8.0	<5.5, >8.0
Altitude (Alt_China)	m	3.8	1–155	160–1275	1354–2500	>2500
Min Temperature of the Coldest Month (Bio6)	°C	2.9	−8.6–2.4	−11.9–9, −1.9–2.2	−17.1–12.2, 3.9–6.2	<−17.1, >6.2
Soil Organic Carbon (SC)	—	1.8	4.18–4.80	4.0–4.18, 4.09–6.05	3.7–3.9, 6.79–8.0	<3.7, >8.0
Wet-day Frequency (WET)	%	1.7	10.2–10.99	7–10.2, 11.01–15.81	15.81–45	<7, >45
Isothermality (BIO2/BIO7) (Bio3)	—	0.8	26–30	31–32, 24–26	22–24, 32–36	<22, >36

### The current and potential distribution of *P*. *ostii*

**Figure 3:**
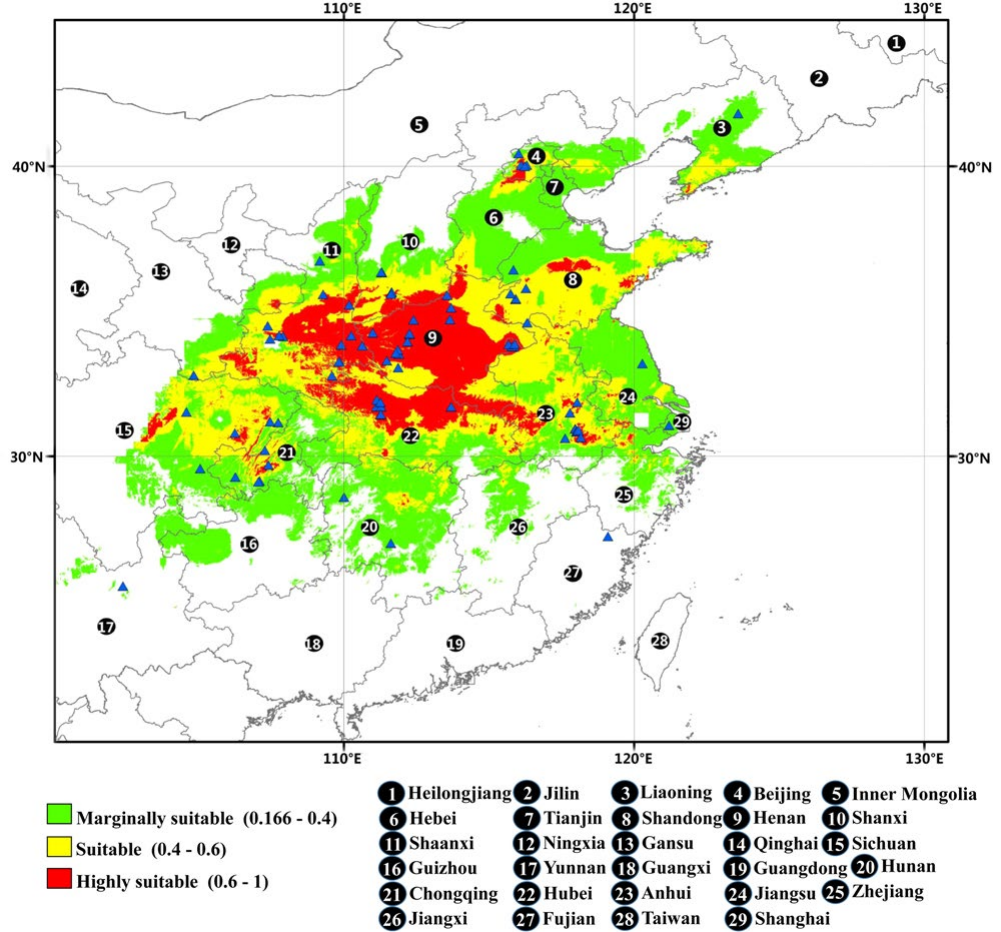
Kernel density plots for 11 environmental factors that affect the distribution of *P*. *ostii*.

Based on known occurrences of *P*. *ostii* and current climate data, a habitat map of suitable climates was produced using the MaxEnt model with 11 environmental variables. The suitable habitat areas were divided into four classes with the “maximum training sensitivity plus specificity” threshold value of 0.166, in which they were defined as: (i) highly suitable areas (0.6–1.0), (ii) moderately suitable areas (0.4–0.6), (iii) marginally suitable areas (0.166–0.4), and (iv) unsuitable areas (0–0.166) (Fig. 3). According to the map, the potential distribution area of *P*. *ostii* occurs mainly in 28 provinces in China (regions with a probability threshold >0.166): Shaanxi, Hubei, Henan, Sichuan, Hebei, Shandong, Shanxi, Anhui, Hunan, Liaoning, Jiangsu, Gansu, Chongqing, Guizhou, Zhejiang, Jiangxi, Inner Mongolia, Yunnan, Beijing, Ningxia, Tianjin, Shanghai, Jilin, Guangxi, Guangdong, Xizang, Fujian, and Heilongjiang Provinces (Tables 2 and S4). Among the provinces, Henan, Shandong, and Hebei Provinces had the most highly, moderate, and marginally suitable habitat areas for *P*. *ostii*, respectively. The current total suitable area in China for this species was predicted to encompass ca. 20.31 × 10^5^ km^2^.

**Table 2:** Portions of different classes of potential distribution area of *P*. *ostii* under current climatic and geological conditions.

PR	Unsuitable area			Marginally suitable area			Moderately suitable area			Highly suitable area			Total		
	CA (km^2^)	NC	AA (%)	CA (km^2^)	NC	AA (%)	CA (km^2^)	NC	AA (%)	CA (km^2^)	NC	AA (%)	CA (km^2^)	NC	AA (%)
Shaanxi	8,587.35	6	4.17	67,553.04	48	32.82	58,298.99	68	28.33	71,360.62	69	34.67	197,212.65	76	95.83
Hubei	10,346.38	25	5.57	57,458.51	64	30.92	59,942.39	73	32.26	58,052.72	47	31.24	175,453.62	79	94.43
Henan	0.00	0	0.00	3,586.17	17	2.15	50,247.90	80	30.09	113,165.93	110	67.76	167,000.00	122	100.00
Sichuan	321,339.35	102	66.12	82,917.57	111	17.06	73,040.67	83	15.03	8,702.41	42	1.79	164,660.65	117	33.88
Hebei	30,987.26	30	16.41	119,896.75	113	63.50	29,671.39	68	15.72	8,244.60	27	4.37	157,812.74	145	83.59
Shandong	4,006.87	18	2.54	57,394.08	79	36.34	84,496.03	79	53.50	12,044.40	48	7.63	153,934.51	111	97.46
Shanxi	22,286.35	26	14.22	56,676.29	61	36.15	43,135.24	70	27.51	34,673.12	48	22.12	134,484.65	106	85.78
Anhui	8,714.69	13	6.24	42,070.70	64	30.14	71,504.89	74	51.22	17,309.72	54	12.40	130,885.31	80	93.76
Hunan	87,808.24	77	41.46	107,290.11	95	50.66	16,222.92	39	7.66	478.73	12	0.23	123,991.76	95	58.54
Liaoning	32,309.28	34	21.83	97,110.71	55	65.62	15,846.11	15	10.71	2,733.90	6	1.85	115,690.72	55	78.17
Jiangsu	828.34	7	0.77	88,070.84	69	82.16	16,966.47	40	15.83	1,334.36	11	1.24	106,371.66	100	99.23
Gansu	359,967.71	52	79.34	53,296.51	33	11.75	33,861.10	28	7.46	6,574.68	9	1.45	93,732.29	39	20.66
Chongqing	11,965.73	18	14.52	43,810.75	38	53.17	23,858.08	33	28.95	2,768.39	17	3.36	70,437.22	38	85.48
Guizhou	118,823.74	80	67.45	56,926.14	48	32.31	417.11	6	0.24	0.00	0	0.00	57,343.26	48	32.55
Zhejiang	58,195.62	56	55.88	38,963.06	59	37.41	5,679.51	24	5.45	1,302.81	10	1.25	45,945.38	59	44.12
Jiangxi	131,642.12	86	78.87	32,821.76	66	19.67	2,415.32	14	1.45	20.80	3	0.01	35,257.88	66	21.13
Inner Mongolia	1,155,151.33	85	97.65	27,848.67	20	2.35	0.00	0	0.00	0.00	0	0.00	27,848.67	20	2.35
Yunnan	376,290.32	126	95.51	17,652.64	32	4.48	57.04	2	0.01	0.00	0	0.00	17,709.68	32	4.49
Beijing	362.24	3	2.21	15,404.69	9	93.87	644.27	4	3.93	0.00	0	0.00	16,048.96	9	97.79
Ningxia	52,324.03	17	78.80	14,010.35	7	21.10	65.62	1	0.10	0.00	0	0.00	14,075.97	7	21.20
Tianjin	0.00	0	0.00	11,088.75	6	92.63	871.60	1	7.28	10.65	1	0.09	11,971.00	6	100.00
Shanghai	0.00	0	0.00	6,307.00	9	100.00	0.00	0	0.00	0.00	0	0.00	6,307.00	9	100.00
Jilin	183,868.11	45	98.12	3,531.89	12	1.88	0.00	0	0.00	0.00	0	0.00	3,531.89	12	1.88
Guangxi	235,005.27	88	99.28	1,694.73	5	0.72	0.00	0	0.00	0.00	0	0.00	1,694.73	5	0.72
Guangdong	178,975.27	91	99.60	724.73	6	0.40	0.00	0	0.00	0.00	0	0.00	724.73	6	0.40
Xizang	1,227,801.65	77	99.95	560.29	3	0.05	0.00	0	0.00	0.00	0	0.00	560.29	3	0.05
Fujian	123,850.99	69	99.90	118.02	6	0.10	0.00	0	0.00	0.00	0	0.00	118.02	6	0.10
Heilongjiang	453,952.51	78	100.00	1.16	1	0.00	0.00	0	0.00	0.00	0	0.00	1.16	1	0.00
Total	5,195,390.72	1,310	54.12	1,104,785.92	1,137	11.51	587,242.67	802.00	6.12	338,777.84	514	3.53	2,030,806.43	1,452	21.16

Notes: PR = province (region); NC = number of cities/counties; CA = coverage area; AA = accounted area for area of cities/countries.

### Future changes in suitable areas

**Figure 4:**
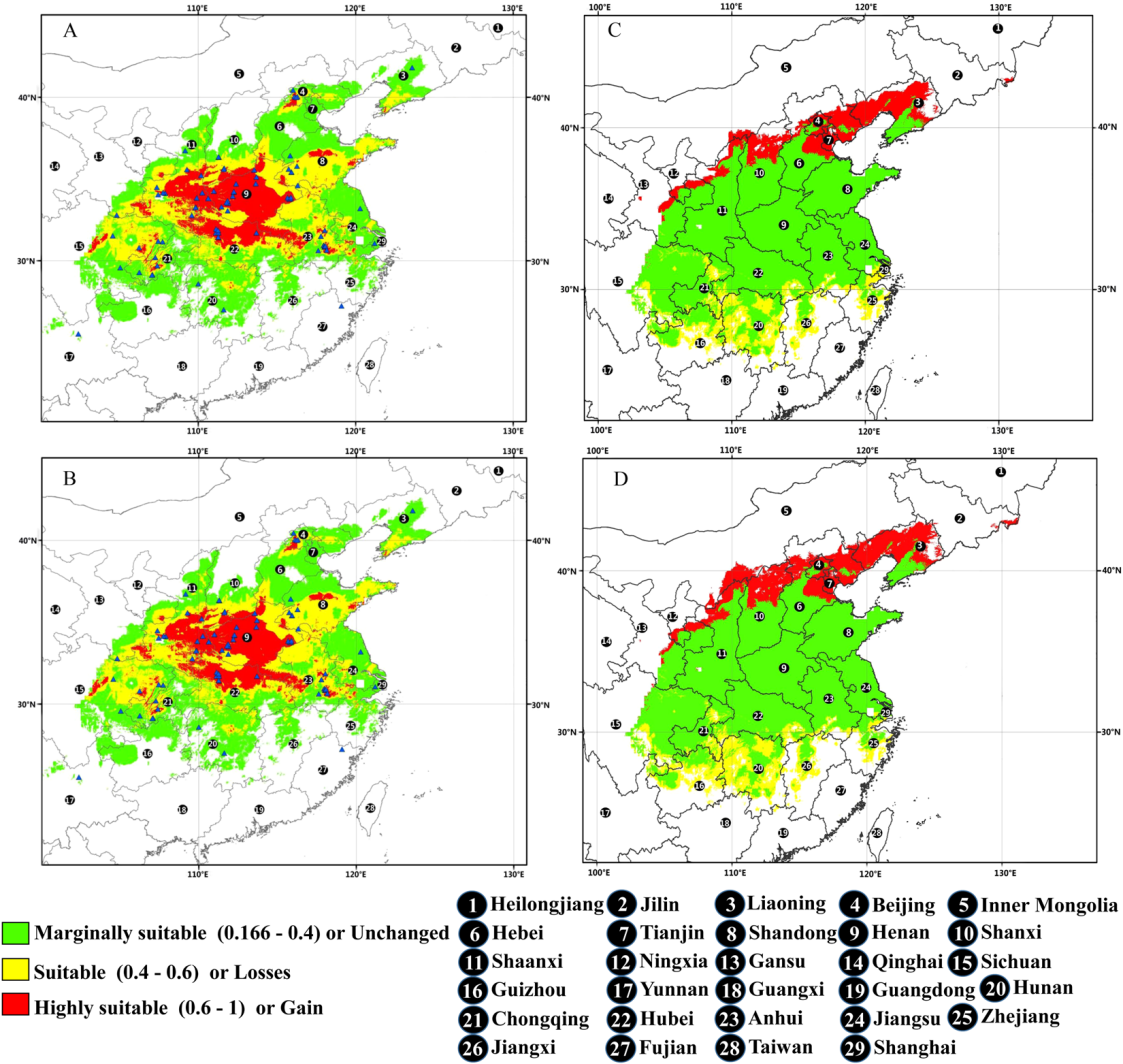
Future species distribution models (SDMs) and their spatial changes for *P*. *ostii* under the future climate scenario/year combinations RCP2.6-2050 and RCP8.5-2050. (**A**) SDM for *P*. *ostii* under future climate scenario/year combination RCP2.6-2050. (**B**) SDM for *P*. *ostii* under future climate scenario/year combination RCP8.5-2050. (**C**) Comparison between the current SDM and the SDM under future climate scenario/year combination RCP2.6-2050. (**D**) Comparison between the current SDM and the SDM under future climate scenario/year combination RCP8.5-2050. The figure is based on the prediction of the maximum entropy model using MaxEnt software for species habitat modeling (version 3.3.3 k), and the map was generated by ArcMap 10.0 (ESRI, Redlands, CA, USA, http://www.esri.com/).

Under the future climate scenario/year combination of RCP2.6-2050, MaxEnt predicted *P*. *ostii* gains in suitable habitat area in the southern sector of Inner Mongolia and Liaoning, as well as in the northern Hebei Province in the coming decades (Fig. 4B,D), amounting to ca. 4.2 × 10^5^ km^2^ (16.34% of the currently suitable areas). Losses or decreases in suitable areas would occur at low latitudes, mainly in Hunan, Sichuan, Jiangxi, and Zhejiang Provinces (Fig. 4B,D), and would amount to approximately 3.6 × 10^5^ km^2^ (14.01%). Under the RCP8.5-2050 climate scenario, the increased and decreased habitat stayed the same, with gains and losses in suitable areas of 5.3 × 10^5^ km^2^ (20.62%) and 3.9 × 10^5^ km^2^ (15.15%), respectively. Approximately 2.21 × 10^6^ km^2^ (85.99% of the current suitable areas) under RCP2.6-2050 and 2.18 × 10^6^ km^2^ (84.82%) under RCP8.5-2050 would remain unchanged (Table 3).

**Table 3:** Changes in suitable areas for *P*. *ostii* under two future climate scenario/year combinations RCP2.6-2050 and RCP8.5-2050.

Future climate scenario/year combination	Area (×10^6^ km^2^)					Proportion of area (%)			
	Future	Loss	Gain	Unchanged	Total	Loss	Gain	Unchanged	Total
RCP2.6-2050	2.27	0.36	0.42	2.21	0.06	14.01	16.34	85.99	2.33
RCP8.5-2050	2.32	0.39	0.53	2.18	0.14	15.18	20.62	84.82	5.45

## Discussion

This study performed a detailed analysis on the suitable habitat of *P*. *ostii* under current and future climate scenarios, which can serve as an important step in developing strategies and policies for the effective management and utilization of this emerging oil crop.

*P*. *ostii* has a wide distribution range in China, ranging across Bozhou and Tongling city in Anhui Province, the southwest region of Hunan Province, and sparsely across in Shanxi, Shandong Henan, Hubei, Shaanxi provinces, and Sichuan^3^. Based on large-scale environmental data, our modeling study projected a spatial distribution in China of ca. 20.31 × 10^5^ km^2^ for the species under current climate conditions, in which the center areas are in Henan, Anhui, the southern sector of Shaanxi and Shanxi, the northwest of Hubei, and the northeast of Sichuan Province. This model was in agreement with the precious occurrence records^30–32^ that found this species’ range was from 26 to 42°N and 100 to 124°E.

Our simulation results showed that temperature, precipitation, and light were among the most important factors affecting the distribution of *P*. *ostii*. Previous studies on the growth characteristics of other plant species showed that GDD as a representation of accumulated temperature were closely related to plant growth and development, including emergence, flowering, and maturity dates^44–49^. Likewise, we found that GDD were the most important factor (26.4%) for *P*. *ostii* and that its base value was 2000–5000 °C (≥10 °C) for normal growth. Temperature was reported to affect the seed dormancy and germination of *P*. *ostii*^18^. Dormancy in peony seeds typically is complex, requiring cold stratification (0–10 °C) to break the dormancy of the epicotyl^50^, while temperatures >20 °C were found to be unfavorable for seed germination. This suggests that the emergence and the death of *P*. *ostii* seedlings be directly affected by temperature, which played a main role in shaping the ecological adaptation of this species.

Water availability is correlated with many environmental factors that influence the physiological and biochemical processes of plants^51^. The number of female flowers and the root growth of *P*. *ostii* are significantly associated with precipitation amount. Abnormally-grown or even rotted roots can occure in Areas with high precipitation; while those areas with less precipitation, such as Xinjiang Province (the annual precipitation was no more than 100 mm, typically below 50 mm)^52^, will be not beneficial to the growth and development. In our modeling exercise, precipitation including Bio12 (9.7%) and Bio13 (17.7%) greatly impacted the habitat suitability of *P*. *ostii*, with base values of 40–350 mm and 500–2000 mm, respectively. This suggests that not only severe drought but heavy and concentrated precipitation, especially during flowering, would greatly impede the growth and development of *P*. *ostii*. These environmental factors obviously impacting the distribution of *P*. *ostii* would be helpful in formulating a suitable region-wide recommendation for the introduction and cultivation of it.

Notably, the intensity of UV-B radiation was identified as one of the most biologically relevant ultraviolet radiation, and it significantly affected the growth and distribution of *P*. *ostii*. In agricultural systems, UV-B radiation affects the yield of many crops^53–57^ and increased UV-B radiation can alter the photosynthetic capacity and reduce biomass production in plants^58,59^. However, our knowledge of the effects of UV-B radiation on *P*. *ostii* is very limited, and more studies on this issue are necessary. The SpH (6.1), Alt_china (2.7), SC (1.4) and WET (1.4) variables played relatively smaller roles in the growth of *P*. *ostii*. Of these factors, only SpH seemed to significantly affect the habitat suitability of *P*. *ostii*. The overall pH range of suitable areas was from 5.5 (slightly acid) to 8.5 (alkaline); however, the pH range considered highly ecologically suitable was comparatively narrow (6.5–6.9), which demonstrated that both extremely acid and saline-alkali soils could considerably diminish the suitability of *P*. *ostii* in some places.

Climate change is driving latitudinal and altitudinal shifts in species distribution worldwide^60^, although most species are generally migrating to higher latitudes and elevations as climates warm^26,61^. Consistently, we found that under both the low (RCP2.6) and the higher (RCP8.5) scenarios of concentrations of greenhouse gas emissions, the suitable habitat range of *P*. *ostii* will increase as global warming intensity proceeds. Low-latitude regions, such as Hunan, Sichuan, Jiangxi, and Zhejiang Provinces would see a decrease in distribution. Therefore, when new areas were chosen to cultivate this species, areas should be reserved for marginal populations in low-latitudes, so as to increase survival of *P*. *ostii* plants to cope with climate change in the future.

Assessment of habitat suitability is important in understanding what defines suitable ecological conditions for cultivating *P*. *ostii*, and can provide valuable information and a theoretical reference for the identification of future suitable areas. In this study, our results show that the suitable environmental areas of *P*. *ostii* accurately predicted at the county scale can be applied to the evaluation of land use patterns at a large scale. For management efficiency, the distribution of highly and moderate suitable areas for cultivating *P*. *ostii* should be focused on. In addition, China’s national policy could be utilized to encourage farmers to cultivate *P*. *ostia* for oilseed production in more regions. In agricultural production, such environment variables as temperature, precipitation, and soil features can be artificially regulated (e.g., irrigation, and soil improvement using greenhouses and shade)^62,63^. According to the key variables identified in this study, Xinjiang Province was unsuitable for *P*. *ostii* cultivation due to water-deficiencies. Howere, when irrigation facilities are used, some of the unsuitable areas in this region would become available for growing *P*. *ostii*, which would increase the land’s value for potential oilseed production.

Due to a long-term medicinal cultivation history, the modern oil use breeding of *P*. *ostii* is rather short. The modelling of suitable environmental cultivation areas in this study was based on the occurrence of this species where it was traditionally cultivated for medicinal use. Although the areas are suitable for the growth of *P*. *ostii*, it is still uncertain if these regions will produce high seed yield and quality. Therefore, additional research should focus on determining the ecological quality and crop regionalization of *P*. *ostii*.

## Supplementary information

Supplement tables

## References

[1] Hong DY, Pan KY, Zhou ZQ (2004). Circumscription of *Paeonia suffruticosa* Andrews and identification of cultivated tree peonies. Acta Phytotaxonomica Sinica.

[2] Yu L, He LX, Li JJ (1997). Comparative studies on chromosome in varieties of *Paeonia rockii* and *Paeonia suffruticosa*. Acta Horticulture Sinica.

[3] Li, J. J., Zhang, X. F. & Zhao, X. Q. Tree Peony of China. 1–206 (Encyclopaedia of China Publishing House, 2011).

[4] Cheng FY (2007). Advances in the breeding of tree peonies and a cultivar system for the cultivar group. International Journal of Plant Breeding.

[5] Tan, Z. Z. Research on the screening oil cultivars of herbaceous peony and comparison with oil cultivars of tree peony ‘Fengdan’ Master thesis, the Chinese Academy of Forestry Science (2014).

[6] Li SS (2015). Systematic qualitative and quantitative assessment of fatty acids in the seeds of 60 tree peony (*Paeonia* section *Moutan* DC.) cultivars by GC-MS. Food chemistry.

[7] Yu, S., Du, S., Yuan, J. & Hu, Y. Fatty acid profile in the seeds and seed tissues of *Paeonia* L. species as new oil plant resources. *Scientific Reports* 6, srep26944 (2016). 10.1038/srep26944 PMC4886256 27240678

[8] Han XY, Zhang YL, Niu LX, Luo JR (2014). Fatty acid composition of ‘Fengdan’ peony seed oils from different growing regions. Food Science.

[9] The Chinese Council General Office. Opinions on accelerating the development of woody oil industry. Available at, http://www.gov.cn/zhengce/content/2015-01/13/content_9386.htm (2014).

[10] Han, J. G., Li, X. Q., Liu, Z. & Hu, Y. H. Potential applications of tree peony as an oil plant. *Cereals & Oils* (2014).

[11] Zhang ZH (2015). The effects of soil metals on the composition of oil of *Paeonia ostii* seeds. Journal of Plant Interactions.

[12] Bi, Y. W., Qin, J., Wang, K. L. & Hu, Y. H. Photosynthetic characteristis of Paeonia ostii in different seasons. *Tianjin Agricultural Sciences* (2011).

[13] Zhang, Z. H. *et al*. Photosynthetic characteristics and its micro-environmental limiting factors of two main oil peony. *Bulletin of Botanical Research* (2014).

[14] Zhou SG (2010). Effects of shading on photosynthesis, and other physiological and biochemical characteristics in tree peony. Scientia Silvae Sinicae.

[15] Li XQ, Han JG, Liu Z, Liu QH, Hu YH (2014). Economic characteristics investigation and seed oil fatty acid composition analysis of *Paeonia ostii* plants in different areas. Journal of Grain and Oil.

[16] Qian, X. Y. Physiological mechanism of breaking seed dormancy and effect of seedling quality on Paeonia ostii by different tempreture and gibberellic acid. *Nanjing Agricultural University* (2009).

[17] Jiang Z, Liu ZA, Wang LS, Shu QY (2007). Relationship between the types of flower-bud differentiation and forcing successive secondary flowering of tree peonies. Acta Horticulturae Sinica.

[18] Mei YQ, Liu ZA, Song SQ (2017). Effects of temperature on seed germination and growth inhibition of epicotyl. Seed industry of China.

[19] Luo J, Han JR, Wang YL, Yang M, Fei YJ (2011). Response of heat stress on the physiological biochemistry of *Paeonia suffruticosa*. Journal of Yangtze University.

[20] Fitzpatrick M, Gotelli N, Ellison A (2013). MaxEnt versus MaxLike: empirical comparisons with ant species distributions. Ecosphere.

[21] Pearson RG, Raxworthy CJ, Nakamura M, Peterson AT (2007). Predicting species distributions from small numbers of occurrence records: a test case using cryptic geckos in Madagascar. Journal of Biogeography.

[22] Phillips SJ, Dudík M (2008). Modeling of species distributions with Maxent: new extensions and a comprehensive evaluation. Ecography.

[23] Phillips SJ, Anderson RP, Schapire RE (2006). Maximum entropy modeling of species geographic distributions. Ecological Modelling.

[24] Yi YJ, Cheng X, Yang ZF, Zhang SH (2016). Maxent modeling for predicting the potential distribution of endangered medicinal plant (h. *riparia*, lour) in yunnan, china. Ecological Engineering.

[25] Hu, X. G., Jin, Y., Wang, X. R., Mao, J. F. & Li, Y. Predicting impacts of future climate change on the distribution of the widespread conifer *platycladus orientalis*. *Plos One* **10**(7), e0132326, 26–19 (2015). 10.1371/journal.pone.0132326 PMC4488561 26132163

[26] Tang CQ (2017). Potential effects of climate change on geographic distribution of the tertiary relict tree species *Davidia involucrata* in China. Scientific Reports.

[27] Choudhury MR, Deb P, Singha H, Chakdar B, Medhi M (2016). Predicting the probable distribution and threat of invasive *mimosa diplotricha*, suavalle and *mikania micrantha*, kunth in a protected tropical grassland. Ecological Engineering.

[28] Lu CY, Gu W, Dai AH, Wei HY (2012). Assessing habitat suitability based on geographic information system (GIS) and fuzzy: a case study of *Schisandra sphenanthera* Rehd. et Wils. in Qinling Mountains, China. Ecological Modelling.

[29] Zhu, G. P., Li, H. Q., Zhao, L., Man, L. & Liu, Q. Mapping the ecological dimensions and potential distributions of endangered relic shrubs in western Ordos biodiversity center. *Scientific Reports* **6** (2016). 10.1038/srep26268 PMC4873805 27199260

[30] Ju, Z. X. Study on the ecological adaptability and cold resistance of tree peony in northeast China. *Beijing Forestry University* (2011).

[31] Wu ZF, Li P, Xu SJ, Liu Y (2008). Anti-hyperlipidemia effects of extracts from different species of cortex moutan. Lishizhen Medicine & Materia Medica Research.

[32] Zhang MH, Jin XL, Cheng FY, Lu JH, Wu S (2016). ISSR analysis on genetic diversity of *Paeonia suffruticosa* in Hunan province. Chinese Traditional & Herbal Drugs.

[33] C. V. H. Chinese Virtual Herbarium. Available at, http://www.cvh.org.cn/ (Accessed: 10th June 2015).

[34] Hijmans RJ, Cameron SE, Parra JL, Jones PG, Jarvis A (2010). Very high resolution interpolated climate surfaces for global land areas. International Journal of Climatology.

[35] New M, Hulme M, Jones P (2000). Representing twentieth-century space-time climate variability. part ii: development of 1901–96 monthly grids of terrestrial surface climate. Journal of Climate.

[36] Beckmann M (2014). Gluv: global uv-b radiation data set for macroecological studies. Methods in Ecology & Evolution.

[37] Mao JF, Wang XR (2011). Distinct niche divergence characterizes the homoploid hybrid speciation of *Pinus densataon* the Tibetan Plateau. American Naturalist.

[38] Mcsweeney CF, Jones RG, Lee RW, Rowell DP (2014). Selecting cmip5 gcms for downscaling over multiple regions. Climate Dynamics.

[39] Collins, M. *et al*. Long-term Climate Change: Projections, Commitments and Irreversibility. In Climate change 2013: The physical science basis. Contribution of working group I to the fifth assessment report of the intergovernmental panel on climate change (eds Stocker, T. F. *et al*.). (Cambridge University Press, Cambridge, United Kingdom, 2013).

[40] Vuuren DPV, Edmonds JA, Kainuma M, Riahi K, Weyant J (2011). A special issue on the RCPs. Climatic Change.

[41] Hosmer, D. W. & Lemeshow, S. Applied logistic regression, 2nd edn. (Wiley Inter-Science, Hoboken, NJ, 2000).

[42] ESRI. ArcGIS version 10.2 (GIS by ESRI, 2013). Available at, https://www.arcgis.com (Accessed 20th September 2015).

[43] Jiménez-Valverde A, Lobo JM (2007). Threshold criteria for conversion of probability of species presence to either-or presence-absence. Acta Oecologica.

[44] Trisurat Y, Shrestha RP, Kjelgren R (2011). Plant species vulnerability to climate change in Peninsular Thailand. Applied Geography.

[45] Zhou, X. T. *et al*. Regionalization of habitat suitability of masson’s pine based on geographic information system and fuzzy matter-element model. *Scientific Reports* **6** (2016). 10.1038/srep34716 PMC5046140 27694967

[46] Waha K, Müller C, Rolinski S (2013). Separate and combined effects of temperature and precipitation change on maize yields in sub-saharan africa for mid- to late-21st century. Global & Planetary Change.

[47] Hou P (2014). Temporal and spatial variation in accumulated temperature requirements of maize. Field Crops Research.

[48] Sadras VO, Monzon JP (2006). Modelled wheat phenology captures rising temperature trends: Shortened time to flowering and maturity in Australia and Argentina. Field Crops Research.

[49] Iannucci A, Terribile MR, Martiniello P (2008). Effects of temperature and photoperiod on flowering time of forage legumes in a Mediterranean environment. Field Crops Research.

[50] Baskin, C. C. & Baskin, J. M. Seeds: Ecology, biogeography, and, evolution of dormancy and germination. 2nd edn (Academic Press, 2014).

[51] Wang JR, Hawkins CDB, Letchford T (1998). Photosynthesis, water and nitrogen use efficiencies of four paper birch (*betula papyrifera*) populations grown under different soil moisture and nutrient regimes. Forest Ecology & Management.

[52] Ding, Y. H. Climate of China. Vol. 1 (Science Press, 2013).

[53] Madronich S, McKenzie RL, Björn LO, Caldwell MM (1998). Changes in biologically active ultraviolet radiation reaching the Earth’s surface. Photochemical & Photobiological Sciences Official Journal of the European Photochemistry Association & the European Society for Photobiology.

[54] Teramura AH (1983). Effects of ultraviolet-B radiation on the growth and yield of crop plants. Physiologia Plantarum.

[55] Prado, F. E. *et al*. UV-B Radiation, Its effects and defense mechanisms in terrestrial plants//Environmental adaptations and stress tolerance of plants in the era of climate change. (Springer New York, 57–83 2012).

[56] Kumagai T, Hidema J, Kang HS, Sato T (2001). Effects of supplemental UV-B radiation on the growth and yield of two cultivars of Japanese lowland rice (*Oryza sativa* L.) under the field in a cool rice-growing region of Japan. Agriculture Ecosystems & Environment.

[57] Yin LN, Wang SW (2012). Modulated increased UV-B radiation affects crop growth and grain yield and quality of maize in the field. Photosynthetica.

[58] Xiong FS, Day TA (2001). Effect of solar ultraviolet-B radiation during springtime ozone depletion on photosynthesis and biomass production of Antarctic vascular plants. Plant Physiology.

[59] Jansen MA, Martret BL, Koornneef M (2010). Variations in constitutive and inducible UV-B tolerance; dissecting photosystem II protection in *Arabidopsis thaliana* accessions. Physiologia Plantarum.

[60] Bertrand R (2011). Changes in plant community composition lag behind climate warming in lowland forests. Nature.

[61] Zhang K, Yao L, Meng J, Tao J (2018). Maxent modeling for predicting the potential geographical distribution of two peony species under climate change. Science of the Total Environment.

[62] Zhang XB (2016). Production suitability regionalization study of *Pinus massoniana*. China Journal of Chinese Materia Medica.

[63] Chen J, Xie CX, Sun CZ, Zhao RB, Xu R (2008). Numerical analysis of suitability of C*istanche deserticola*. Journal of Chinese Materia Medica.

