# Supplementary material for: Modelling environmentally suitable areas for the potential introduction and cultivation of the emerging oil crop Paeonia ostii in China
Source: Sci Rep. 2019 Mar 1;9:3213. doi: 10.1038/s41598-019-39449-y (PMC6397192; doi:10.1038/s41598-019-39449-y)
Supplement: Supplementary file 1 — Supplement tables [file 41598_2019_39449_MOESM1_ESM.doc]

**Modelling** **environmentally suitable areas for the potential introduction and cultivation of the emerging oil crop *Paeonia ostii* in China**

**Li-Ping Peng^1^, Fang-Yun Cheng^1^** ***, Xian-Ge Hu^2^, Jian-Feng Mao^2^** ***, Xing-Xing Xu^1^,** **Yuan Zhong^1^, San-Yuan Li^3^, Hong-Li Xian****^3^**

^1^ Beijing Key Laboratory of Ornamental Plants Germplasm Innovation & Molecular Breeding, National Engineering Research Center for Floriculture, Beijing Laboratory of Urban and Rural Ecological Environment, School of Landscape Architecture, Beijing Forestry University, Beijing, 100083, China

^2^ Key Laboratory of Genetics and Breeding in Forest Trees and Ornamental Plants, Ministry of Education, College of Biological Sciences and Technology, Beijing Forestry University, Beijing, 100083, China

^3^ Forestry Department of Shaanxi Province, Xi’an, Shaanxi, 710082, China

* **Correspondence:**

Dr. Fang-Yun Cheng (E-mail: chengfy8@263.net)

Dr. Jian-Feng Mao (E-mail: jianfeng.mao@bjfu.edu.cn)

**Fig. S1. The area under the receiver operating characteristic curve of the model**


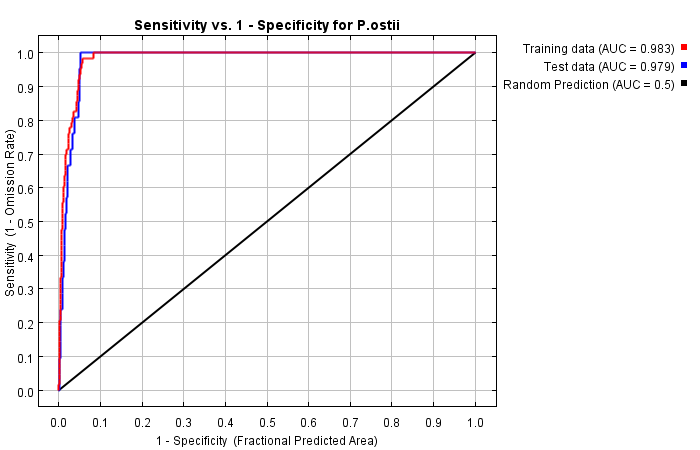


**Table S1. Collection sites of geographic distribution**

**Table S2. Environmental variables used in the study and their percentage contribution**

**Table S3. Multi-collinearity results of the environmental variables used in the study Multi-collinearity test was conducted by Arcgis 10.0 using multivariate band collection statistics to examine the cross-correlation**

**Table S4. Areas of highly suitable, moderately suitable, marginally suitable, and unsuitable habitats in every county or district in China**

| **Table S1 Collection sites of geographic distribution** | | | |
| --- | --- | --- | --- |
| **Code** | **Location** | **latitude** | **longitude** |
| 1 | Wangxing zhuang village, Huatuo town, Bozhou city, Anhui Province | 34.6114 | 116.3146 |
| 2 | Shibali town, Qiaocheng district, Bozhou city, Anhui Province | 33.8551 | 115.6664 |
| 3 | Shibali town, Qiaocheng district, Bozhou city, Anhui Province | 33.8661 | 115.6743 |
| 4 | ShiJiuli town, Qiaocheng district, Bozhou city, Anhui Province | 33.7779 | 115.8490 |
| 5 | Shijiuli town, Qiaocheng district, Bozhou city, Anhui Province | 33.7824 | 115.8499 |
| 6 | Shijiuli town, Qiaocheng district, Bozhou city, Anhui Province | 33.7958 | 115.7815 |
| 7 | Ligezi village, Wuma town, Bozhou city, Anhui Province | 33.8657 | 115.6721 |
| 8 | Ligezi village, Wuma town, Bozhou city, Anhui Province | 33.8740 | 115.8795 |
| 9 | Ligezi village, Wuma town, Bozhou city, Anhui Province | 33.8775 | 115.8826 |
| 10 | Yingpin mountain, Chao lake, Anhui Province | 31.5026 | 117.7875 |
| 11 | Jiuhua mountain drug plantation, Qingyang country, Anhui Province | 30.6348 | 117.6237 |
| 12 | Longshan village, Hewan town, Nanling county, Anhui Province | 30.8472 | 118.0157 |
| 13 | Tieshan village, Yashan town, Nanling county, Anhui Province | 30.8648 | 118.0571 |
| 14 | Mudan village, Shunan town, Tongling country, Anhui Province | 30.9607 | 117.9628 |
| 15 | Taofeng village, Shunan town, Tongling country, Anhui Province | 30.9562 | 117.9676 |
| 16 | Fenghuang mountain, Shunan town, Tongling country, Anhui Province | 30.8821 | 118.0224 |
| 17 | Fenghuang mountain, Shunan town, Tongling country, Anhui Province | 31.8750 | 118.0303 |
| 18 | Fenghuang mountain, Shunan town, Tongling country, Anhui Province | 30.8712 | 118.0218 |
| 19 | Jin feng village, Zhongming town, Tongling country, Anhui Province | 30.9142 | 118.0752 |
| 20 | Jing country, Xuancheng city, Anhui Province | 30.6553 | 118.1572 |
| 21 | Beijing Jiufeng National Forest Park | 40.0339 | 116.0990 |
| 22 | Dayushu town, Yanqing county, Beijing | 40.4365 | 116.0218 |
| 23 | Muzhuwa, Fanpo town, Song country, Henan Province | 34.2089 | 112.2523 |
| 24 | Bailonggou, Fanpo town, Song country, Henan Province | 34.2319 | 112.2595 |
| 25 | Muzhi town, Song country, Henan Province | 33.9507 | 112.1930 |
| 26 | Baotianman, Nanyang city, Henan Province | 33.5305 | 111.9029 |
| 27 | Guanshan Geopark, Hui country, Henan Province | 35.5603 | 113.5469 |
| 28 | Chenjia village, Guandu Town, Lushi country, Henan Province | 34.2581 | 110.9867 |
| 29 | National Peony Garden, Luoyang city, Henan Province | 34.7068 | 112.3929 |
| 30 | Baotianman Nature Reserve, Nei country, Henan Province | 33.0619 | 111.8647 |
| 31 | Baiyun Mountain National Forest Park, Song country, Henan Province | 33.6856 | 111.8513 |
| 32 | Muzhuwa, Fanpo town, Song country, Henan Province | 33.5678 | 111.7877 |
| 33 | Xixia country, Henan Province | 33.3096 | 111.4721 |
| 34 | Huojia town, xinxiang city, Henan Province | 35.1280 | 113.6956 |
| 35 | School of Aerospace management, Zhengzhou city, Henan Province | 34.7224 | 113.6506 |
| 36 | Hengchong medicine factory, Baokang country, Hubei Province | 31.7193 | 111.1207 |
| 37 | Houping town, Baokang country, Hubei Province | 31.7197 | 113.6867 |
| 38 | Houping town, Baokang country, Hubei Province | 31.7199 | 111.2901 |
| 39 | Siping town, Baokang country, Hubei Province | 31.9563 | 111.1340 |
| 40 | Yunqi Shan, Baokang country, Hubei Province | 31.4337 | 111.2559 |
| 41 | Yunqi Shan, Baokang country, Hubei Province | 31.4502 | 111.2692 |
| 42 | Government, Baokang country, Hubei Province | 31.8738 | 111.2567 |
| 43 | Lijiaping village, Shaoyang city, Hunan Province | 27.0165 | 111.6151 |
| 44 | Phoenix town, Tujia and Miao Autonomous region, Hunan Province | 28.5932 | 110.0025 |
| 45 | Niji town, Cao country, Heze city, Shandong Province | 36.4339 | 115.8343 |
| 46 | Huanggang town, Mudan district, Heze city, Shandong Province | 35.8007 | 116.2622 |
| 47 | Pili village, Mudan district, Heze city, Shandong Province | 35.4120 | 115.9033 |
| 48 | Pili village, Mudan district, Heze city, Shandong Province | 35.4308 | 115.9249 |
| 49 | Xiaoliu town, Mudan district, Heze city, Shandong Province | 35.6123 | 115.7426 |
| 50 | Mei Yuan, Donge country, Liaocheng city, Shandong Province | 36.3342 | 111.3062 |
| 51 | Mei Yuan, Donge country, Liaocheng city, Shandong Province | 36.3530 | 111.2841 |
| 52 | Yuan village, Nantang town, Yicheng country, Yuncheng city, Shanxi Province | 35.6594 | 111.6279 |
| 53 | Yuan village, Nantang town, Yicheng country, Yuncheng city, Shanxi Province | 35.6751 | 111.6409 |
| 54 | Nanfan town, Jian country, Yuncheng city, Shanxi Province | 35.6031 | 111.6170 |
| 55 | Dali village, Yingkou town, Mei country, Baoji city, Shaanxi Province | 34.1334 | 107.8978 |
| 56 | Dali village, Yingkou town, Mei country, Baoji city, Shaanxi Province | 34.1599 | 107.7583 |
| 57 | Dali village, Yingkou town, Mei country, Baoji city, Shaanxi Province | 34.1603 | 107.7652 |
| 58 | Guandi temple, Xuanyuan, Huangling country, Shaanxi Province | 35.5890 | 109.2791 |
| 59 | Shangzhou, Shangluo city, Shaanxi Province | 33.8471 | 109.9044 |
| 60 | Shangluo coutry, Shangluo city, Shaanxi Province | 33.8163 | 110.6281 |
| 61 | Tongguan coutry, Shangluo city, Shaanxi Province | 34.1603 | 110.2536 |
| 62 | Taibai mountain, Taibai county, Shaanxi Province | 34.0495 | 107.4554 |
| 63 | Yang mountain, Gouyuan town, Xunyang country, Shaanxi Province | 32.7899 | 109.5861 |
| 64 | Ganquan country, Yanan city, Shaanxi Province | 36.7362 | 109.1657 |
| 65 | Dali village, Yingkou town, Mei country, Baoji city, Shaanxi Province | 33.2806 | 109.8455 |
| 66 | Miganqiao village, Fengxiang country, Baoji city, Shaanxi Province | 34.4972 | 107.3667 |
| 67 | Huping village, Shanyang country, Shangluo city, Shaanxi Province | 33.2655 | 109.8252 |
| 68 | Heyang country, Weinan city, Shaanxi Province | 35.2258 | 110.1886 |
| 69 | Shanghai Chen Shan Botanical Garden | 31.0812 | 121.1906 |
| 70 | Majia country, Kaijiang country, Dazhou city, Sichuan Province | 31.1739 | 107.7389 |
| 71 | Lushun, Pingchang country, Dazhou city, Sichuan Province | 31.2070 | 107.4604 |
| 72 | Wuping commune, Fengdu town, Puling district, Chongqing city | 29.7087 | 107.3963 |
| 73 | Yangyan commune, Jiangjin district, Chongqing city | 29.2939 | 106.2583 |
| 74 | Yanjing commune, An country, Mianyang city, Sichuan Province | 31.5403 | 104.5723 |
| 75 | Jigong Ling, Fenghuang mountain, Nanchong city, Sichuan Province | 29.5864 | 105.0446 |
| 76 | Zouma town, Nanchong city, Sichuan Province | 30.8061 | 106.2502 |
| 77 | Nanchuan district, Chongqing city, | 29.1607 | 107.0973 |
| 78 | Wuzhi commune, Jianyang town, Neijiang city, Sichuan Province | 29.5846 | 105.0547 |
| 79 | Institute of vegetables and flowers, Chinese Academy of Agricultural Sciences | 40.0382 | 116.2922 |
| 80 | Chinese Academy of Sciences Beijing Botanical Garden | 39.9973 | 116.2207 |
| 81 | Shuanggui village, Chengxi town, Dianjiang country, Chongqing city | 30.2192 | 107.2694 |
| 82 | Bijia mountain, Nanchuan district, Chongqing city, | 29.1292 | 107.0574 |
| 83 | Zhenqian town, Zhenghe county, Nanping city, Fujian Province | 27.2484 | 119.0899 |
| 84 | Liujiaping town, Wen County, Gansu Province | 32.7953 | 104.8241 |
| 85 | Wuding Lion Rock Scenic Area, Chuxiong city, Yunnan Province | 25.5398 | 102.3859 |
| 86 | Kuzhi Peony Park peony research base, Biancang city, Jiangsu Province | 33.2052 | 120.2797 |
| 87 | Shenyang Agricultural University | 41.8273 | 123.5718 |

| **Table S2. Environmental variables used to predict the potential geographic distribution of *P. ostii*** | | | | | | | |
| --- | --- | --- | --- | --- | --- | --- | --- |
| **Code** | **Environmental variables** | **Resolution** | **Scaling Factors** | **Unit** | **Source** |  | |
| Bio1 | Annual mean temperature | 30″× 30″ | 10 | ◦C | http://www.worldclim.org/ | |  |
| Bio2 | Mean diurnal air temperature range | 30″× 30″ | 10 | ◦C | http://www.worldclim.org/ | |  |
| Bio3 | Isothermality ((Bio2/Bio7)×100) | 30″× 30″ | 100 | – | http://www.worldclim.org/ | |  |
| Bio4 | Temperature seasonality | 30″× 30″ | 100 | C of V | http://www.worldclim.org/ | |  |
| Bio5 | Maximum temperature of warmest month | 30″× 30″ | 10 | ◦C | http://www.worldclim.org/ | |  |
| Bio6 | Minimum temperature of coldest month | 30″× 30″ | 10 | ◦C | http://www.worldclim.org/ | |  |
| Bio7 | Temperature annual range (Bio5–Bio6) | 30″× 30″ | 10 | ◦C | http://www.worldclim.org/ | |  |
| Bio8 | Mean temperature of wettest quarter | 30″× 30″ | 10 | ◦C | http://www.worldclim.org/ | |  |
| Bio9 | Mean temperature of driest quarter | 30″× 30″ | 10 | ◦C | http://www.worldclim.org/ | |  |
| Bio10 | Mean temperature of warmest quarter | 30″× 30″ | 10 | ◦C | http://www.worldclim.org/ | |  |
| Bio11 | Mean temperature of coldest quarter | 30″× 30″ | 10 | ◦C | http://www.worldclim.org/ | |  |
| Bio12 | Annual precipitation | 30″× 30″ | 1 | mm | http://www.worldclim.org/ | |  |
| Bio13 | Precipitation of wettest month | 30″× 30″ | 1 | mm | http://www.worldclim.org/ | |  |
| Bio14 | Precipitation of driest month | 30″× 30″ | 1 | mm | http://www.worldclim.org/ | |  |
| Bio15 | Precipitation seasonality (CV) | 30″× 30″ | 100 | C of V | http://www.worldclim.org/ | |  |
| Bio16 | Precipitation of wettest quarter | 30″× 30″ | 1 | mm | http://www.worldclim.org/ | |  |
| Bio17 | Precipitation of driest quarter | 30″× 30″ | 1 | mm | http://www.worldclim.org/ | |  |
| Bio18 | Precipitation of warmest quarter | 30″× 30″ | 1 | mm | http://www.worldclim.org/ | |  |
| Bio19 | Precipitation of coldest quarter | 30″× 30″ | 1 | mm | http://www.worldclim.org/ | |  |
| WET | Wet-day frequency | 0.5°×0.5° | 1 | – | http://www.ipcc-data.org/obs/cru_ts2_1.html | |  |
| VAP | Vapor pressure | 0.5°×0.5° | 1 | hPa | http://www.ipcc-data.org/obs/cru_ts2_1.html | |  |
| FRS | Ground-frost frequency | 0.5°×0.5° | 1 | – | http://www.ipcc-data.org/obs/cru_ts2_1.html | |  |
| SpH | Soil pH | 0.5°×0.5° | 1 | – | http://www.sage.wisc.edu/atlas/index.php | |  |
| SC | Soil organic carbon | 0.5°×0.5° | 1 | – | http://www.sage.wisc.edu/atlas/index.php | |  |
| GDD | Growing degree days | 0.5°×0.5° | 1 | ℃ | http://www.sage.wisc.edu/atlas/index.php | |  |
| UVB-1 | Annual mean UV-B | 30″× 30″ | 1 | J m^-2^ •day^-1^ | http://www.ufz.de/gluv/ | |  |
| UVB-2 | UV-B seasonality | 30″× 30″ | 1 | J m^-2^ •day^-1^ | http://www.ufz.de/gluv/ | |  |
| UVB-3 | mean UV-B of lightest month | 30″× 30″ | 1 | J m^-2^ •day^-1^ | http://www.ufz.de/gluv/ | |  |
| UVB-4 | mean UV-B of lowest month | 30″× 30″ | 1 | J m^-2^ •day^-1^ | http://www.ufz.de/gluv/ | |  |

| **Table S3. Multi-collinearity results of the environmental variables used in the study. Multi-collinearity test was conducted by Arcgis 10.0 using multivariate band collection statistics to examine the cross-correlation** | | | | | | | | | | | | | | | |
| --- | --- | --- | --- | --- | --- | --- | --- | --- | --- | --- | --- | --- | --- | --- | --- |
| Layer | Altitude | Bio1 | Bio2 | Bio3 | Bio4 | Bio5 | Bio6 | Bio7 | Bio8 | Bio9 | Bio10 | Bio11 | Bio12 | Bio13 | Bio14 |
| Altitude | 100 | -42.82 | -70.61 | -24.54 | -25.31 | -22.85 | -24.32 | 20.20 | -22.37 | -24.24 | -15.42 | -14.22 | 31.48 | 21.25 | -13.65 |
| Bio1 | -42.82 | 100 | 90.75 | 97.3 | 52.2 | 54.44 | 8.32 | 20.33 | 54.36 | 14.23 | 27.01 | 18.17 | -21.44 | 67.12 | -78.16 |
| Bio2 | -70.61 | 90.75 | 100 | 78.85 | 34.99 | 38.02 | 7.71 | 11.49 | 37.21 | 11.37 | 12.98 | 13.90 | -15.06 | 36.82 | -44.95 |
| Bio3 | -24.54 | 97.3 | 78.85 | 100 | 57.07 | 58.59 | 9.06 | 22.55 | 58.85 | 15.58 | 31.10 | 20.19 | -23.86 | 77.98 | -90.37 |
| Bio4 | -25.31 | 52.2 | 34.99 | 57.07 | 100 | 93.23 | 46.92 | 1.33 | 95.61 | 53.71 | 82.98 | 42.07 | -59.58 | 41.83 | -58.28 |
| Bio5 | -22.85 | 54.44 | 38.02 | 58.59 | 93.23 | 100.00 | 22.22 | 25.66 | 99.13 | 29.21 | 74.56 | 27.46 | -42.78 | 49.52 | -58.54 |
| Bio6 | -24.32 | 8.32 | 7.71 | 9.06 | 46.92 | 22.22 | 100.00 | -53.16 | 24.69 | 98.50 | 39.38 | 68.63 | -62.73 | -20.66 | -7.40 |
| Bio7 | 20.2 | 20.33 | 11.49 | 22.55 | 1.33 | 25.66 | -53.16 | 100.00 | 22.02 | -51.57 | 3.67 | -36.66 | 49.32 | 45.96 | -25.36 |
| Bio8 | -22.37 | 54.36 | 37.21 | 58.85 | 95.61 | 99.13 | 24.69 | 22.02 | 100.00 | 31.81 | 77.41 | 27.74 | -45.21 | 49.36 | -59.44 |
| Bio9 | -24.24 | 14.23 | 11.37 | 15.58 | 53.71 | 29.21 | 98.50 | -51.57 | 31.81 | 100.00 | 44.77 | 70.66 | -65.62 | -14.28 | -14.27 |
| Bio10 | -15.42 | 27.01 | 12.98 | 31.1 | 82.98 | 74.56 | 39.38 | 3.67 | 77.41 | 44.77 | 100.00 | 21.70 | -50.56 | 23.25 | -35.70 |
| Bio11 | -14.22 | 18.17 | 13.9 | 20.19 | 42.07 | 27.46 | 68.63 | -36.66 | 27.74 | 70.66 | 21.70 | 100.00 | -43.81 | 0.38 | -19.49 |
| Bio12 | 31.48 | -21.44 | -15.06 | -23.86 | -59.58 | -42.78 | -62.73 | 49.32 | -45.21 | -65.62 | -50.56 | -43.81 | 100.00 | 15.42 | 23.80 |
| Bio13 | 21.25 | 67.12 | 36.82 | 77.98 | 41.83 | 49.52 | -20.66 | 45.96 | 49.36 | -14.28 | 23.25 | 0.38 | 15.42 | 100.00 | -87.65 |
| Bio14 | -13.65 | -78.16 | -44.95 | -90.37 | -58.28 | -58.54 | -7.40 | -25.36 | -59.44 | -14.27 | -35.70 | -19.49 | 23.80 | -87.65 | 100.00 |
| Bio15 | -70.31 | 85.49 | 98.27 | 72.27 | 23.27 | 28.34 | -0.36 | 12.49 | 27.24 | 2.60 | -0.71 | 9.34 | -2.16 | 33.15 | -36.67 |
| Bio16 | -27.39 | 96.92 | 79.02 | 99.52 | 59.31 | 59.04 | 14.85 | 15.82 | 59.48 | 21.36 | 32.80 | 24.61 | -31.78 | 73.84 | -89.51 |
| Bio17 | -8.78 | -75.89 | -43.17 | -88.07 | -65.45 | -61.77 | -20.43 | -13.35 | -63.09 | -27.35 | -45.09 | -27.37 | 41.82 | -78.75 | 97.77 |
| Bio18 | -62.53 | 70.4 | 76.56 | 60.71 | 40.18 | 43.91 | 9.17 | 24.39 | 43.06 | 12.12 | 32.32 | -3.88 | -18.86 | 29.96 | -34.73 |
| Bio19 | -23.98 | 90.64 | 77.18 | 91.6 | 42.24 | 43.49 | 4.30 | 11.44 | 43.70 | 10.38 | 13.53 | 25.55 | -15.15 | 68.49 | -79.39 |
| FRS | 1.17 | -2.19 | -1.75 | -2.27 | -2.35 | -2.35 | -1.11 | -0.17 | -2.34 | -1.20 | -1.16 | -1.34 | 1.73 | -1.64 | 2.07 |
| GDD | -45.95 | 92.02 | 85.17 | 88.5 | 50.79 | 55.68 | 1.55 | 25.23 | 55.35 | 7.35 | 23.17 | 12.13 | -17.37 | 64.21 | -69.20 |
| SC | -6.66 | -25.64 | -25.65 | -23.9 | 14.51 | 10.34 | 18.65 | -15.67 | 10.93 | 18.71 | 19.63 | 6.09 | -23.41 | -20.38 | 16.95 |
| SpH | 1.89 | 1.97 | 15.39 | -4.57 | -57.05 | -47.86 | -32.90 | 5.83 | -50.45 | -37.07 | -59.10 | -21.08 | 38.56 | -11.56 | 17.30 |
| UVB1 | 43.08 | 41.45% | 13.14 | 53.49 | 20.52 | 25.29 | -15.30 | 32.17 | 25.40 | -10.53 | 7.53 | 1.29 | 9.56 | 69.52 | -68.57 |
| UVB2 | 73.2 | -28.3 | -41.78 | -18.74 | -44.1 | -40.82 | -26.08 | 10.23 | -41.31 | -27.05 | -36.15 | -10.01 | 40.55 | 7.16 | -1.96 |
| UVB3 | 64.29 | 23.79 | -5.3 | 37.76 | 0.84 | 6.50 | -23.13 | 31.64 | 6.37 | -19.44 | -9.05 | -2.44 | 23.67 | 61.59 | -58.63 |
| UVB4 | 13.57 | 50.97 | 28.4 | 59.2 | 37.71 | 41.35 | -5.69 | 27.38 | 41.62 | -0.65 | 20.51 | 5.00 | -6.51 | 65.76 | -66.23 |
| VAP | -0.08 | 0.24 | 0.43 | 0.1 | -0.64 | -0.68 | -0.59 | 0.15 | -0.65 | -0.51 | -0.06 | -0.71 | 0.73 | -0.12 | 0.16 |
| WET | 0.49 | -1.02 | -0.76 | -1.09 | -1.2 | -1.37 | -0.37 | -0.42 | -1.32 | -0.38 | -0.30 | -0.70 | 0.76 | -1.00 | 1.06 |
|  |  |  |  |  |  |  |  |  |  |  |  |  |  |  |  |
| **Table S3. Multi-collinearity results of the environmental variables used in the study. Multi-collinearity test was conducted by Arcgis 10.0 using multivariate band collection statistics to examine the cross-correlation** | | | | | | | | | | | | | | | |
| Layer | Bio15 | Bio16 | Bio17 | Bio18 | Bio19 | FRS | GDD | SC | SpH | UVB1 | UVB2 | UVB3 | UVB4 | VAP | WET |
| Altitude | -70.31 | -27.39 | -8.78 | -62.53 | -23.98 | 1.17 | -45.95 | -6.66 | 1.89 | 43.08 | 73.20 | 64.29 | 13.57 | -0.08 | 0.49 |
| Bio1 | 85.49 | 96.92 | -75.89 | 70.40 | 90.64 | -2.19 | 92.02 | -25.64 | 1.97 | 41.45 | -28.30 | 23.79 | 50.97 | 0.24 | -1.02 |
| Bio2 | 98.27 | 79.02 | -43.17 | 76.56 | 77.18 | -1.75 | 85.17 | -25.65 | 15.39 | 13.14 | -41.78 | -5.30 | 28.40 | 0.43 | -0.76 |
| Bio3 | 72.27 | 99.52 | -88.07 | 60.71 | 91.60 | -2.27 | 88.50 | -23.90 | -4.57 | 53.49 | -18.74 | 37.76 | 59.20 | 0.10 | -1.09 |
| Bio4 | 23.27 | 59.31 | -65.45 | 40.18 | 42.24 | -2.35 | 50.79 | 14.51 | -57.05 | 20.52 | -44.10 | 0.84 | 37.71 | -0.64 | -1.20 |
| Bio5 | 28.34 | 59.04 | -61.77 | 43.91 | 43.49 | -2.35 | 55.68 | 10.34 | -47.86 | 25.29 | -40.82 | 6.50 | 41.35 | -0.68 | -1.37 |
| Bio6 | -0.36 | 14.85 | -20.43 | 9.17 | 4.30 | -1.11 | 1.55 | 18.65 | -32.90 | -15.30 | -26.08 | -23.13 | -5.69 | -0.59 | -0.37 |
| Bio7 | 12.49 | 15.82 | -13.35 | 24.39 | 11.44 | -0.17 | 25.23 | -15.67 | 5.83 | 32.17 | 10.23 | 31.64 | 27.38 | 0.15 | -0.42 |
| Bio8 | 27.24 | 59.48 | -63.09 | 43.06 | 43.70 | -2.34 | 55.35 | 10.93 | -50.45 | 25.40 | -41.31 | 6.37 | 41.62 | -0.65 | -1.32 |
| Bio9 | 2.60 | 21.36 | -27.35 | 12.12 | 10.38 | -1.20 | 7.35 | 18.71 | -37.07 | -10.53 | -27.05 | -19.44 | -0.65 | -0.51 | -0.38 |
| Bio10 | -0.71 | 32.80 | -45.09 | 32.32 | 13.53 | -1.16 | 23.17 | 19.63 | -59.10 | 7.53 | -36.15 | -9.05 | 20.51 | -0.06 | -0.30 |
| Bio11 | 9.34 | 24.61 | -27.37 | -3.88 | 25.55 | -1.34 | 12.13 | 6.09 | -21.08 | 1.29 | -10.01 | -2.44 | 5.00 | -0.71 | -0.70 |
| Bio12 | -2.16 | -31.78 | 41.82 | -18.86 | -15.15 | 1.73 | -17.37 | -23.41 | 38.56 | 9.56 | 40.55 | 23.67 | -6.51 | 0.73 | 0.76 |
| Bio13 | 33.15 | 73.84 | -78.75 | 29.96 | 68.49 | -1.64 | 64.21 | -20.38 | -11.56 | 69.52 | 7.16 | 61.59 | 65.76 | -0.12 | -1.00 |
| Bio14 | -36.67 | -89.51 | 97.77 | -34.73 | -79.39 | 2.07 | -69.20 | 16.95 | 17.30 | -68.57 | -1.96 | -58.63 | -66.23 | 0.16 | 1.06 |
| Bio15 | 100.00 | 71.82 | -32.24 | 71.14 | 74.37 | -1.43 | 81.91 | -28.27 | 22.86 | 9.90 | -38.23 | -6.28 | 24.09 | 0.56 | -0.60 |
| Bio16 | 71.82 | 100.00 | -89.02 | 59.98 | 91.59 | -2.34 | 87.62 | -22.46 | -5.67 | 50.64 | -21.12 | 34.44 | 57.33 | 0.04 | -1.11 |
| Bio17 | -32.24 | -89.02 | 100.00 | -35.02 | -75.92 | 2.25 | -65.58 | 12.04 | 22.69 | -62.40 | 3.71 | -50.97 | -62.23 | 0.31 | 1.13 |
| Bio18 | 71.14 | 59.98 | -35.02 | 100.00 | 38.73 | -1.46 | 68.76 | -6.42 | -4.25 | 3.84 | -53.07 | -17.75 | 23.80 | 0.34 | -0.64 |
| Bio19 | 74.37 | 91.59 | -75.92 | 38.73 | 100.00 | -1.99 | 81.48 | -30.96 | 8.32 | 49.29 | -6.92 | 38.92 | 50.52 | 0.15 | -0.94 |
| FRS | -1.43 | -2.34 | 2.25 | -1.46 | -1.99 | 100.00 | -4.33 | 0.14 | 0.91 | -29.37 | 29.18 | -13.65 | -38.95 | 99.97 | 99.99 |
| GDD | 81.91 | 87.62 | -65.58 | 68.76 | 81.48 | -4.33 | 100.00 | -21.71 | -3.42 | 38.30 | -40.24 | 17.59 | 53.43 | -1.89 | -3.21 |
| SC | -28.27 | -22.46 | 12.04 | -6.42 | -30.96 | 0.14 | -21.71 | 100.00 | -42.08 | -20.23 | -19.45 | -23.91 | -11.37 | -0.28 | 0.15 |
| SpH | 22.86 | -5.67 | 22.69 | -4.25 | 8.32 | 0.91 | -3.42 | -42.08 | 100.00 | -0.75 | 34.77 | 11.65 | -15.36 | 0.46 | 0.30 |
| UVB1 | 9.90 | 50.64 | -62.40 | 3.84 | 49.29 | -29.37 | 38.30 | -20.23 | -0.75 | 100.00 | 18.60 | 92.64 | 91.05 | -28.47 | -29.03 |
| UVB2 | -38.23 | -21.12 | 3.71 | -53.07 | -6.92 | 29.18 | -40.24 | -19.45 | 34.77 | 18.60 | 100.00 | 52.76 | -22.82 | 28.04 | 28.44 |
| UVB3 | -6.28 | 34.44 | -50.97 | -17.75 | 38.92 | -13.65 | 17.59 | -23.91 | 11.65 | 92.64 | 52.76 | 100.00 | 70.29 | -13.33 | -13.64 |
| UVB4 | 24.09 | 57.33 | -62.23 | 23.80 | 50.52 | -38.95 | 53.43 | -11.37 | -15.36 | 91.05 | -22.82 | 70.29 | 100.00 | -37.63 | -38.33 |
| VAP | 0.56 | 0.04 | 0.31 | 0.34 | 0.15 | 99.97 | -1.89 | -0.28 | 0.46 | -28.47 | 28.04 | -13.33 | -37.63 | 100.00 | 99.99 |
| WET | -0.60 | -1.11 | 1.13 | -0.64 | -0.94 | 99.99 | -3.21 | 0.15 | 0.30 | -29.03 | 28.44 | -13.64 | -38.33 | 99.99 | 100.00 |

**Table S4. Areas of highly suitable, moderately suitable, marginally suitable, and unsuitable habitats in every county or district in China**

| Province | County/District | Unsuitable habitat | Marginally suitable habitat | Moderate suitable habitat | Highly suitable habitat |
| --- | --- | --- | --- | --- | --- |
| Anhui | Anqing District | 3.72 | 427.34 | 31.96 | 0.00 |
| Anhui | Bengbu District | 0.00 | 188.03 | 220.73 | 0.74 |
| Anhui | Bozhou | 0.00 | 0.00 | 139.72 | 2,198.37 |
| Anhui | Chaohu | 0.00 | 3.72 | 1,423.96 | 662.93 |
| Anhui | Chuzhou | 0.00 | 610.16 | 807.11 | 2.97 |
| Anhui | Congyang | 15.61 | 1,650.63 | 232.62 | 5.20 |
| Anhui | Dangtu | 0.00 | 899.26 | 573.00 | 28.24 |
| Anhui | Dangshan | 0.00 | 0.00 | 1,256.00 | 0.00 |
| Anhui | Dingyuan | 0.00 | 277.95 | 2,683.67 | 141.21 |
| Anhui | Dongzhi | 1,537.67 | 1,698.94 | 2.97 | 0.00 |
| Anhui | Fanchang | 0.00 | 0.00 | 700.09 | 219.99 |
| Anhui | Feidong | 0.00 | 28.24 | 1,841.63 | 451.86 |
| Anhui | Feixi | 0.00 | 1034.53 | 1,340.72 | 0.00 |
| Anhui | Fengtai | 0.00 | 20.81 | 1,088.04 | 0.00 |
| Anhui | Fengyang | 0.00 | 504.63 | 1,405.38 | 113.71 |
| Anhui | Funan | 0.00 | 322.55 | 1,677.39 | 0.00 |
| Anhui | Fuyang District | 0.00 | 0.00 | 63.91 | 0.00 |
| Anhui | Guzheng | 0.00 | 943.11 | 598.27 | 0.00 |
| Anhui | Guangde | 0.00 | 144.18 | 1,615.70 | 400.58 |
| Anhui | Guichi | 31.96 | 2,039.32 | 505.37 | 0.74 |
| Anhui | Hanshan | 0.00 | 0.00 | 719.41 | 350.79 |
| Anhui | Hefei District | 0.00 | 211.07 | 311.40 | 0.00 |
| Anhui | Hexian | 0.00 | 427.34 | 1,068.71 | 81.01 |
| Anhui | Huaining | 0.00 | 1,050.13 | 572.26 | 0.00 |
| Anhui | Huaiyuan | 0.00 | 508.34 | 1,993.99 | 5.20 |
| Anhui | Huaibei District | 0.00 | 0.00 | 325.52 | 17.84 |
| Anhui | Huainan District | 0.00 | 282.41 | 783.33 | 17.09 |
| Anhui | Huangshan District | 622.80 | 1,263.43 | 346.33 | 55.00 |
| Anhui | Huoqiu | 0.00 | 2,784.75 | 1,071.69 | 36.42 |
| Anhui | Huoshan | 0.00 | 0.00 | 605.70 | 1,454.43 |
| Anhui | Jixi | 11.15 | 1,094.72 | 1.49 | 0.00 |
| Anhui | Jieshou | 0.00 | 0.00 | 665.16 | 3.72 |
| Anhui | Jinzhai | 0.00 | 0.00 | 721.64 | 3,262.62 |
| Anhui | Jingxian | 0.00 | 0.00 | 416.19 | 1,633.54 |
| Anhui | Jingde | 0.00 | 566.31 | 327.01 | 18.58 |
| Anhui | Laian | 0.00 | 947.57 | 592.33 | 0.00 |
| Anhui | Langxi | 0.00 | 442.20 | 601.99 | 95.87 |
| Anhui | Lixin | 0.00 | 2.97 | 2,132.22 | 0.00 |
| Anhui | Linquan | 0.00 | 1.49 | 1,701.17 | 208.84 |
| Anhui | Linbi | 0.00 | 563.34 | 1,623.88 | 5.20 |
| Anhui | Luan | 0.00 | 11.89 | 34.93 | 0.00 |
| Anhui | Lujiang | 0.00 | 741.71 | 1,506.45 | 149.38 |
| Anhui | Maanshan District | 0.00 | 147.90 | 154.58 | 6.69 |
| Anhui | Mengcheng | 0.00 | 16.35 | 2,219.92 | 0.00 |
| Anhui | Mingguang | 0.00 | 1,082.83 | 1,348.90 | 8.92 |
| Anhui | Nanling | 0.00 | 0.00 | 713.47 | 555.17 |
| Anhui | Ningguo | 0.00 | 1,043.44 | 1,319.17 | 73.58 |
| Anhui | Qimen | 2115.13 | 91.41 | 0.00 | 0.00 |
| Anhui | Qianshan | 0.00 | 303.97 | 827.92 | 590.10 |
| Anhui | Qingyang | 0.00 | 274.98 | 617.59 | 308.43 |
| Anhui | Quanjiao | 0.00 | 451.86 | 1,002.57 | 166.48 |
| Anhui | Shitai | 725.36 | 755.08 | 0.00 | 0.00 |
| Anhui | Shouxian | 0.00 | 2781.77 | 240.05 | 5.95 |
| Anhui | Shucheng | 0.00 | 264.58 | 1,239.65 | 631.71 |
| Anhui | Sixian | 0.00 | 1,035.27 | 898.52 | 0.00 |
| Anhui | Suixi | 0.00 | 55.00 | 2,397.54 | 57.23 |
| Anhui | Taihe | 0.00 | 0.00 | 1,673.67 | 248.23 |
| Anhui | Taihu | 0.00 | 413.22 | 1,455.17 | 170.93 |
| Anhui | Tiancheng | 0.00 | 955.75 | 847.98 | 2.97 |
| Anhui | Tongcheng | 0.00 | 1,083.58 | 306.20 | 272.75 |
| Anhui | Tongling District | 0.00 | 39.39 | 33.44 | 7.43 |
| Anhui | Tongling | 0.00 | 309.91 | 419.16 | 237.82 |
| Anhui | Wangjiang | 263.83 | 1,018.18 | 53.51 | 0.00 |
| Anhui | Woyang | 0.00 | 0.00 | 2,020.74 | 181.34 |
| Anhui | Wuwei | 0.00 | 734.28 | 1,595.64 | 114.45 |
| Anhui | Wuhu District | 0.00 | 0.74 | 148.64 | 0.00 |
| Anhui | Wuhu | 0.00 | 180.60 | 796.70 | 9.66 |
| Anhui | Wuhe | 0.00 | 1,085.81 | 557.40 | 1.49 |
| Anhui | Shexian | 940.14 | 1,238.16 | 1,742.05 | 172.42 |
| Anhui | Xiaoxian | 0.00 | 0.00 | 1,742.05 | 172.42 |
| Anhui | Xiuning | 1,695.22 | 420.65 | 0.00 | 0.00 |
| Anhui | Suzsong | 49.05 | 1,672.19 | 482.33 | 149.38 |
| Anhui | Suzhou District | 0.00 | 588.61 | 2,411.66 | 72.83 |
| Anhui | Xuanzhou District | 0.00 | 700.09 | 1,606.78 | 353.02 |
| Anhui | Yixian | 703.06 | 150.87 | 0.00 | 0.00 |
| Anhui | Yingshang | 0.00 | 376.80 | 1,678.87 | 0.00 |
| Anhui | Yuexi | 0.00 | 50.54 | 1,243.36 | 1,116.28 |
| Anhui | Changfeng | 0.00 | 1,053.85 | 1,378.62 | 0.00 |
| Beijing | Beijing District | 4.60 | 4,254.83 | 529.22 | 0.00 |
| Beijing | Changping | 0.00 | 1,290.52 | 55.22 | 0.00 |
| Beijing | Daxing | 0.00 | 1,009.80 | 0.00 | 0.00 |
| Beijing | Huairou | 336.60 | 1,807.25 | 0.00 | 0.00 |
| Beijing | Miyun | 0.00 | 2,211.56 | 0.66 | 0.00 |
| Beijing | Pinggu | 0.00 | 894.75 | 59.17 | 0.00 |
| Beijing | Shunyi | 0.00 | 1,010.46 | 0.00 | 0.00 |
| Beijing | Tongzhou | 0.00 | 927.62 | 0.00 | 0.00 |
| Beijing | Yanqing | 21.04 | 1,997.90 | 0.00 | 0.00 |
| Chongqing | Banan District | 0.00 | 547.65 | 1,143.97 | 156.03 |
| Chongqing | Beipei District | 0.00 | 281.16 | 368.45 | 169.93 |
| Chongqing | Bishan | 0.00 | 889.84 | 67.97 | 0.00 |
| Chongqing | Dadukou District | 0.00 | 109.69 | 12.36 | 0.00 |
| Chongqing | Dazu | 0.00 | 1,223.53 | 203.15 | 0.00 |
| Chongqing | Dianjiang | 0.00 | 61.79 | 1,459.89 | 54.84 |
| Chongqing | Fengdu | 2.32 | 1,353.30 | 1,428.23 | 219.37 |
| Chongqing | Fengjie | 322.88 | 3,771.78 | 233.27 | 0.00 |
| Chongqing | Puling District | 0.00 | 329.06 | 1,443.67 | 1,274.51 |
| Chongqing | Hechuan City | 0.00 | 414.02 | 1,931.85 | 75.70 |
| Chongqing | Jiangbei District | 0.00 | 98.10 | 124.36 | 11.59 |
| Chongqing | Jiangjin City | 122.04 | 2,803.92 | 368.45 | 0.00 |
| Chongqing | Jiulongpo District | 0.00 | 380.81 | 57.16 | 0.00 |
| Chongqing | Kai | 794.06 | 3,151.52 | 264.94 | 0.00 |
| Chongqing | Liangping | 0.00 | 887.52 | 1,036.60 | 44.80 |
| Chongqing | Nanan District | 0.00 | 149.08 | 136.72 | 16.22 |
| Chongqing | Nanchuan City | 0.00 | 1,479.21 | 1,082.18 | 124.36 |
| Chongqing | Pengshui Miao and Tujia Autonomous County | 2,150.45 | 1,896.32 | 0.00 | 0.00 |
| Chongqing | Qijiang | 594.00 | 1,401.96 | 238.68 | 0.00 |
| Chongqing | Qianjiang Tujia and Miao Autonomous County | 855.85 | 3,269.70 | 1,815.99 | 27.04 |
| Chongqing | Rongchang | 0.00 | 1,113.07 | 9.27 | 0.00 |
| Chongqing | Shapingba District | 0.00 | 363.82 | 74.15 | 0.00 |
| Chongqing | Shizhu Tujia Autonomous County | 128.22 | 2372.91 | 636.48 | 0.00 |
| Chongqing | Shuangqiao District | 0.00 | 38.62 | 8.50 | 0.00 |
| Chongqing | Tongliang | 0.00 | 1,210.40 | 186.93 | 0.00 |
| Chongqing | Tongnan | 0.00 | 199.29 | 1,473.80 | 1.54 |
| Chongqing | Wansheng District | 90.37 | 454.19 | 56.39 | 0.00 |
| Chongqing | Wanzhou District | 111.23 | 132.09 | 0.00 | 0.00 |
| Chongqing | Wushan | 129.00 | 1,635.24 | 1,364.89 | 0.00 |
| Chongqing | Wuxi | 10.81 | 1,806.72 | 2,371.36 | 50.98 |
| Chongqing | Wulong | 346.05 | 1,914.86 | 702.91 | 16.99 |
| Chongqing | Xiushan Tujia and Miao Autonomous County | 1,765.01 | 736.90 | 0.00 | 0.00 |
| Chongqing | Yongchuan City | 6.18 | 1,509.33 | 111.23 | 0.00 |
| Chongqing | Youyang Tujia and Miao Autonomous County | 2,163.58 | 3,128.35 | 0.00 | 0.00 |
| Chongqing | Yu Bei District | 0.00 | 284.25 | 876.71 | 293.52 |
| Chongqing | Yunyang | 2,372.91 | 1,455.26 | 0.00 | 0.00 |
| Chongqing | Changshou | 0.00 | 45.57 | 1,251.34 | 171.48 |
| Chongqing | Zhong | 0.77 | 909.92 | 1,316.22 | 59.48 |
| Fujian | Anxi | 3,047.46 | 23.60 | 0.00 | 0.00 |
| Fujian | Datian | 2,340.94 | 0.00 | 0.00 | 0.00 |
| Fujian | Dehua | 2,349.08 | 0.00 | 0.00 | 0.00 |
| Fujian | Dongshan | 171.75 | 0.00 | 0.00 | 0.00 |
| Fujian | Fuan | 1,882.68 | 0.00 | 0.00 | 0.00 |
| Fujian | Fuding | 1,502.57 | 0.00 | 0.00 | 0.00 |
| Fujian | Fuqing | 1,150.12 | 0.00 | 0.00 | 0.00 |
| Fujian | Fuzhou District | 983.26 | 0.00 | 0.00 | 0.00 |
| Fujian | Gutian | 2,528.15 | 0.00 | 0.00 | 0.00 |
| Fujian | Guangze | 2,405.25 | 30.12 | 0.00 | 0.00 |
| Fujian | Huaan | 1,384.54 | 0.00 | 0.00 | 0.00 |
| Fujian | Huian | 923.84 | 0.00 | 0.00 | 0.00 |
| Fujian | Jianning | 1,855.01 | 0.00 | 0.00 | 0.00 |
| Fujian | Jianou | 4,451.53 | 0.00 | 0.00 | 0.00 |
| Fujian | Jianyang | 3,657.93 | 0.00 | 0.00 | 0.00 |
| Fujian | Jiangle | 2,401.18 | 0.00 | 0.00 | 0.00 |
| Fujian | Jinmen | 8.14 | 0.00 | 0.00 | 0.00 |
| Fujian | Jinjiang | 651.17 | 0.00 | 0.00 | 0.00 |
| Fujian | Liancheng | 2,743.04 | 0.00 | 0.00 | 0.00 |
| Fujian | Lianjiang | 498.96 | 0.00 | 0.00 | 0.00 |
| Fujian | Longhai | 1,239.66 | 0.00 | 0.00 | 0.00 |
| Fujian | Longyan | 2,830.13 | 0.00 | 0.00 | 0.00 |
| Fujian | Luoyuan | 861.98 | 0.00 | 0.00 | 0.00 |
| Fujian | Minhou | 2,243.27 | 0.00 | 0.00 | 0.00 |
| Fujian | Minqing | 1,593.73 | 0.00 | 0.00 | 0.00 |
| Fujian | mingxi | 1,829.78 | 0.00 | 0.00 | 0.00 |
| Fujian | Nanan District | 2,072.34 | 5.70 | 0.00 | 0.00 |
| Fujian | Nanjing | 2,062.57 | 0.00 | 0.00 | 0.00 |
| Fujian | Nanping District | 2,813.04 | 0.00 | 0.00 | 0.00 |
| Fujian | Ningde District | 1,398.38 | 0.00 | 0.00 | 0.00 |
| Fujian | Ninghua | 2,491.52 | 0.00 | 0.00 | 0.00 |
| Fujian | Pinghe | 2,392.22 | 0.00 | 0.00 | 0.00 |
| Fujian | Pingnan | 1,586.40 | 0.00 | 0.00 | 0.00 |
| Fujian | Putian District | 41.51 | 0.00 | 0.00 | 0.00 |
| Fujian | Putian | 1,184.31 | 0.00 | 0.00 | 0.00 |
| Fujian | Pucheng | 3,643.27 | 0.00 | 0.00 | 0.00 |
| Fujian | Qingliu | 1,933.15 | 0.00 | 0.00 | 0.00 |
| Fujian | Quanzhou District | 547.79 | 0.00 | 0.00 | 0.00 |
| Fujian | Sanming District | 1,214.42 | 0.00 | 0.00 | 0.00 |
| Fujian | Sha | 1,957.57 | 0.00 | 0.00 | 0.00 |
| Fujian | Xiamen District | 423.26 | 0.00 | 0.00 | 0.00 |
| Fujian | Shanghang | 3,005.13 | 0.00 | 0.00 | 0.00 |
| Fujian | Shaowu District | 3,012.46 | 0.00 | 0.00 | 0.00 |
| Fujian | Shishi District | 151.40 | 0.00 | 0.00 | 0.00 |
| Fujian | Shouning | 1,534.31 | 0.00 | 0.00 | 0.00 |
| Fujian | Shunchang | 2,113.03 | 0.00 | 0.00 | 0.00 |
| Fujian | Songxi | 1,120.82 | 0.00 | 0.00 | 0.00 |
| Fujian | Taining | 1,651.52 | 0.00 | 0.00 | 0.00 |
| Fujian | Tongan District | 956.40 | 13.02 | 0.00 | 0.00 |
| Fujian | Wuping | 2,769.08 | 0.00 | 0.00 | 0.00 |
| Fujian | Wuyishan District | 2,966.06 | 44.77 | 0.00 | 0.00 |
| Fujian | Xiapu District | 1,016.63 | 0.00 | 0.00 | 0.00 |
| Fujian | Xianyou | 1,902.22 | 0.00 | 0.00 | 0.00 |
| Fujian | Yongan District | 3,110.13 | 0.00 | 0.00 | 0.00 |
| Fujian | Yongchun | 1,552.22 | 0.00 | 0.00 | 0.00 |
| Fujian | Yongding | 2,324.66 | 0.00 | 0.00 | 0.00 |
| Fujian | Yongtai | 2,385.71 | 0.00 | 0.00 | 0.00 |
| Fujian | Youxi | 3,600.13 | 0.00 | 0.00 | 0.00 |
| Fujian | Yunxiao | 1,039.42 | 0.00 | 0.00 | 0.00 |
| Fujian | Zhangping | 3,106.88 | 0.00 | 0.00 | 0.00 |
| Fujian | Zhangpu | 1,684.08 | 0.00 | 0.00 | 0.00 |
| Fujian | Zhangzhou District | 271.86 | 0.00 | 0.00 | 0.00 |
| Fujian | Changle | 196.16 | 0.00 | 0.00 | 0.00 |
| Fujian | Changtai | 923.03 | 0.81 | 0.00 | 0.00 |
| Fujian | Changting | 3,277.81 | 0.00 | 0.00 | 0.00 |
| Fujian | Zhaoan | 1,291.75 | 0.00 | 0.00 | 0.00 |
| Fujian | Tuorong | 594.19 | 0.00 | 0.00 | 0.00 |
| Fujian | Zhenghe | 1,872.92 | 0.00 | 0.00 | 0.00 |
| Fujian | Zhouning | 1,124.08 | 0.00 | 0.00 | 0.00 |
| Gansu | Aksai Kazak Autonomous County | 9,632.64 | 0.00 | 0.00 | 0.00 |
| Gansu | Anxi | 3,1159.46 | 0.00 | 0.00 | 0.00 |
| Gansu | Baiyin District | 4,038.08 | 0.00 | 0.00 | 0.00 |
| Gansu | Chengxian | 0.00 | 919.17 | 1,037.66 | 0.00 |
| Gansu | Chongxin | 0.00 | 0.00 | 1,020.73 | 19.47 |
| Gansu | Dangchang | 2,753.27 | 1,190.86 | 0.00 | 0.00 |
| Gansu | Diebu | 5,704.60 | 0.00 | 0.00 | 0.00 |
| Gansu | Dingxi | 4,412.18 | 0.00 | 0.00 | 0.00 |
| Gansu | Dongxiang Autonomous County | 1,857.80 | 0.00 | 0.00 | 0.00 |
| Gansu | Dunhuang | 32927.55 | 0.00 | 0.00 | 0.00 |
| Gansu | Gangu | 0.00 | 1,897.58 | 2.54 | 0.00 |
| Gansu | Gaolan | 3,118.91 | 0.00 | 0.00 | 0.00 |
| Gansu | Gaotai | 5,441.37 | 0.00 | 0.00 | 0.00 |
| Gansu | Gulang | 6,307.22 | 0.00 | 0.00 | 0.00 |
| Gansu | Guanghe | 626.32 | 0.00 | 0.00 | 0.00 |
| Gansu | Heshui | 0.00 | 306.39 | 3,321.19 | 0.85 |
| Gansu | Hezheng | 1,130.76 | 0.00 | 0.00 | 0.00 |
| Gansu | Huachi | 27.08 | 4,455.34 | 198.90 | 0.00 |
| Gansu | Huating | 0.00 | 101.57 | 1,317.81 | 0.00 |
| Gansu | Huangxian | 3,095.21 | 8,311.45 | 0.00 | 0.00 |
| Gansu | Huixian | 0.00 | 154.89 | 3,003.80 | 140.50 |
| Gansu | Huining | 6,560.29 | 349.55 | 0.00 | 0.00 |
| Gansu | Jishishan Baoan Dongxiang Salar Autonomous Region | 1,121.45 | 1.69 | 0.00 | 0.00 |
| Gansu | Jiayuguan District | 1,668.21 | 0.00 | 0.00 | 0.00 |
| Gansu | Jinchang District | 1,515.87 | 0.00 | 0.00 | 0.00 |
| Gansu | Jinta | 19,348.23 | 0.00 | 0.00 | 0.00 |
| Gansu | Jingchuan | 0.00 | 0.00 | 1,128.22 | 666.10 |
| Gansu | Jingtai | 6,700.79 | 0.00 | 0.00 | 0.00 |
| Gansu | Jinyuan | 6,929.31 | 0.00 | 0.00 | 0.00 |
| Gansu | Jingning | 60.94 | 2,575.53 | 16.08 | 0.00 |
| Gansu | Jiuquan | 4,454.49 | 0.00 | 0.00 | 0.00 |
| Gansu | Kangle | 1,206.94 | 0.00 | 0.00 | 0.00 |
| Gansu | Kang | 0.00 | 1,590.35 | 1,898.43 | 0.00 |
| Gansu | Langzhou District | 2070.24 | 0.00 | 0.00 | 0.00 |
| Gansu | Li | 258.99 | 4,892.07 | 7.62 | 0.00 |
| Gansu | Liangdang | 0.00 | 0.00 | 704.19 | 964.87 |
| Gansu | Lintan | 1,757.93 | 0.00 | 0.00 | 0.00 |
| Gansu | Lintao | 3,480.31 | 0.00 | 0.00 | 0.00 |
| Gansu | Linxia District | 115.11 | 27.08 | 0.00 | 0.00 |
| Gansu | Linxia | 1,333.05 | 80.41 | 0.00 | 0.00 |
| Gansu | Linze | 3,454.92 | 0.00 | 0.00 | 0.00 |
| Gansu | Lingtai | 0.00 | 0.00 | 179.43 | 2,183.66 |
| Gansu | Longxi | 2,398.64 | 505.29 | 0.00 | 0.00 |
| Gansu | Luqu | 5,099.44 | 0.00 | 0.00 | 0.00 |
| Gansu | Maqu | 1,1458.28 | 0.00 | 0.00 | 0.00 |
| Gansu | Minqing | 20,183.61 | 0.00 | 0.00 | 0.00 |
| Gansu | Ming | 3,942.44 | 330.93 | 0.00 | 0.00 |
| Gansu | Ning | 0.00 | 0.00 | 2,326.70 | 854.00 |
| Gansu | Pingliang District | 0.00 | 1,088.44 | 1,224.71 | 0.00 |
| Gansu | Qinan | 0.00 | 786.29 | 1,159.54 | 0.00 |
| Gansu | Qingshui | 0.00 | 11.85 | 2,337.70 | 0.00 |
| Gansu | Qingyang | 0.00 | 2,344.47 | 893.78 | 0.00 |
| Gansu | Shandan | 6,279.29 | 0.00 | 0.00 | 0.00 |
| Gansu | Subei Mongolian Autonomous County | 73,720.49 | 0.00 | 0.00 | 0.00 |
| Gansu | Sunan Yugur Autonomous County | 26,618.63 | 0.00 | 0.00 | 0.00 |
| Gansu | Tianshui District | 0.00 | 1,676.68 | 4,879.38 | 400.34 |
| Gansu | Tianzhu Tibetan Autonomous County | 7,265.32 | 0.00 | 0.00 | 0.00 |
| Gansu | Tongwei | 1,188.32 | 2321.62 | 0.00 | 0.00 |
| Gansu | Weiyuan | 2,508.67 | 0.00 | 0.00 | 0.00 |
| Gansu | Wen | 682.18 | 4,370.70 | 829.45 | 0.00 |
| Gansu | Wudu | 287.77 | 3,642.82 | 1,621.66 | 0.00 |
| Gansu | Wushan | 266.61 | 2,130.34 | 0.00 | 0.00 |
| Gansu | Wuwei | 7,493.00 | 0.00 | 0.00 | 0.00 |
| Gansu | Xifeng | 0.00 | 0.85 | 1,177.31 | 0.00 |
| Gansu | Xihe | 6.77 | 1,966.99 | 239.53 | 0.00 |
| Gansu | Xiahe | 0.00 | 0.00 | 0.00 | 0.00 |
| Gansu | Yongchang | 0.00 | 0.00 | 0.00 | 0.00 |
| Gansu | Yongcheng | 0.00 | 0.00 | 0.00 | 0.00 |
| Gansu | Yongjing | 0.00 | 0.00 | 0.00 | 0.00 |
| Gansu | Yuzhong | 0.00 | 0.00 | 0.00 | 0.00 |
| Gansu | Yumen | 0.00 | 0.00 | 0.00 | 0.00 |
| Gansu | Zhangjiachuan Hui Autonomous County | 0.00 | 307.24 | 1,235.71 | 0.00 |
| Gansu | Zhangye | 4,686.40 | 0.00 | 0.00 | 0.00 |
| Gansu | Zhang | 2,595.00 | 27.93 | 0.00 | 0.00 |
| Gansu | Zhengyuan | 0.00 | 2,633.93 | 1,607.27 | 0.00 |
| Gansu | Zhengning | 0.00 | 0.00 | 270.00 | 1,344.90 |
| Gansu | Zhouqu | 2,850.61 | 724.50 | 0.85 | 0.00 |
| Gansu | Zhuanglang | 0.00 | 1,571.73 | 220.91 | 0.00 |
| Gansu | Zhuoni | 6,166.72 | 0.00 | 0.00 | 0.00 |
| Guangdong | Baoan District | 1,598.45 | 0.00 | 0.00 | 0.00 |
| Guangdong | Boluo | 3,150.61 | 0.00 | 0.00 | 0.00 |
| Guangdong | Chaoyang District | 975.57 | 0.00 | 0.00 | 0.00 |
| Guangdong | Chaozhou District | 1,436.84 | 0.00 | 0.00 | 0.00 |
| Guangdong | Chenghai District | 234.00 | 0.00 | 0.00 | 0.00 |
| Guangdong | Conghua District | 2,120.33 | 0.00 | 0.00 | 0.00 |
| Guangdong | Dapu | 2,643.88 | 0.00 | 0.00 | 0.00 |
| Guangdong | Deqing | 2,164.94 | 0.00 | 0.00 | 0.00 |
| Guangdong | Dianbai | 2,121.17 | 0.00 | 0.00 | 0.00 |
| Guangdong | Dongguan District | 2,530.25 | 0.00 | 0.00 | 0.00 |
| Guangdong | Doumen | 654.03 | 0.00 | 0.00 | 0.00 |
| Guangdong | Enping District | 1,787.00 | 0.00 | 0.00 | 0.00 |
| Guangdong | Panyu District | 971.36 | 0.00 | 0.00 | 0.00 |
| Guangdong | Fengshun | 2,907.35 | 0.00 | 0.00 | 0.00 |
| Guangdong | Fengkai | 2,919.97 | 0.00 | 0.00 | 0.00 |
| Guangdong | Fogang | 1,383.81 | 0.00 | 0.00 | 0.00 |
| Guangdong | Foshan District | 105.22 | 0.00 | 0.00 | 0.00 |
| Guangdong | Gaoming District | 1,000.82 | 0.00 | 0.00 | 0.00 |
| Guangdong | Gaozhou District | 3,437.64 | 0.00 | 0.00 | 0.00 |
| Guangdong | Guangning | 2,594.22 | 0.00 | 0.00 | 0.00 |
| Guangdong | Guangzhou District | 1,432.63 | 0.00 | 0.00 | 0.00 |
| Guangdong | Hiafeng | 1,744.91 | 0.00 | 0.00 | 0.00 |
| Guangdong | Heping | 2,469.65 | 0.00 | 0.00 | 0.00 |
| Guangdong | Heyuan District | 4,680.04 | 0.00 | 0.00 | 0.00 |
| Guangdong | Heshan District | 1,138.02 | 0.00 | 0.00 | 0.00 |
| Guangdong | Huadu District | 3,475.52 | 80.81 | 0.00 | 0.00 |
| Guangdong | Huazhou District | 2,504.16 | 0.00 | 0.00 | 0.00 |
| Guangdong | Huaiji | 3,878.71 | 0.00 | 0.00 | 0.00 |
| Guangdong | Huidong | 3,700.26 | 0.00 | 0.00 | 0.00 |
| Guangdong | Huilai | 829.11 | 0.00 | 0.00 | 0.00 |
| Guangdong | Huiyang | 2,350.12 | 0.00 | 0.00 | 0.00 |
| Guangdong | Huizhou District | 436.02 | 0.00 | 0.00 | 0.00 |
| Guangdong | Jiangmen District | 138.89 | 0.00 | 0.00 | 0.00 |
| Guangdong | Jiaoling | 1041.22 | 0.00 | 0.00 | 0.00 |
| Guangdong | Jiexi | 1,408.22 | 0.00 | 0.00 | 0.00 |
| Guangdong | Jieyang District | 1,032.81 | 0.00 | 0.00 | 0.00 |
| Guangdong | Kaiping District | 1,755.01 | 0.00 | 0.00 | 0.00 |
| Guangdong | Lechang District | 2,047.94 | 578.27 | 0.00 | 0.00 |
| Guangdong | Huozhou District | 3,547.06 | 0.00 | 0.00 | 0.00 |
| Guangdong | Liannan Yao Autonomous County | 1,264.28 | 0.00 | 0.00 | 0.00 |
| Guangdong | Lianping | 2,442.71 | 0.00 | 0.00 | 0.00 |
| Guangdong | Lianshan Zhuang and Yao Autonomous County | 1,300.48 | 0.00 | 0.00 | 0.00 |
| Guangdong | Lianzhou | 2,857.69 | 0.00 | 0.00 | 0.00 |
| Guangdong | Lianjiang District | 2,857.69 | 0.00 | 0.00 | 0.00 |
| Guangdong | Longchuan | 3,333.26 | 0.00 | 0.00 | 0.00 |
| Guangdong | Longmen | 2,464.60 | 0.00 | 0.00 | 0.00 |
| Guangdong | Lufeng District | 748.30 | 0.00 | 0.00 | 0.00 |
| Guangdong | Luhe | 1,018.50 | 0.00 | 0.00 | 0.00 |
| Guangdong | Luoding District | 2,484.80 | 0.00 | 0.00 | 0.00 |
| Guangdong | Maoming District | 535.34 | 0.00 | 0.00 | 0.00 |
| Guangdong | Mei | 2,941.86 | 0.00 | 0.00 | 0.00 |
| Guangdong | Mei District | 323.23 | 0.00 | 0.00 | 0.00 |
| Guangdong | Nanhai District | 1,214.62 | 0.00 | 0.00 | 0.00 |
| Guangdong | Pingyuan | 1,533.64 | 0.00 | 0.00 | 0.00 |
| Guangdong | Puning District | 1,751.65 | 0.00 | 0.00 | 0.00 |
| Guangdong | Qingyuan District | 3,917.43 | 0.00 | 0.00 | 0.00 |
| Guangdong | Raoping | 1,723.03 | 0.00 | 0.00 | 0.00 |
| Guangdong | Renhua | 1,693.57 | 13.47 | 0.00 | 0.00 |
| Guangdong | Ruyuan Yao Autonomous County | 2,435.98 | 46.30 | 0.00 | 0.00 |
| Guangdong | Sanshui District | 952.84 | 0.00 | 0.00 | 0.00 |
| Guangdong | Shaoguan District | 351.84 | 0.00 | 0.00 | 0.00 |
| Guangdong | Shenzhen District | 308.92 | 0.00 | 0.00 | 0.00 |
| Guangdong | Shixing | 2,297.93 | 4.21 | 0.00 | 0.00 |
| Guangdong | Shunde District | 867.83 | 0.00 | 0.00 | 0.00 |
| Guangdong | Sihui District | 1,306.37 | 0.00 | 0.00 | 0.00 |
| Guangdong | Jixi | 2,046.25 | 0.00 | 0.00 | 0.00 |
| Guangdong | Taishan District | 3,172.49 | 0.00 | 0.00 | 0.00 |
| Guangdong | Wengyuan | 2,348.44 | 0.00 | 0.00 | 0.00 |
| Guangdong | Wuchuan District | 864.46 | 0.00 | 0.00 | 0.00 |
| Guangdong | Wuhua | 3,422.49 | 0.00 | 0.00 | 0.00 |
| Guangdong | Xinfeng | 2,028.58 | 0.00 | 0.00 | 0.00 |
| Guangdong | Xinhui District | 1,685.15 | 0.00 | 0.00 | 0.00 |
| Guangdong | Xinxing | 1,633.80 | 0.00 | 0.00 | 0.00 |
| Guangdong | Xingyi District | 3,307.17 | 0.00 | 0.00 | 0.00 |
| Guangdong | XingningDistrict | 2,211.23 | 0.00 | 0.00 | 0.00 |
| Guangdong | Xuwen | 1,574.88 | 0.00 | 0.00 | 0.00 |
| Guangdong | Yangchuan District | 4,266.75 | 0.00 | 0.00 | 0.00 |
| Guangdong | Yangjiang District | 2,521.83 | 0.00 | 0.00 | 0.00 |
| Guangdong | Yangshan | 3,659.86 | 0.00 | 0.00 | 0.00 |
| Guangdong | Yangxi | 1,388.02 | 0.00 | 0.00 | 0.00 |
| Guangdong | Yizhang | 2,345.07 | 1.68 | 0.00 | 0.00 |
| Guangdong | Yingde | 6,065.53 | 0.00 | 0.00 | 0.00 |
| Guangdong | Yunan | 2,075.71 | 0.00 | 0.00 | 0.00 |
| Guangdong | Yunfu District | 2,106.86 | 0.00 | 0.00 | 0.00 |
| Guangdong | Zengcheng District | 1,845.92 | 0.00 | 0.00 | 0.00 |
| Guangdong | Zhanjiang District | 1,197.79 | 0.00 | 0.00 | 0.00 |
| Guangdong | Zhaoqing District | 143.09 | 0.00 | 0.00 | 0.00 |
| Guangdong | Zhongshan District | 1,643.91 | 0.00 | 0.00 | 0.00 |
| Guangdong | Zhuhai District | 236.53 | 0.00 | 0.00 | 0.00 |
| Guangdong | Zijin | 3,894.70 | 0.00 | 0.00 | 0.00 |
| Guangxi | Quanzhou | 3,294.64 | 787.21 | 0.00 | 0.00 |
| Guangxi | Ziyuan | 1,136.99 | 854.93 | 0.00 | 0.00 |
| Guangxi | Bama Yao Autonomous County | 1,948.90 | 0.00 | 0.00 | 0.00 |
| Guangxi | Baise District | 3,818.91 | 0.00 | 0.00 | 0.00 |
| Guangxi | Beihai District | 112.34 | 0.00 | 0.00 | 0.00 |
| Guangxi | Beiliu District | 2,482.73 | 0.00 | 0.00 | 0.00 |
| Guangxi | Binyang | 2,341.70 | 0.00 | 0.00 | 0.00 |
| Guangxi | Boyang | 3,846.00 | 0.00 | 0.00 | 0.00 |
| Guangxi | Cangwu | 4,312.91 | 0.00 | 0.00 | 0.00 |
| Guangxi | Cenxi | 2,797.45 | 0.00 | 0.00 | 0.00 |
| Guangxi | Chongzuo | 2,952.03 | 0.00 | 0.00 | 0.00 |
| Guangxi | Dahua Yao Autonomous County | 2,943.26 | 0.00 | 0.00 | 0.00 |
| Guangxi | Daxin | 2,779.93 | 0.00 | 0.00 | 0.00 |
| Guangxi | Debao | 2,600.65 | 0.00 | 0.00 | 0.00 |
| Guangxi | Donglan | 2,436.52 | 0.00 | 0.00 | 0.00 |
| Guangxi | Dongxin District | 420.69 | 0.00 | 0.00 | 0.00 |
| Guangxi | Du'an Yao Autonomous County | 4,110.53 | 0.00 | 0.00 | 0.00 |
| Guangxi | Fangchenggang District | 2,545.68 | 0.00 | 0.00 | 0.00 |
| Guangxi | Fengshan | 1,762.45 | 0.00 | 0.00 | 0.00 |
| Guangxi | Fusui | 2,860.40 | 0.00 | 0.00 | 0.00 |
| Guangxi | Fuchuan Yao Autonomous County | 1,557.68 | 0.00 | 0.00 | 0.00 |
| Guangxi | Gongcheng Yao Autonomous County | 2,172.79 | 0.00 | 0.00 | 0.00 |
| Guangxi | Guanyang | 1,901.89 | 0.00 | 0.00 | 0.00 |
| Guangxi | Guigang District | 3,566.34 | 0.00 | 0.00 | 0.00 |
| Guangxi | Guilin District | 584.03 | 0.00 | 0.00 | 0.00 |
| Guangxi | Guiping District | 4,148.77 | 0.00 | 0.00 | 0.00 |
| Guangxi | Hepu | 2,874.74 | 0.00 | 0.00 | 0.00 |
| Guangxi | Heshan District | 365.72 | 0.00 | 0.00 | 0.00 |
| Guangxi | Hechi District | 2,376.76 | 0.00 | 0.00 | 0.00 |
| Guangxi | Hezhou | 5,194.13 | 0.00 | 0.00 | 0.00 |
| Guangxi | Heng | 3,468.33 | 0.00 | 0.00 | 0.00 |
| Guangxi | Huanjiang Maonan Autonomous County | 4,661.89 | 0.00 | 0.00 | 0.00 |
| Guangxi | Jinxiu Yao Autonomous County | 2,517.79 | 0.00 | 0.00 | 0.00 |
| Guangxi | Jingxi | 3,363.16 | 0.00 | 0.00 | 0.00 |
| Guangxi | Laibin | 4,436.41 | 0.00 | 0.00 | 0.00 |
| Guangxi | Leye | 2,688.30 | 0.00 | 0.00 | 0.00 |
| Guangxi | Lipu | 1,769.62 | 0.00 | 0.00 | 0.00 |
| Guangxi | Lingui | 2,281.15 | 0.00 | 0.00 | 0.00 |
| Guangxi | Linchuan | 2,364.01 | 0.00 | 0.00 | 0.00 |
| Guangxi | Lingshan | 3,555.98 | 0.00 | 0.00 | 0.00 |
| Guangxi | Lingyun | 2,070.00 | 0.00 | 0.00 | 0.00 |
| Guangxi | Liucheng | 2,225.37 | 0.00 | 0.00 | 0.00 |
| Guangxi | Liujiang | 2,568.78 | 0.00 | 0.00 | 0.00 |
| Guangxi | Liuzhou District | 580.05 | 0.00 | 0.00 | 0.00 |
| Guangxi | Longsheng ethnic autonomous counties | 2,530.54 | 19.92 | 0.00 | 0.00 |
| Guangxi | Longzhou | 2,338.52 | 0.00 | 0.00 | 0.00 |
| Guangxi | Longan | 2,307.44 | 0.00 | 0.00 | 0.00 |
| Guangxi | Longlin ethnic autonomous counties | 3,650.00 | 0.00 | 0.00 | 0.00 |
| Guangxi | Luchuan | 1,561.67 | 0.00 | 0.00 | 0.00 |
| Guangxi | Luzhai | 3,438.06 | 0.00 | 0.00 | 0.00 |
| Guangxi | Luocheng Mulao Autonomous County | 2,693.08 | 0.00 | 0.00 | 0.00 |
| Guangxi | Mashan | 2,376.76 | 0.00 | 0.00 | 0.00 |
| Guangxi | Mengshan | 1,301.92 | 0.00 | 0.00 | 0.00 |
| Guangxi | Napo | 2,272.38 | 0.00 | 0.00 | 0.00 |
| Guangxi | Nandan | 4,053.96 | 0.00 | 0.00 | 0.00 |
| Guangxi | Nanning District | 1,762.45 | 0.00 | 0.00 | 0.00 |
| Guangxi | Ningming | 3,764.73 | 0.00 | 0.00 | 0.00 |
| Guangxi | Pingguo | 2,506.63 | 0.00 | 0.00 | 0.00 |
| Guangxi | Pingle | 1,979.97 | 0.00 | 0.00 | 0.00 |
| Guangxi | Pingnan | 3,039.67 | 0.00 | 0.00 | 0.00 |
| Guangxi | Pinxiang District | 684.42 | 0.00 | 0.00 | 0.00 |
| Guangxi | Pubei | 2,556.83 | 0.00 | 0.00 | 0.00 |
| Guangxi | Qinzhou District | 4,491.38 | 0.00 | 0.00 | 0.00 |
| Guangxi | Rong | 2,278.76 | 0.00 | 0.00 | 0.00 |
| Guangxi | Rongan | 2,971.95 | 0.00 | 0.00 | 0.00 |
| Guangxi | Rongshui Miao Autonomous County | 4,776.63 | 14.34 | 0.00 | 0.00 |
| Guangxi | Sanjiang Dong Autonomous County | 2,467.59 | 0.00 | 0.00 | 0.00 |
| Guangxi | Shanglin | 1,914.63 | 0.00 | 0.00 | 0.00 |
| Guangxi | Shangsi | 2,826.14 | 0.00 | 0.00 | 0.00 |
| Guangxi | Teng | 3,998.98 | 0.00 | 0.00 | 0.00 |
| Guangxi | Tiandeng | 2,181.55 | 0.00 | 0.00 | 0.00 |
| Guangxi | Tiane | 3,269.94 | 0.00 | 0.00 | 0.00 |
| Guangxi | Tiandong | 2,869.16 | 0.00 | 0.00 | 0.00 |
| Guangxi | Tianlin | 5,616.42 | 0.00 | 0.00 | 0.00 |
| Guangxi | Tianyang | 2,442.10 | 0.00 | 0.00 | 0.00 |
| Guangxi | Wuzhou District | 313.13 | 0.00 | 0.00 | 0.00 |
| Guangxi | Wuming | 3,426.10 | 0.00 | 0.00 | 0.00 |
| Guangxi | Wuxuan | 1,724.21 | 0.00 | 0.00 | 0.00 |
| Guangxi | Xiangzhou | 1,944.91 | 0.00 | 0.00 | 0.00 |
| Guangxi | Yicheng | 2,549.66 | 0.00 | 0.00 | 0.00 |
| Guangxi | Xingan | 2,397.48 | 18.33 | 0.00 | 0.00 |
| Guangxi | Yangshuo | 1,478.01 | 0.00 | 0.00 | 0.00 |
| Guangxi | Yizhou District | 3,928.87 | 0.00 | 0.00 | 0.00 |
| Guangxi | Yongning | 4,731.21 | 0.00 | 0.00 | 0.00 |
| Guangxi | Yongfu | 2,864.38 | 0.00 | 0.00 | 0.00 |
| Guangxi | Yulin District | 2,775.94 | 0.00 | 0.00 | 0.00 |
| Guangxi | Zhaoping | 3,252.41 | 0.00 | 0.00 | 0.00 |
| Guangxi | Zhongshan | 1,896.31 | 0.00 | 0.00 | 0.00 |
| Guizhou | Anlong | 2,280.33 | 0.00 | 0.00 | 0.00 |
| Guizhou | Anshun District | 1,746.52 | 0.00 | 0.00 | 0.00 |
| Guizhou | Bijie District | 1,242.67 | 2,269.29 | 0.00 | 0.00 |
| Guizhou | Ceheng | 2,621.74 | 0.00 | 0.00 | 0.00 |
| Guizhou | Cengkong | 1,196.15 | 337.48 | 0.00 | 0.00 |
| Guizhou | ChishuiDistrict | 269.67 | 1,272.63 | 335.11 | 0.00 |
| Guizhou | Congjiang | 3,042.80 | 327.23 | 0.00 | 0.00 |
| Guizhou | Dafang | 224.72 | 3,452.03 | 0.00 | 0.00 |
| Guizhou | Danzhai | 944.62 | 0.00 | 0.00 | 0.00 |
| Guizhou | Daozhen Gelao Miao Autonomous County | 32.33 | 2,215.67 | 3.94 | 0.00 |
| Guizhou | Dejiang | 26.02 | 2,116.32 | 0.00 | 0.00 |
| Guizhou | Duyun District | 2,346.56 | 0.00 | 0.00 | 0.00 |
| Guizhou | Dushan | 2,454.58 | 0.00 | 0.00 | 0.00 |
| Guizhou | Fenggang | 1,219.01 | 743.55 | 0.00 | 0.00 |
| Guizhou | Fuquan District | 1,735.48 | 0.00 | 0.00 | 0.00 |
| Guizhou | Guanling Buyi and Miao Autonomous County | 1,502.87 | 0.00 | 0.00 | 0.00 |
| Guizhou | Guiding | 1,675.55 | 0.00 | 0.00 | 0.00 |
| Guizhou | Guiyang District | 2,359.96 | 84.37 | 0.00 | 0.00 |
| Guizhou | Hezhang | 23.65 | 3,318.77 | 0.00 | 0.00 |
| Guizhou | Huangping | 1,702.36 | 0.00 | 0.00 | 0.00 |
| Guizhou | Huishui | 2,523.18 | 3.15 | 0.00 | 0.00 |
| Guizhou | Jianhe | 1,901.06 | 186.08 | 0.00 | 0.00 |
| Guizhou | Jiangkou | 1,170.91 | 771.15 | 0.00 | 0.00 |
| Guizhou | Jinsha | 2,570.49 | 32.33 | 0.00 | 0.00 |
| Guizhou | Jinping | 1,064.47 | 574.02 | 0.00 | 0.00 |
| Guizhou | Kaiyang | 2,097.40 | 0.00 | 0.00 | 0.00 |
| Guizhou | Kaili District | 1,318.36 | 0.00 | 0.00 | 0.00 |
| Guizhou | Leishan | 1,233.99 | 0.00 | 0.00 | 0.00 |
| Guizhou | Liping | 4,581.94 | 0.00 | 0.00 | 0.00 |
| Guizhou | Lipo | 2,415.16 | 24.44 | 0.00 | 0.00 |
| Guizhou | Liuzhi District | 1,174.07 | 654.45 | 0.00 | 0.00 |
| Guizhou | Longli | 1,500.50 | 70.96 | 0.00 | 0.00 |
| Guizhou | Luodian | 3,023.88 | 0.00 | 0.00 | 0.00 |
| Guizhou | Majiang | 1,271.05 | 0.00 | 0.00 | 0.00 |
| Guizhou | Meitan | 1,514.70 | 397.40 | 0.00 | 0.00 |
| Guizhou | nayong | 0.00 | 2,484.55 | 5.52 | 0.00 |
| Guizhou | Panxian District | 4,151.42 | 0.00 | 0.00 | 0.00 |
| Guizhou | Pingba | 1,034.50 | 0.00 | 0.00 | 0.00 |
| Guizhou | Pingtang | 2,858.29 | 0.00 | 0.00 | 0.00 |
| Guizhou | Puan | 1,470.54 | 0.00 | 0.00 | 0.00 |
| Guizhou | Puding | 754.59 | 383.21 | 0.00 | 0.00 |
| Guizhou | Qianxi | 1,402.73 | 1,193.78 | 0.00 | 0.00 |
| Guizhou | Qingzheng District | 1,548.60 | 0.00 | 0.00 | 0.00 |
| Guizhou | Qinglong | 1,325.46 | 0.00 | 0.00 | 0.00 |
| Guizhou | Renhuai District | 1,603.01 | 258.63 | 0.00 | 0.00 |
| Guizhou | Rongjiang | 3,174.48 | 162.43 | 0.00 | 0.00 |
| Guizhou | Sandushui autonomous county | 2,418.31 | 14.19 | 0.00 | 0.00 |
| Guizhou | Sanhui | 953.29 | 110.39 | 0.00 | 0.00 |
| Guizhou | Shibing | 1,204.03 | 391.88 | 0.00 | 0.00 |
| Guizhou | Shiqian | 1,137.80 | 1,085.76 | 0.00 | 0.00 |
| Guizhou | Shuicheng | 1,410.62 | 2,385.98 | 0.00 | 0.00 |
| Guizhou | Sinan | 1,387.75 | 893.36 | 0.00 | 0.00 |
| Guizhou | Songtao Miao Autonomous County | 2,489.28 | 486.50 | 0.00 | 0.00 |
| Guizhou | Suiyang | 275.18 | 2,393.08 | 0.00 | 0.00 |
| Guizhou | Taijiang | 1,248.98 | 0.00 | 0.00 | 0.00 |
| Guizhou | Tianzhu | 407.65 | 1,873.46 | 0.00 | 0.00 |
| Guizhou | Tongzi | 316.97 | 3,021.51 | 0.00 | 0.00 |
| Guizhou | Tongren | 1,461.87 | 123.01 | 0.00 | 0.00 |
| Guizhou | Wanshan District | 219.99 | 145.08 | 0.00 | 0.00 |
| Guizhou | Wangmo | 3,080.65 | 0.00 | 0.00 | 0.00 |
| Guizhou | Wei Ning Yi Hui and Miao Autonomous County | 2,100.55 | 4,412.41 | 0.00 | 0.00 |
| Guizhou | Wengning | 2,041.41 | 0.00 | 0.00 | 0.00 |
| Guizhou | Wulong Gelao and Miao Autonomous County | 33.12 | 2,943.45 | 3.15 | 0.00 |
| Guizhou | Xifeng | 1059.74 | 0.00 | 0.00 | 0.00 |
| Guizhou | Xishui | 402.13 | 2,834.64 | 26.02 | 0.00 |
| Guizhou | Xingren | 1,826.94 | 0.00 | 0.00 | 0.00 |
| Guizhou | Xingyi District | 2,960.01 | 0.00 | 0.00 | 0.00 |
| Guizhou | Xiuwen | 1,138.59 | 0.00 | 0.00 | 0.00 |
| Guizhou | Yanhe Tujia Autonomous County | 574.02 | 1,993.31 | 0.00 | 0.00 |
| Guizhou | Yinjiang Tujia and Miao Autonomous County | 156.12 | 1,890.81 | 0.00 | 0.00 |
| Guizhou | Yuqing | 1,683.44 | 7.10 | 0.00 | 0.00 |
| Guizhou | Yuping Dong Autonomous County | 521.19 | 26.02 | 0.00 | 0.00 |
| Guizhou | changshun | 1,564.37 | 0.00 | 0.00 | 0.00 |
| Guizhou | Zhenfeng | 1,532.04 | 0.00 | 0.00 | 0.00 |
| Guizhou | Zhenning Buyi and Miao Autonomous County | 1,754.40 | 0.00 | 0.00 | 0.00 |
| Guizhou | Zhengyuan | 1,263.96 | 674.95 | 0.00 | 0.00 |
| Guizhou | Zhengan | 7.10 | 2,713.21 | 0.00 | 0.00 |
| Guizhou | Zhijin | 234.18 | 2,656.44 | 43.37 | 0.00 |
| Guizhou | Zhongshan District | 1.58 | 446.29 | 0.00 | 0.00 |
| Guizhou | Ziyun Miao and Buyi Autonomous County | 2,364.69 | 0.00 | 0.00 | 0.00 |
| Guizhou | Zunyi District | 259.41 | 69.39 | 0.00 | 0.00 |
| Hainan | Baisha Li Autonomous County | 2,419.53 | 0.00 | 0.00 | 0.00 |
| Hainan | Baoting Li and Miao Autonomous County | 1,057.39 | 0.00 | 0.00 | 0.00 |
| Hainan | Changjiang Li Autonomous County | 1,820.19 | 0.00 | 0.00 | 0.00 |
| Hainan | Chengmai | 2,335.50 | 0.00 | 0.00 | 0.00 |
| Hainan | Danzhou District | 3,672.70 | 0.00 | 0.00 | 0.00 |
| Hainan | Dingan | 1,344.60 | 0.00 | 0.00 | 0.00 |
| Hainan | Dongfang District | 2,560.83 | 0.00 | 0.00 | 0.00 |
| Hainan | Haikou District | 204.09 | 0.00 | 0.00 | 0.00 |
| Hainan | Ledong Li Autonomous County | 3,029.04 | 0.00 | 0.00 | 0.00 |
| Hainan | Lingao | 1,436.02 | 0.00 | 0.00 | 0.00 |
| Hainan | Lingshui Li Autonomous County | 867.15 | 0.00 | 0.00 | 0.00 |
| Hainan | Qionghai City | 3,586.82 | 0.00 | 0.00 | 0.00 |
| Hainan | Qiongzhong Li and Miao Autonomous County | 3,529.56 | 0.00 | 0.00 | 0.00 |
| Hainan | Sanya District | 1,329.82 | 0.00 | 0.00 | 0.00 |
| Hainan | Tongshen District | 779.42 | 0.00 | 0.00 | 0.00 |
| Hainan | Tunchang | 1,409.24 | 0.00 | 0.00 | 0.00 |
| Hainan | Wanning District | 1,962.41 | 0.00 | 0.00 | 0.00 |
| Hainan | Wenchang District | 2,055.68 | 0.00 | 0.00 | 0.00 |
| Hebei | Anguo District | 0.00 | 483.92 | 0.00 | 0.00 |
| Hebei | Anping | 0.00 | 508.87 | 0.00 | 0.00 |
| Hebei | Anxin | 0.00 | 778.56 | 0.00 | 0.00 |
| Hebei | Bazhou District | 0.00 | 877.70 | 0.00 | 0.00 |
| Hebei | Baixing | 0.00 | 2.77 | 248.89 | 0.00 |
| Hebei | Baoding District | 0.00 | 137.27 | 0.00 | 0.00 |
| Hebei | Botou District | 0.00 | 1,035.08 | 0.00 | 0.00 |
| Hebei | Boye | 0.00 | 370.22 | 0.00 | 0.00 |
| Hebei | Cang | 0.00 | 1,620.22 | 0.00 | 0.00 |
| Hebei | Cangzhou District | 0.00 | 172.63 | 0.00 | 0.00 |
| Hebei | Changli | 9.01 | 1,073.21 | 34.66 | 0.00 |
| Hebei | Chengan | 0.00 | 0.00 | 92.21 | 405.57 |
| Hebei | Chengde District | 54.77 | 651.69 | 0.00 | 0.00 |
| Hebei | Chengde | 1,144.62 | 3,195.37 | 0.00 | 0.00 |
| Hebei | Chicheng | 2,981.84 | 2,642.82 | 0.00 | 0.00 |
| Hebei | Chongli | 2,294.09 | 226.71 | 0.00 | 0.00 |
| Hebei | Dachang Hui Autonomous County | 0.00 | 182.34 | 0.00 | 0.00 |
| Hebei | Dacheng | 0.00 | 892.96 | 0.00 | 0.00 |
| Hebei | Daming | 0.00 | 0.00 | 603.16 | 461.04 |
| Hebei | Dingxing | 0.00 | 750.83 | 0.00 | 0.00 |
| Hebei | Dingzhou District | 0.00 | 1,225.04 | 80.42 | 0.00 |
| Hebei | Dongguang | 0.00 | 714.09 | 0.00 | 0.00 |
| Hebei | Feixiang | 0.00 | 0.00 | 400.03 | 95.67 |
| Hebei | Fenfnan District | 0.00 | 1,340.13 | 0.00 | 0.00 |
| Hebei | Fengning Manchu Autonomous County | 0.00 | 9,357.33 | 51.30 | 0.00 |
| Hebei | Fengrun | 0.00 | 0.69 | 1321.41 | 8.32 |
| Hebei | Funing | 61.01 | 1,661.12 | 6.24 | 0.00 |
| Hebei | Fucheng | 0.00 | 711.31 | 0.00 | 0.00 |
| Hebei | Fuping | 1.39 | 1,331.81 | 1279.81 | 0.69 |
| Hebei | Gaobeidian District | 0.00 | 700.22 | 0.00 | 0.00 |
| Hebei | Gaoyang | 0.00 | 498.47 | 0.00 | 0.00 |
| Hebei | Gaoyi | 0.00 | 0.00 | 198.28 | 1.39 |
| Hebei | Gaocheng District | 0.00 | 730.73 | 95.67 | 0.00 |
| Hebei | Guyuan | 0.00 | 3,867.17 | 0.00 | 0.00 |
| Hebei | Guan | 22.88 | 700.92 | 0.00 | 0.00 |
| Hebei | Gucheng | 0.00 | 153.91 | 812.54 | 0.00 |
| Hebei | Guantao | 0.00 | 0.00 | 420.13 | 22.88 |
| Hebei | Guangping | 0.00 | 0.00 | 266.92 | 84.58 |
| Hebei | Guangzong | 0.00 | 0.00 | 508.87 | 0.00 |
| Hebei | Haixing | 0.00 | 814.61 | 0.00 | 0.00 |
| Hebei | Handan District | 0.00 | 0.00 | 2.77 | 469.36 |
| Hebei | Xingtang | 0.00 | 0.69 | 999.03 | 0.00 |
| Hebei | Hejian District | 0.00 | 1,361.62 | 0.00 | 0.00 |
| Hebei | Hengshui District | 0.00 | 586.52 | 18.72 | 0.00 |
| Hebei | Huaian | 180.95 | 1,607.74 | 0.00 | 0.00 |
| Hebei | Huailai | 25.65 | 1,865.64 | 2.08 | 0.00 |
| Hebei | Huangye District | 0.00 | 2,087.49 | 0.00 | 0.00 |
| Hebei | Jize | 0.00 | 0.00 | 345.26 | 1.39 |
| Hebei | Jizhou District | 0.00 | 603.16 | 330.01 | 0.00 |
| Hebei | Jinzhou District | 0.00 | 632.97 | 0.00 | 0.00 |
| Hebei | Jing | 0.00 | 1218.11 | 0.69 | 0.00 |
| Hebei | Julu | 0.00 | 0.00 | 607.32 | 0.00 |
| Hebei | Kangbao | 3616.20 | 0.00 | 0.00 | 0.00 |
| Hebei | Kuancheng Manchu Autonomous County | 3.47 | 2,053.52 | 0.00 | 0.00 |
| Hebei | Laishui | 0.00 | 1,486.41 | 232.25 | 0.00 |
| Hebei | Laiyuan | 0.69 | 2,068.78 | 481.84 | 0.00 |
| Hebei | Langfang District | 0.00 | 1,027.45 | 0.00 | 0.00 |
| Hebei | Leting | 0.00 | 951.19 | 0.00 | 0.00 |
| Hebei | Li | 0.00 | 680.12 | 119.94 | 655.16 |
| Hebei | Linxi | 0.00 | 0.00 | 556.71 | 0.00 |
| Hebei | Linzhang | 0.00 | 0.00 | 345.26 | 389.63 |
| Hebei | Lingshou | 0.00 | 172.63 | 921.38 | 0.00 |
| Hebei | Longhua | 5205.22 | 659.32 | 0.00 | 0.00 |
| Hebei | Longyao | 0.00 | 52.69 | 740.43 | 4.85 |
| Hebei | Lulong | 0.00 | 957.43 | 47.84 | 0.00 |
| Hebei | Luquan | 0.00 | 0.00 | 644.07 | 4.16 |
| Hebei | Luancheng | 0.00 | 72.10 | 333.47 | 0.00 |
| Hebei | Luannan | 0.00 | 1,513.45 | 0.00 | 0.00 |
| Hebei | Luanping | 922.77 | 2,521.49 | 0.00 | 0.00 |
| Hebei | Luan | 0.00 | 1,028.15 | 23.57 | 0.00 |
| Hebei | Mancheng | 0.00 | 508.18 | 248.89 | 0.00 |
| Hebei | Mengcun Hui Autonomous County | 0.00 | 407.65 | 0.00 | 0.00 |
| Hebei | Nangong District | 0.00 | 1.39 | 867.30 | 0.00 |
| Hebei | Nanhe | 0.00 | 0.00 | 386.16 | 27.04 |
| Hebei | Nanpi | 0.00 | 847.89 | 85.27 | 703.69 |
| Hebei | Ningjin | 0.00 | 899.20 | 244.73 | 0.00 |
| Hebei | Pingquan | 574.04 | 2,928.45 | 0.00 | 0.00 |
| Hebei | Pingshan | 0.00 | 1,665.97 | 1,032.31 | 0.00 |
| Hebei | Pingxiang | 0.00 | 422.21 | 0.00 | 0.00 |
| Hebei | Qianan District | 0.69 | 1,292.29 | 0.00 | 0.00 |
| Hebei | Qianxi | 0.69 | 1,487.80 | 0.00 | 0.00 |
| Hebei | Qinhuangdao District | 56.16 | 1,64.31 | 0.00 | 0.00 |
| Hebei | Qinglong Manchu Autonomous County | 2.77 | 3,734.75 | 0.00 | 0.00 |
| Hebei | Qing | 0.00 | 1,049.64 | 0.00 | 0.00 |
| Hebei | Qinghe | 0.00 | 0.00 | 516.50 | 0.00 |
| Hebei | Qingyuan | 0.00 | 971.30 | 0.00 | 0.00 |
| Hebei | Qiuyang | 0.00 | 0.00 | 462.42 | 0.00 |
| Hebei | Quyang | 0.00 | 469.36 | 638.52 | 0.00 |
| Hebei | Quzhou | 0.00 | 0.00 | 592.76 | 63.78 |
| Hebei | Raoyang | 0.00 | 575.43 | 0.00 | 0.00 |
| Hebei | Renqiu District | 0.00 | 1053.11 | 0.00 | 0.00 |
| Hebei | Ren | 0.00 | 0.00 | 463.81 | 0.69 |
| Hebei | Rongcheng | 0.00 | 316.14 | 0.00 | 0.00 |
| Hebei | SanheDistrict | 0.69 | 617.72 | 32.58 | 0.00 |
| Hebei | Shahe District | 0.00 | 0.00 | 210.76 | 785.50 |
| Hebei | Shangyi | 2681.64 | 159.46 | 0.00 | 0.00 |
| Hebei | She | 0.00 | 0.00 | 1,085.00 | 424.99 |
| Hebei | Shenze | 0.00 | 324.46 | 0.00 | 0.00 |
| Hebei | Shenzhou | 0.00 | 1,275.65 | 0.00 | 0.00 |
| Hebei | Shijiazhuang District | 0.00 | 0.00 | 343.18 | 0.00 |
| Hebei | Shunping | 0.00 | 329.31 | 416.67 | 0.00 |
| Hebei | Suning | 0.00 | 547.70 | 0.00 | 0.00 |
| Hebei | Tanghai | 0.00 | 722.41 | 0.00 | 0.00 |
| Hebei | Tangshan District | 0.00 | 753.61 | 40.90 | 0.00 |
| Hebei | Tang | 0.00 | 820.16 | 643.37 | 0.00 |
| Hebei | Wanquan | 272.46 | 918.61 | 0.00 | 0.00 |
| Hebei | Wangdu | 0.00 | 396.56 | 0.00 | 0.00 |
| Hebei | Wei | 0.00 | 0.00 | 1,035.77 | 0.00 |
| Hebei | Weichang Mongolian Autonomous County | 9,732.39 | 2.08 | 0.00 | 0.00 |
| Hebei | Wei | 315.45 | 3,040.77 | 0.00 | 0.00 |
| Hebei | Weii | 0.00 | 0.00 | 723.79 | 125.49 |
| Hebei | Wenan | 0.00 | 1,055.88 | 0.00 | 0.00 |
| Hebei | Wuji | 0.00 | 512.34 | 0.00 | 0.00 |
| Hebei | Wuqiao | 0.00 | 615.64 | 0.00 | 0.00 |
| Hebei | Wuan District | 0.00 | 0.00 | 547.70 | 1,311.70 |
| Hebei | Wuqiang | 0.00 | 479.06 | 0.00 | 0.00 |
| Hebei | Wuyi | 0.00 | 818.77 | 0.00 | 0.00 |
| Hebei | Xian | 0.00 | 1,210.48 | 0.00 | 0.00 |
| Hebei | Xianghe | 0.00 | 470.74 | 0.00 | 0.00 |
| Hebei | Xinji District | 0.00 | 971.30 | 0.00 | 0.00 |
| Hebei | Xinhe | 0.00 | 90.13 | 291.18 | 0.00 |
| Hebei | Xinle District | 0.00 | 196.89 | 340.41 | 0.00 |
| Hebei | Xintai District | 0.00 | 0.00 | 0.00 | 127.57 |
| Hebei | Xingtai | 0.00 | 0.00 | 1,044.79 | 953.97 |
| Hebei | Xiong | 0.00 | 544.93 | 0.00 | 0.00 |
| Hebei | Xushui | 0.00 | 753.61 | 22.88 | 0.00 |
| Hebei | Xuanhua | 253.05 | 2219.91 | 0.00 | 0.00 |
| Hebei | Yanshan | 0.00 | 816.69 | 0.00 | 0.00 |
| Hebei | Yangyuan | 112.31 | 1,817.80 | 0.00 | 0.00 |
| Hebei | YI | 0.00 | 1,577.93 | 1,159.87 | 0.00 |
| Hebei | Yongnian | 0.00 | 0.00 | 293.95 | 591.38 |
| Hebei | Yongqing | 0.00 | 802.83 | 0.00 | 0.00 |
| Hebei | Yutian | 332.78 | 870.08 | 0.00 | 0.00 |
| Hebei | Yuanshi | 0.00 | 0.00 | 680.81 | 21.49 |
| Hebei | Zanhuang | 0.00 | 0.00 | 384.78 | 502.63 |
| Hebei | Zaoqiang | 0.00 | 259.29 | 665.56 | 0.00 |
| Hebei | Zhangbei | 0.00 | 4,482.12 | 0.00 | 0.00 |
| Hebei | Zhangjiakou District | 57.54 | 587.22 | 0.00 | 0.00 |
| Hebei | Zhao | 0.00 | 327.93 | 359.12 | 0.00 |
| Hebei | Zhengding | 0.00 | 6.24 | 587.91 | 0.00 |
| Hebei | Zhuolu | 70.02 | 2,798.12 | 48.53 | 0.00 |
| Hebei | Zhuozhou | 0.00 | 760.54 | 0.00 | 0.00 |
| Hebei | Zunhua District | 0.00 | 1,527.32 | 0.00 | 0.00 |
| Heilongjiang | Dongning | 6,921.70 | 1.16 | 0.00 | 0.00 |
| Heilongjiang | Acheng District | 2,653.19 | 0.00 | 0.00 | 0.00 |
| Heilongjiang | Anda District | 3,557.86 | 0.00 | 0.00 | 0.00 |
| Heilongjiang | Bayan | 3,047.61 | 0.00 | 0.00 | 0.00 |
| Heilongjiang | Baiquan | 3,583.92 | 0.00 | 0.00 | 0.00 |
| Heilongjiang | Baoqing | 9,735.90 | 0.00 | 0.00 | 0.00 |
| Heilongjiang | Beian District | 7,185.22 | 0.00 | 0.00 | 0.00 |
| Heilongjiang | Bing | 3,695.70 | 0.00 | 0.00 | 0.00 |
| Heilongjiang | Boli | 4,314.84 | 0.00 | 0.00 | 0.00 |
| Heilongjiang | Daqing District | 4,998.84 | 0.00 | 0.00 | 0.00 |
| Heilongjiang | Duerbote Mongolian Autonomous County | 5,860.07 | 0.00 | 0.00 | 0.00 |
| Heilongjiang | Fangzheng | 2,861.69 | 0.00 | 0.00 | 0.00 |
| Heilongjiang | Fuyuan | 6,270.13 | 0.00 | 0.00 | 0.00 |
| Heilongjiang | Fujin District | 8,170.40 | 0.00 | 0.00 | 0.00 |
| Heilongjiang | Fuyu | 4,022.36 | 0.00 | 0.00 | 0.00 |
| Heilongjiang | Gannan | 4,811.77 | 0.00 | 0.00 | 0.00 |
| Heilongjiang | Haerbin District | 1,592.15 | 0.00 | 0.00 | 0.00 |
| Heilongjiang | Hailin District | 8,358.63 | 0.00 | 0.00 | 0.00 |
| Heilongjiang | Hailun District | 4,642.07 | 0.00 | 0.00 | 0.00 |
| Heilongjiang | Hegang District | 4,532.03 | 0.00 | 0.00 | 0.00 |
| Heilongjiang | Heihe District | 15,069.51 | 0.00 | 0.00 | 0.00 |
| Heilongjiang | Hulan | 2,544.89 | 0.00 | 0.00 | 0.00 |
| Heilongjiang | Huma | 35,028.98 | 0.00 | 0.00 | 0.00 |
| Heilongjiang | Hulin District | 9,026.41 | 0.00 | 0.00 | 0.00 |
| Heilongjiang | Huachuan | 2,697.79 | 0.00 | 0.00 | 0.00 |
| Heilongjiang | Huanan | 4,285.30 | 0.00 | 0.00 | 0.00 |
| Heilongjiang | Jidong | 3,098.00 | 0.00 | 0.00 | 0.00 |
| Heilongjiang | Jixi District | 2,094.29 | 0.00 | 0.00 | 0.00 |
| Heilongjiang | Jixian | 2,165.53 | 0.00 | 0.00 | 0.00 |
| Heilongjiang | Jiamusi District | 841.54 | 0.00 | 0.00 | 0.00 |
| Heilongjiang | Jiayin | 6,903.17 | 0.00 | 0.00 | 0.00 |
| Heilongjiang | Kedong | 2,078.07 | 0.00 | 0.00 | 0.00 |
| Heilongjiang | Keshan | 3,174.45 | 0.00 | 0.00 | 0.00 |
| Heilongjiang | Lanxi | 2,414.57 | 0.00 | 0.00 | 0.00 |
| Heilongjiang | Lindian | 3,469.25 | 0.00 | 0.00 | 0.00 |
| Heilongjiang | Linkou | 6,913.59 | 0.00 | 0.00 | 0.00 |
| Heilongjiang | Longjiang | 5,894.83 | 0.00 | 0.00 | 0.00 |
| Heilongjiang | Luobei | 6,721.88 | 0.00 | 0.00 | 0.00 |
| Heilongjiang | Mishan District | 7,357.24 | 0.00 | 0.00 | 0.00 |
| Heilongjiang | Mingshui | 2,269.20 | 0.00 | 0.00 | 0.00 |
| Heilongjiang | Mohe | 2,0566.44 | 0.00 | 0.00 | 0.00 |
| Heilongjiang | Mudanjiang District | 1,268.97 | 0.00 | 0.00 | 0.00 |
| Heilongjiang | Mulan | 3,093.94 | 0.00 | 0.00 | 0.00 |
| Heilongjiang | Muleng District | 5,972.43 | 0.00 | 0.00 | 0.00 |
| Heilongjiang | Nahe District | 6,713.78 | 0.00 | 0.00 | 0.00 |
| Heilongjiang | Nengjiang | 1,5604.09 | 0.00 | 0.00 | 0.00 |
| Heilongjiang | Ningan District | 6,686.56 | 0.00 | 0.00 | 0.00 |
| Heilongjiang | Qitaihe District | 1,707.98 | 0.00 | 0.00 | 0.00 |
| Heilongjiang | Qiqi Haer | 4,307.31 | 0.00 | 0.00 | 0.00 |
| Heilongjiang | Qinggang | 2,616.70 | 0.00 | 0.00 | 0.00 |
| Heilongjiang | Qingan | 5,418.17 | 0.00 | 0.00 | 0.00 |
| Heilongjiang | Raohe | 6,530.76 | 0.00 | 0.00 | 0.00 |
| Heilongjiang | Shangzhi District | 8,497.05 | 0.00 | 0.00 | 0.00 |
| Heilongjiang | Shuangcheng District | 2,980.43 | 0.00 | 0.00 | 0.00 |
| Heilongjiang | Shuangyashan District | 1,388.86 | 0.00 | 0.00 | 0.00 |
| Heilongjiang | Suibin | 3,371.37 | 0.00 | 0.00 | 0.00 |
| Heilongjiang | Suifenhe District | 378.78 | 0.00 | 0.00 | 0.00 |
| Heilongjiang | Suihua District | 2,696.63 | 0.00 | 0.00 | 0.00 |
| Heilongjiang | Suileng | 4,271.40 | 0.00 | 0.00 | 0.00 |
| Heilongjiang | Sunwu | 4,522.18 | 0.00 | 0.00 | 0.00 |
| Heilongjiang | Tahe | 15,463.93 | 0.00 | 0.00 | 0.00 |
| Heilongjiang | Tailai | 3,898.42 | 0.00 | 0.00 | 0.00 |
| Heilongjiang | Tangyuan | 3,973.71 | 0.00 | 0.00 | 0.00 |
| Heilongjiang | Tieli District | 6,363.38 | 0.00 | 0.00 | 0.00 |
| Heilongjiang | Tonghe | 5,518.36 | 0.00 | 0.00 | 0.00 |
| Heilongjiang | Tongjiang District | 6,066.26 | 0.00 | 0.00 | 0.00 |
| Heilongjiang | Wangkui | 2,287.16 | 0.00 | 0.00 | 0.00 |
| Heilongjiang | Wuchang District | 7,113.41 | 0.00 | 0.00 | 0.00 |
| Heilongjiang | Wudalianchi District | 9,096.49 | 0.00 | 0.00 | 0.00 |
| Heilongjiang | Xunke | 17,453.39 | 0.00 | 0.00 | 0.00 |
| Heilongjiang | Yanshou | 3,052.24 | 0.00 | 0.00 | 0.00 |
| Heilongjiang | Yichun District | 19,629.34 | 0.00 | 0.00 | 0.00 |
| Heilongjiang | Yian | 3,701.50 | 0.00 | 0.00 | 0.00 |
| Heilongjiang | Yilan | 4,516.97 | 0.00 | 0.00 | 0.00 |
| Heilongjiang | Youyi | 1,841.19 | 0.00 | 0.00 | 0.00 |
| Heilongjiang | Zhaodong District | 4,196.69 | 0.00 | 0.00 | 0.00 |
| Heilongjiang | Zhaoyuan | 3,985.87 | 0.00 | 0.00 | 0.00 |
| Heilongjiang | Zhaozhou | 2,335.81 | 0.00 | 0.00 | 0.00 |
| Henan | Anyang District | 0.00 | 0.00 | 237.82 | 27.93 |
| Henan | Anyang | 0.00 | 3.02 | 1,041.88 | 496.02 |
| Henan | Baofeng | 0.00 | 0.00 | 0.00 | 807.08 |
| Henan | Boai | 0.00 | 0.00 | 0.00 | 517.16 |
| Henan | Dancheng | 0.00 | 0.00 | 39.26 | 1,552.25 |
| Henan | Dengfeng District | 0.00 | 0.00 | 0.00 | 1,307.63 |
| Henan | Dengzhou District | 0.00 | 0.00 | 2,511.83 | 22.65 |
| Henan | Fan | 0.00 | 10.57 | 639.47 | 0.00 |
| Henan | Fangcheng | 0.00 | 0.00 | 0.00 | 2,662.07 |
| Henan | Fengqiu | 0.00 | 0.00 | 1,235.15 | 48.32 |
| Henan | Fugou | 0.00 | 0.00 | 0.00 | 1,250.25 |
| Henan | Kongyi District | 0.00 | 0.00 | 0.00 | 1,084.16 |
| Henan | Gushi District | 0.00 | 1,780.25 | 891.64 | 371.45 |
| Henan | Guangshan | 0.00 | 0.00 | 659.86 | 1,258.56 |
| Henan | Hebi District | 0.00 | 0.00 | 71.72 | 428.83 |
| Henan | Hua | 0.00 | 0.00 | 1,887.46 | 0.00 |
| Henan | Suibin | 0.00 | 656.84 | 609.27 | 0.00 |
| Henan | Suiyang | 0.00 | 0.00 | 0.00 | 1,571.12 |
| Henan | Hengchuan | 0.00 | 506.59 | 1,053.96 | 134.39 |
| Henan | Hui | 0.00 | 0.00 | 172.89 | 1,668.51 |
| Henan | Huojia | 0.00 | 0.00 | 80.03 | 415.24 |
| Henan | Jiyuan District | 0.00 | 0.00 | 5.28 | 2,024.11 |
| Henan | Jia | 0.00 | 0.00 | 0.00 | 770.08 |
| Henan | Jiaozuo District | 0.00 | 0.00 | 0.00 | 403.92 |
| Henan | Jun | 0.00 | 0.00 | 1,202.69 | 0.00 |
| Henan | Kaifeng District | 0.00 | 0.00 | 308.03 | 106.45 |
| Henan | Kaifeng | 0.00 | 0.00 | 1,232.89 | 332.95 |
| Henan | Lankao | 0.00 | 0.00 | 1,047.92 | 125.33 |
| Henan | Linzhou District | 0.00 | 8.30 | 687.79 | 1,559.04 |
| Henan | Linying | 0.00 | 0.00 | 0.00 | 841.05 |
| Henan | Lingbao District | 0.00 | 0.00 | 958.83 | 2,251.36 |
| Henan | Lushi | 0.00 | 0.00 | 55.87 | 3,810.40 |
| Henan | Lushan | 0.00 | 0.75 | 7.55 | 2,478.61 |
| Henan | Luyi | 0.00 | 0.00 | 109.47 | 1,244.21 |
| Henan | Luanchuan | 0.00 | 0.75 | 225.74 | 2,400.85 |
| Henan | Luoshan | 0.00 | 0.00 | 777.63 | 1,379.35 |
| Henan | Luoning | 0.00 | 0.75 | 95.88 | 2,326.10 |
| Henan | Luoyang District | 0.00 | 0.00 | 0.00 | 493.76 |
| Henan | Luohe District | 0.00 | 0.00 | 0.00 | 62.66 |
| Henan | Mengjin | 0.00 | 0.00 | 0.00 | 826.71 |
| Henan | Mengzhou District | 0.00 | 0.00 | 0.00 | 593.42 |
| Henan | Biyang | 0.00 | 0.00 | 31.71 | 2,921.03 |
| Henan | Yingchi | 0.00 | 0.00 | 23.40 | 1,419.37 |
| Henan | Mingquan | 0.00 | 0.00 | 767.06 | 547.36 |
| Henan | Nanle | 0.00 | 237.82 | 412.98 | 0.00 |
| Henan | Nanyang District | 0.00 | 0.00 | 155.53 | 1.51 |
| Henan | Nanzhao | 0.00 | 0.75 | 6.04 | 3,063.72 |
| Henan | Neihuang | 0.00 | 194.03 | 1,027.53 | 0.00 |
| Henan | Neixiang | 0.00 | 0.00 | 1,204.20 | 1,228.36 |
| Henan | Ningling | 0.00 | 0.00 | 852.38 | 3.77 |
| Henan | Pingdingshan District | 0.00 | 0.00 | 0.00 | 499.80 |
| Henan | Pingyu | 0.00 | 0.00 | 0.00 | 1,346.89 |
| Henan | Puyang District | 0.00 | 0.00 | 308.79 | 0.00 |
| Henan | Puyang | 0.00 | 0.00 | 1,534.88 | 0.00 |
| Henan | Qi | 0.00 | 0.00 | 286.14 | 343.52 |
| Henan | Qii | 0.00 | 0.00 | 973.93 | 364.66 |
| Henan | Qinyang | 0.00 | 0.00 | 0.00 | 632.68 |
| Henan | Qingfeng | 0.00 | 72.48 | 900.69 | 0.00 |
| Henan | Queshan | 0.00 | 0.00 | 15.85 | 2,305.72 |
| Henan | Runan | 0.00 | 0.00 | 6.79 | 1,680.59 |
| Henan | Ruyang | 0.00 | 0.00 | 0.00 | 1,405.78 |
| Henan | Ruzhou District | 0.00 | 0.00 | 0.00 | 1,662.47 |
| Henan | Sanmenxia District | 0.00 | 0.00 | 173.65 | 20.38 |
| Henan | Shan | 0.00 | 0.00 | 381.27 | 1,341.60 |
| Henan | Shangcheng | 0.00 | 0.75 | 384.29 | 1,805.92 |
| Henan | Shangqiu District | 0.00 | 0.00 | 87.58 | 9.06 |
| Henan | Shangshui | 0.00 | 0.00 | 0.00 | 1,404.27 |
| Henan | Shangcai | 0.00 | 0.00 | 0.00 | 1,577.91 |
| Henan | Sheqi | 0.00 | 0.00 | 345.78 | 831.99 |
| Henan | Shenqiu | 0.00 | 0.00 | 643.25 | 492.25 |
| Henan | Song | 0.00 | 0.75 | 64.17 | 3,139.97 |
| Henan | Sui | 0.00 | 0.00 | 973.17 | 0.00 |
| Henan | Suiping | 0.00 | 0.00 | 11.32 | 1,274.41 |
| Henan | Taiqian | 0.00 | 89.09 | 381.27 | 0.00 |
| Henan | Taikang | 0.00 | 0.00 | 297.46 | 1,598.30 |
| Henan | Tangyin | 0.00 | 0.00 | 693.07 | 11.32 |
| Henan | Tanghe | 0.00 | 0.00 | 2,325.35 | 289.91 |
| Henan | Tongxu | 0.00 | 0.00 | 218.19 | 611.54 |
| Henan | Tongbai | 0.00 | 0.00 | 43.03 | 1,961.45 |
| Henan | Weihui District | 0.00 | 0.00 | 576.05 | 351.82 |
| Henan | Weishi | 0.00 | 0.00 | 0.00 | 1,357.46 |
| Henan | Wen | 0.00 | 0.00 | 0.00 | 562.46 |
| Henan | Wuzhi | 0.00 | 0.00 | 0.00 | 927.87 |
| Henan | Wugan District | 0.00 | 0.00 | 0.00 | 686.28 |
| Henan | Wuyang | 0.00 | 0.00 | 0.00 | 797.26 |
| Henan | Xihua | 0.00 | 0.00 | 0.00 | 1,286.49 |
| Henan | Xiping | 0.00 | 0.00 | 34.73 | 1,113.60 |
| Henan | Xixia | 0.00 | 0.00 | 399.39 | 3,259.26 |
| Henan | Xi | 0.00 | 22.65 | 1935.02 | 4.53 |
| Henan | Xichuan | 0.00 | 0.00 | 1,729.67 | 1,186.83 |
| Henan | Xiayi | 0.00 | 0.00 | 1,415.59 | 151.75 |
| Henan | Xiangcheng | 0.00 | 0.00 | 0.00 | 982.99 |
| Henan | Xiangcheng | 0.00 | 0.00 | 4.53 | 1,133.98 |
| Henan | Xinan | 0.00 | 0.00 | 1.51 | 1,219.30 |
| Henan | Xincai | 0.00 | 0.00 | 872.01 | 649.29 |
| Henan | Xinmi District | 0.00 | 0.00 | 0.00 | 1,073.59 |
| Henan | Xin | 0.00 | 0.00 | 0.75 | 1,597.54 |
| Henan | Xinxinag District | 0.00 | 0.00 | 185.73 | 21.89 |
| Henan | Xinxiang | 0.00 | 0.00 | 426.57 | 138.16 |
| Henan | Xinye | 0.00 | 0.00 | 1,102.28 | 0.00 |
| Henan | Xinzheng District |  |  |  | 927.87 |
| Henan | Xinyang District | 0.00 | 0.00 | 48.32 | 180.44 |
| Henan | Xiuwu | 0.00 | 0.00 | 0.00 | 795.00 |
| Henan | Xuchang District | 0.00 | 0.00 | 0.00 | 108.72 |
| Henan | Yanling | 0.00 | 0.00 | 0.00 | 916.55 |
| Henan | Yanjin | 0.00 | 0.00 | 1,027.53 | 1,103.79 |
| Henan | Yancheng |  |  |  |  |
| Henan | Yanshi District | 0.00 | 0.00 | 0.00 | 984.50 |
| Henan | Ye | 0.00 | 0.00 | 0.00 | 1,515.25 |
| Henan | Yichuan | 0.00 | 0.00 | 0.00 | 1,130.96 |
| Henan | Yiyang | 0.00 | 0.00 | 1.51 | 1,781.76 |
| Henan | Yima District | 0.00 | 0.00 | 0.00 | 128.35 |
| Henan | Xingyang District | 0.00 | 0.00 | 0.00 | 988.27 |
| Henan | Yongcheng District | 0.00 | 0.00 | 1,888.97 | 295.20 |
| Henan | Yucheng | 0.00 | 0.00 | 1,241.95 | 407.69 |
| Henan | Yuzhou District | 0.00 | 0.00 | 0.00 | 1,563.57 |
| Henan | Yuanyang | 0.00 | 0.00 | 260.47 | 1,106.05 |
| Henan | Changge District | 0.00 | 0.00 | 0.00 | 690.05 |
| Henan | Changyuan | 0.00 | 0.00 | 1,170.98 | 0.00 |
| Henan | Tuocheng | 0.00 | 0.00 | 937.69 | 169.87 |
| Henan | Zhengping | 0.00 | 0.00 | 723.27 | 829.73 |
| Henan | Zhengyang | 0.00 | 0.00 | 1,122.66 | 862.19 |
| Henan | Zhengzhou District | 0.00 | 0.00 | 0.00 | 1,139.27 |
| Henan | Zhongmu | 0.00 | 0.00 | 0.00 | 1,524.31 |
| Henan | Zhoukou District | 0.00 | 0.00 | 0.00 | 90.60 |
| Hongkong | Hongkong | 15.89 | 0.00 | 0.00 | 0.00 |
| Hongkong | Hongkong | 1088.54 | 0.00 | 0.00 | 0.00 |
| Hubei | Anlu District | 0.00 | 0.00 | 124.98 | 1,249.05 |
| Hubei | Badong | 17.21 | 846.42 | 2,562.46 | 0.75 |
| Hubei | Baokang | 0.00 | 0.00 | 7.48 | 3,293.63 |
| Hubei | Chibi | 913.77 | 795.53 | 15.72 | 0.00 |
| Hubei | Chongyang | 1,599.29 | 380.93 | 0.75 | 0.00 |
| Hubei | Dawu | 0.00 | 2.99 | 658.58 | 1,354.57 |
| Hubei | Dazhi District | 301.60 | 1189.93 | 133.96 | 0.00 |
| Hubei | Danjiangkou District | 0.00 | 0.00 | 1,736.99 | 1,500.51 |
| Hubei | Dangyang District | 0.00 | 240.98 | 1,055.22 | 865.13 |
| Hubei | Ezhou District | 585.23 | 1,063.45 | 0.00 | 0.00 |
| Hubei | Enshi District | 176.62 | 3,239.74 | 606.19 | 0.00 |
| Hubei | Fang | 0.00 | 0.00 | 270.91 | 4,975.99 |
| Hubei | Gongan | 0.00 | 2,148.60 | 135.46 | 0.00 |
| Hubei | Gucheng | 0.00 | 0.00 | 924.25 | 1,688.35 |
| Hubei | Guangshui | 0.00 | 0.00 | 59.12 | 2,643.28 |
| Hubei | Hanchuan District | 0.00 | 1,133.05 | 550.81 | 0.00 |
| Hubei | Hanyang District | 0.00 | 1,121.08 | 32.18 | 0.00 |
| Hubei | Hefeng | 82.32 | 2,618.59 | 221.52 | 0.00 |
| Hubei | Hongan | 0.00 | 56.88 | 1,448.87 | 305.34 |
| Hubei | Honghu District | 19.46 | 2,347.67 | 160.90 | 0.00 |
| Hubei | Huangpi District | 0.00 | 228.26 | 1,658.41 | 416.10 |
| Hubei | Huanggang District | 0.00 | 452.77 | 20.95 | 0.00 |
| Hubei | Huangmei | 0.00 | 1,059.71 | 465.49 | 192.33 |
| Hubei | Huangshi District | 5.99 | 155.66 | 5.24 | 0.00 |
| Hubei | Jiayu | 246.22 | 791.04 | 0.00 | 0.00 |
| Hubei | Jianli | 0.00 | 1,601.54 | 1,552.14 | 0.00 |
| Hubei | Jianshi | 0.00 | 698.24 | 1,990.70 | 0.00 |
| Hubei | Jiangling | 0.00 | 2,343.18 | 123.48 | 0.00 |
| Hubei | Jingshan | 0.00 | 17.96 | 663.81 | 2,907.46 |
| Hubei | Jingmen District | 0.00 | 947.45 | 2,162.08 | 1,359.06 |
| Hubei | Laifeng | 1323.14 | 16.46 | 0.00 | 0.00 |
| Hubei | Laokouhe District | 0.00 | 0.00 | 1,094.13 | 12.72 |
| Hubei | Lichuan District | 413.86 | 4,244.82 | 0.00 | 0.00 |
| Hubei | Luotian | 0.00 | 0.00 | 1,650.18 | 520.13 |
| Hubei | Macheng District | 0.00 | 194.58 | 2,539.26 | 945.21 |
| Hubei | Nanzhang | 0.00 | 0.00 | 20.95 | 3,932.75 |
| Hubei | Qichun County | 0.00 | 820.97 | 1,357.56 | 234.24 |
| Hubei | Qianjing District | 0.00 | 1,698.08 | 346.50 | 0.00 |
| Hubei | Sha District | 0.00 | 147.43 | 0.00 | 0.00 |
| Hubei | Shennongjia District | 5.99 | 325.55 | 996.84 | 1,976.48 |
| Hubei | Shiyan District | 0.00 | 0.00 | 720.69 | 526.11 |
| Hubei | Shishou District | 0.00 | 512.64 | 893.57 | 16.46 |
| Hubei | Songchi District | 0.00 | 1,053.72 | 927.25 | 214.79 |
| Hubei | Suizhou District | 0.00 | 0.00 | 1.50 | 7,156.78 |
| Hubei | Tianmen District | 0.00 | 869.62 | 1,802.85 | 5.99 |
| Hubei | Tongcheng | 361.47 | 723.69 | 16.46 | 0.00 |
| Hubei | Tongshan | 1229.59 | 1,174.21 | 14.97 | 0.00 |
| Hubei | Tuanfeng | 0.00 | 251.46 | 284.39 | 168.39 |
| Hubei | Wufeng Tujia Autonomous County | 0.00 | 558.29 | 1,840.27 | 29.19 |
| Hubei | Wuchang District | 904.05 | 1,126.31 | 0.75 | 0.00 |
| Hubei | Wuhan District | 1.50 | 1,603.78 | 57.63 | 0.00 |
| Hubei | Wuxue District | 0.00 | 822.47 | 360.72 | 52.39 |
| Hubei | Xishui | 32.93 | 877.85 | 1,009.57 | 74.84 |
| Hubei | Xiantao District | 0.00 | 1,763.19 | 782.06 | 0.00 |
| Hubei | Xianfeng | 395.89 | 2,175.55 | 0.00 | 0.00 |
| Hubei | Xianning District | 922.01 | 564.28 | 23.20 | 0.00 |
| Hubei | Xiaogan District | 0.00 | 168.39 | 1,265.51 | 817.23 |
| Hubei | Xinzhou District | 0.00 | 647.35 | 790.29 | 45.65 |
| Hubei | Xinshan | 0.00 | 233.50 | 1,561.87 | 568.02 |
| Hubei | Enshi | 701.23 | 2,069.28 | 10.48 | 0.00 |
| Hubei | Yangxin | 62.12 | 2,378.36 | 342.76 | 0.00 |
| Hubei | Yichang District | 6.74 | 279.15 | 35.17 | 0.00 |
| Hubei | Yichang | 3.74 | 671.30 | 1,910.62 | 1,209.38 |
| Hubei | Yicheng District | 0.00 | 0.00 | 166.14 | 2,014.64 |
| Hubei | Yidu District | 0.00 | 487.94 | 650.34 | 235.74 |
| Hubei | Yingshan | 0.00 | 79.33 | 1,262.52 | 133.21 |
| Hubei | Yingcheng District | 0.00 | 34.43 | 922.01 | 147.43 |
| Hubei | Yuanan | 0.00 | 0.00 | 208.80 | 1,569.36 |
| Hubei | Yunmeng | 0.00 | 101.78 | 490.19 | 32.93 |
| Hubei | Yunxi | 0.00 | 93.55 | 1,762.44 | 1,777.41 |
| Hubei | Yun | 0.00 | 37.42 | 2,166.57 | 1,743.73 |
| Hubei | ZaoyangDistrict | 0.00 | 0.00 | 910.03 | 2,459.18 |
| Hubei | Changyang Tujia Autonomous County | 0.00 | 1,353.07 | 2,096.97 | 0.00 |
| Hubei | Zhijiang District | 0.00 | 883.84 | 689.26 | 15.72 |
| Hubei | Zhongxiang District | 0.00 | 20.95 | 2,254.13 | 2,245.89 |
| Hubei | Zhuxhan | 0.00 | 9.73 | 1,422.67 | 2,262.36 |
| Hubei | Zhuxi | 0.00 | 38.17 | 1,299.94 | 2,068.53 |
| Hubei | Zigui | 34.43 | 864.38 | 1,440.63 | 0.00 |
| Hubei | Xiangfan District | 0.00 | 0.00 | 193.08 | 88.31 |
| Hunan | Anhua | 1,258.91 | 2,766.95 | 1,040.24 | 8.59 |
| Hunan | Anren | 1,484.61 | 19.52 | 0.00 | 0.00 |
| Hunan | Anxiang | 0.00 | 1,108.18 | 24.99 | 0.00 |
| Hunan | Baojing | 183.53 | 1,626.74 | 0.00 | 0.00 |
| Hunan | Chaling | 2548.28 | 8.59 | 0.00 | 0.00 |
| Hunan | Changde District | 0.00 | 2,328.83 | 521.68 | 0.00 |
| Hunan | Changning District | 862.18 | 1,236.26 | 0.00 | 0.00 |
| Hunan | Chengzhou District | 272.56 | 5.47 | 0.00 | 0.00 |
| Hunan | Chenxi | 1,559.58 | 479.51 | 0.00 | 0.00 |
| Hunan | Chengbu Miao Autonomous County | 1,508.82 | 1,158.95 | 0.00 | 0.00 |
| Hunan | Cili | 32.80 | 3,051.22 | 550.58 | 1.56 |
| Hunan | Dao | 2,371.78 | 111.68 | 0.00 | 0.00 |
| Hunan | Dongan | 869.21 | 1,365.90 | 0.00 | 0.00 |
| Hunan | Dongkou | 1,147.23 | 1,074.60 | 0.00 | 0.00 |
| Hunan | Fenghuang | 1,313.58 | 483.42 | 0.00 | 0.00 |
| Hunan | Guzhang | 88.25 | 1,260.47 | 0.00 | 0.00 |
| Hunan | Guidong | 1,286.24 | 160.88 | 0.00 | 0.00 |
| Hunan | Guiyang | 2,362.41 | 641.17 | 0.00 | 0.00 |
| Hunan | Hanshou | 0.00 | 1,678.29 | 534.18 | 39.83 |
| Hunan | Hengdong | 522.46 | 1,449.47 | 0.00 | 0.00 |
| Hunan | Hengyang | 1,027.75 | 1,725.93 | 0.00 | 0.00 |
| Hunan | Hengshan | 117.93 | 855.15 | 0.00 | 0.00 |
| Hunan | Hengyang District | 337.38 | 2,909.08 | 0.00 | 0.00 |
| Hunan | Hengjiang District | 1,362.78 | 974.64 | 0.00 | 0.00 |
| Hunan | Huayuan | 257.72 | 892.64 | 0.00 | 0.00 |
| Hunan | Huarong | 0.00 | 1,032.43 | 791.90 | 71.07 |
| Hunan | Huahua District | 1,716.55 | 513.87 | 0.00 | 0.00 |
| Hunan | Jishou District | 690.37 | 400.63 | 0.00 | 0.00 |
| Hunan | Jiahe | 715.36 | 0.00 | 0.00 | 0.00 |
| Hunan | Jianghua Yao Autonomous County | 3,256.61 | 22.65 | 0.00 | 0.00 |
| Hunan | Jiangyong | 1,661.11 | 0.00 | 0.00 | 0.00 |
| Hunan | Jingshi District | 0.00 | 578.69 | 42.17 | 0.00 |
| Hunan | Jingzhou Miao and Dong Autonomous County | 1,868.06 | 363.93 | 0.00 | 0.00 |
| Hunan | Lanshan | 1,800.12 | 0.00 | 0.00 | 0.00 |
| Hunan | Leiyang District | 1,137.08 | 1,591.60 | 0.00 | 0.00 |
| Hunan | Lengshuijiang District | 139.01 | 298.33 | 0.00 | 0.00 |
| Hunan | Lengshuitan District | 372.52 | 891.08 | 0.00 | 0.00 |
| Hunan | Li | 0.00 | 1,443.22 | 653.67 | 0.00 |
| Hunan | Liling | 306.14 | 1,895.39 | 0.78 | 0.00 |
| Hunan | Lianyuan District | 449.05 | 1,495.54 | 19.52 | 0.00 |
| Hunan | Linli | 0.00 | 892.64 | 389.70 | 0.00 |
| Hunan | Linwu | 1392.46 | 0.00 | 0.00 | 0.00 |
| Hunan | Linyin District | 0.00 | 685.68 | 1,084.76 | 3.12 |
| Hunan | Liuyang District | 3,562.75 | 1,616.59 | 0.00 | 0.00 |
| Hunan | Longhshan | 1,972.71 | 1,305.77 | 0.00 | 0.00 |
| Hunan | Longhui | 937.15 | 1,972.71 | 117.93 |  |
| Hunan | Loudi District | 0.00 | 424.84 | 3.90 | 0.00 |
| Hunan | Luxi | 1,313.58 | 301.45 | 0.00 | 0.00 |
| Hunan | Mayang Miao Autonomous County | 1,551.77 | 68.72 | 0.00 | 0.00 |
| Hunan | Miluo District | 0.00 | 1,154.26 | 530.27 | 0.00 |
| Hunan | Nan | 0.00 | 1,376.05 | 3.12 | 0.00 |
| Hunan | Ningxiang | 0.00 | 2139.84 | 860.62 | 0.78 |
| Hunan | Ningyuan | 2,331.95 | 223.36 | 0.00 | 0.00 |
| Hunan | Pingjiang | 1,073.82 | 2,569.36 | 609.15 | 0.00 |
| Hunan | Qidong | 996.51 | 920.75 | 0.00 | 0.00 |
| Hunan | Qiyang | 904.35 | 1,640.02 | 11.71 | 0.00 |
| Hunan | Rucheng | 2,324.92 | 124.95 | 0.00 | 0.00 |
| Hunan | Sangzhi | 1,831.36 | 1,674.38 | 107.77 | 0.00 |
| Hunan | Sahoshan District | 0.00 | 235.85 | 2.34 | 0.00 |
| Hunan | Shaodong | 10.93 | 1,807.15 | 0.00 | 0.00 |
| Hunan | Shaoyang District | 0.78 | 458.42 | 0.00 | 0.00 |
| Hunan | Shaoyang | 911.38 | 1,126.15 | 0.00 | 0.00 |
| Hunan | Shimen | 0.00 | 1,711.09 | 2,451.44 | 21.87 |
| Hunan | Shuangfeng | 0.00 | 1,766.54 | 0.00 | 0.00 |
| Hunan | Shuangpai | 1,028.53 | 729.42 | 25.77 | 0.00 |
| Hunan | Suining | 1,615.81 | 1,342.47 | 0.00 | 0.00 |
| Hunan | Taojiang | 0.00 | 176.50 | 1,723.58 | 241.32 |
| Hunan | Taoyuan | 380.33 | 4,129.73 | 131.20 | 0.00 |
| Hunan | Channel Dong Autonomous County | 2,267.13 | 3.90 | 0.00 | 0.00 |
| Hunan | Wangcheng | 0.00 | 797.36 | 669.28 | 0.00 |
| Hunan | Wugang District | 1,438.53 | 132.76 | 0.00 | 0.00 |
| Hunan | Xiangtan District | 32.02 | 249.13 | 0.00 | 0.00 |
| Hunan | Xiangtan District | 205.39 | 2,423.32 | 0.00 | 0.00 |
| Hunan | Xiangyin District | 22.65 | 2,020.35 | 12.50 | 0.00 |
| Hunan | Xiangyin | 0.00 | 1,309.67 | 363.93 | 0.00 |
| Hunan | Xinhua | 569.32 | 3,065.28 | 132.76 | 0.00 |
| Hunan | Xinhuang Dong Autonomous County | 573.23 | 973.86 | 0.00 | 0.00 |
| Hunan | Xinning | 1562.70 | 1,259.69 | 0.00 | 0.00 |
| Hunan | Xinshao | 82.00 | 1,764.97 | 0.00 | 0.00 |
| Hunan | Xintian | 935.59 | 74.19 | 0.00 | 0.00 |
| Hunan | Xupu | 1,500.23 | 1,922.73 | 121.83 | 0.00 |
| Hunan | Yanling | 2,056.27 | 2.34 | 0.00 | 0.00 |
| Hunan | Yiyang District | 0.00 | 227.26 | 153.85 | 19.52 |
| Hunan | Yiyang District | 0.00 | 760.66 | 691.15 | 42.95 |
| Hunan | Yongshun | 909.82 | 3,069.18 | 0.00 | 0.00 |
| Hunan | Yongxin | 1,827.45 | 164.78 | 0.00 | 0.00 |
| Hunan | Yongzhou District | 675.53 | 1,189.41 | 112.46 | 0.00 |
| Hunan | You | 1,850.10 | 894.98 | 0.78 | 0.00 |
| Hunan | Yuanjiang | 0.00 | 2,134.37 | 21.87 | 0.00 |
| Hunan | Yuanling | 4492.87 | 1,524.44 | 0.00 | 0.00 |
| Hunan | Yueyang District | 0.00 | 459.99 | 410.79 | 14.06 |
| Hunan | Yueyang | 0.00 | 2,035.19 | 1,238.61 | 14.06 |
| Hunan | Zhangjiajie District | 871.55 | 1,865.72 | 0.00 | 0.00 |
| Hunan | Xhengsha District | 42.95 | 313.17 | 3.90 | 0.00 |
| Hunan | Shangsha | 436.56 | 1,688.44 | 56.23 | 0.00 |
| Hunan | Zhijiang Dong Autonomous County | 1,479.92 | 670.07 | 0.00 | 0.00 |
| Hunan | Zhuzhou District | 3.90 | 417.81 | 0.00 | 0.00 |
| Hunan | Zhuzhou | 155.41 | 1,397.92 | 0.00 | 0.00 |
| Hunan | Zixing District | 2,788.03 | 1.56 | 0.00 | 0.00 |
| Inner Mongolia | Aohan Qi | 7,923.83 | 535.55 | 0.00 | 0.00 |
| Inner Mongolia | Aba Gaqi | 2,8833.65 | 0.00 | 0.00 | 0.00 |
| Inner Mongolia | Alxa Youqi | 7,1945.86 | 0.00 | 0.00 | 0.00 |
| Inner Mongolia | Ala Shanzuo Qi | 78,946.88 | 0.00 | 0.00 | 0.00 |
| Inner Mongolia | Alu keerqin Qi | 13,403.03 | 0.00 | 0.00 | 0.00 |
| Inner Mongolia | Arong Qi | 12,559.88 | 0.00 | 0.00 | 0.00 |
| Inner Mongolia | Balin Youqi | 10,225.99 | 0.00 | 0.00 | 0.00 |
| Inner Mongolia | Balin Zuoqi | 6,786.67 | 0.00 | 0.00 | 0.00 |
| Inner Mongolia | Baotou District | 2,543.71 | 35.62 | 0.00 | 0.00 |
| Inner Mongolia | Chahaer Zuoyi Houqi | 3,879.67 | 0.00 | 0.00 | 0.00 |
| Inner Mongolia | Chahaer Zuoyi Zhongqi | 4,186.63 | 0.00 | 0.00 | 0.00 |
| Inner Mongolia | Chenbaer Huqi | 20,779.65 | 0.00 | 0.00 | 0.00 |
| Inner Mongolia | Chifeng District | 7,142.20 | 0.00 | 0.00 | 0.00 |
| Inner Mongolia | Dala Teqi | 5,384.01 | 2,802.09 | 0.00 | 0.00 |
| Inner Mongolia | Dengkou | 3,210.72 | 0.00 | 0.00 | 0.00 |
| Inner Mongolia | Dongsheng City | 658.59 | 1,493.33 | 0.00 | 0.00 |
| Inner Mongolia | Dongwu Zhumuqin Qi | 48,607.60 | 0.00 | 0.00 | 0.00 |
| Inner Mongolia | Duolun | 4,014.37 | 0.00 | 0.00 | 0.00 |
| Inner Mongolia | Ergun city | 58,461.23 | 0.00 | 0.00 | 0.00 |
| Inner Mongolia | Ejina Qi | 87,887.42 | 0.00 | 0.00 | 0.00 |
| Inner Mongolia | Oroqen Autonomous Qi | 64,687.74 | 0.00 | 0.00 | 0.00 |
| Inner Mongolia | Etuoke Qi | 19,324.53 | 170.31 | 0.00 | 0.00 |
| Inner Mongolia | Etuoke Qianqi | 11,166.93 | 1,066.57 | 0.00 | 0.00 |
| Inner Mongolia | Ewenki Autonomous Qi | 21,067.18 | 0.00 | 0.00 | 0.00 |
| Inner Mongolia | Erenhot City | 191.04 | 0.00 | 0.00 | 0.00 |
| Inner Mongolia | Fengzheng | 2,440.09 | 216.94 | 0.00 | 0.00 |
| Inner Mongolia | Guyang | 5,222.11 | 0.00 | 0.00 | 0.00 |
| Inner Mongolia | Hailar City | 1,582.69 | 0.00 | 0.00 | 0.00 |
| Inner Mongolia | Hangjin Houqi | 1,754.30 | 0.00 | 0.00 | 0.00 |
| Inner Mongolia | Hangjin Qi | 18,414.03 | 0.00 | 0.00 | 0.00 |
| Inner Mongolia | Helin Geer | 2,785.90 | 585.42 | 0.00 | 0.00 |
| Inner Mongolia | Huhehaote District | 2,047.01 | 0.00 | 0.00 | 0.00 |
| Inner Mongolia | Huade | 2,663.51 | 0.00 | 0.00 | 0.00 |
| Inner Mongolia | Huolinguole city | 661.83 | 0.00 | 0.00 | 0.00 |
| Inner Mongolia | Jining City | 101.67 | 0.00 | 0.00 | 0.00 |
| Inner Mongolia | Harqin Qi | 3,098.69 | 59.58 | 0.00 | 0.00 |
| Inner Mongolia | Kailu | 4,399.03 | 0.00 | 0.00 | 0.00 |
| Inner Mongolia | Keerqin Youyi Qianqi | 28,109.65 | 0.00 | 0.00 | 0.00 |
| Inner Mongolia | Keerqin Youyi Zhongqi | 13,168.61 | 0.00 | 0.00 | 0.00 |
| Inner Mongolia | Keerqin Zuoyi Houqi | 11,959.57 | 0.00 | 0.00 | 0.00 |
| Inner Mongolia | Keerqin Zuoyi Zhongqi | 10,036.89 | 0.00 | 0.00 | 0.00 |
| Inner Mongolia | Keshenke Tengqi | 18,228.17 | 0.00 | 0.00 | 0.00 |
| Inner Mongolia | Kulun Qi | 4,478.69 | 296.59 | 0.00 | 0.00 |
| Inner Mongolia | Liangcheng | 3,357.07 | 49.86 | 0.00 | 0.00 |
| Inner Mongolia | Linxi | 4,066.82 | 0.00 | 0.00 | 0.00 |
| Inner Mongolia | Linhe City | 2,392.82 | 0.00 | 0.00 | 0.00 |
| Inner Mongolia | Manzhouli City | 818.55 | 0.00 | 0.00 | 0.00 |
| Inner Mongolia | Dole Dawar Autonomous Qi | 11,703.77 | 0.00 | 0.00 | 0.00 |
| Inner Mongolia | Laiman Qi | 7,903.75 | 512.89 | 0.00 | 0.00 |
| Inner Mongolia | Ningcheng | 2,332.60 | 2015.93 | 0.00 | 0.00 |
| Inner Mongolia | Qingshuihe | 1,265.38 | 1,481.67 | 0.00 | 0.00 |
| Inner Mongolia | Shangdu | 4,253.33 | 0.00 | 0.00 | 0.00 |
| Inner Mongolia | Sizi Wangqi | 24,637.31 | 0.00 | 0.00 | 0.00 |
| Inner Mongolia | Suni Teyou Qi | 25,922.76 | 0.00 | 0.00 | 0.00 |
| Inner Mongolia | Suni Tezuo Qi | 36,365.69 | 0.00 | 0.00 | 0.00 |
| Inner Mongolia | Taipusi Qi | 3,530.62 | 0.00 | 0.00 | 0.00 |
| Inner Mongolia | Tongliao | 3,633.59 | 0.00 | 0.00 | 0.00 |
| Inner Mongolia | Tuquan | 5,213.05 | 0.00 | 0.00 | 0.00 |
| Inner Mongolia | Tumote Youqi | 2,031.47 | 308.25 | 0.00 | 0.00 |
| Inner Mongolia | Tumote Zuoqi | 2,758.06 | 0.00 | 0.00 | 0.00 |
| Inner Mongolia | Tuoketuo | 1,350.21 | 16.19 | 0.00 | 0.00 |
| Inner Mongolia | Wengniu Teqi | 12,210.83 | 0.00 | 0.00 | 0.00 |
| Inner Mongolia | Wuhai District | 1511.46 | 0.00 | 0.00 | 0.00 |
| Inner Mongolia | Wulate Houqi | 24,340.71 | 0.00 | 0.00 | 0.00 |
| Inner Mongolia | Wulate Qianqi | 7,369.50 | 0.00 | 0.00 | 0.00 |
| Inner Mongolia | Wulate Zhongqi | 23,098.65 | 0.00 | 0.00 | 0.00 |
| Inner Mongolia | Wulan Haote City | 886.54 | 0.00 | 0.00 | 0.00 |
| Inner Mongolia | Wushen | 6,799.62 | 4,332.98 | 0.00 | 0.00 |
| Inner Mongolia | Wuyuan | 2,459.52 | 0.00 | 0.00 | 0.00 |
| Inner Mongolia | Wuchuan | 4,713.11 | 0.00 | 0.00 | 0.00 |
| Inner Mongolia | Xiwu Zhumu Qinqi | 25,000.60 | 0.00 | 0.00 | 0.00 |
| Inner Mongolia | Xilin Haote City | 16,506.25 | 0.00 | 0.00 | 0.00 |
| Inner Mongolia | Xianghuang Qi | 5,113.97 | 0.00 | 0.00 | 0.00 |
| Inner Mongolia | Xinbaer Huyou Qi | 28,498.85 | 0.00 | 0.00 | 0.00 |
| Inner Mongolia | Xinbaer Huzuo Qi | 22,362.99 | 0.00 | 0.00 | 0.00 |
| Inner Mongolia | Xinghe | 3,420.54 | 110.09 | 0.00 | 0.00 |
| Inner Mongolia | Yashike City | 32,201.08 | 0.00 | 0.00 | 0.00 |
| Inner Mongolia | Yijin Huoluo Qi | 1,130.68 | 4,623.74 | 0.00 | 0.00 |
| Inner Mongolia | Zhalai Teqi | 12,407.05 | 0.00 | 0.00 | 0.00 |
| Inner Mongolia | Zhalantun City | 18,847.91 | 0.00 | 0.00 | 0.00 |
| Inner Mongolia | Zhalu Teqi | 17,547.56 | 0.00 | 0.00 | 0.00 |
| Inner Mongolia | Zhenglan Qi | 10,522.58 | 0.00 | 0.00 | 0.00 |
| Inner Mongolia | Zhengxiang Baiqi | 6,392.29 | 0.00 | 0.00 | 0.00 |
| Inner Mongolia | Zhunge Erqi | 230.54 | 7,135.07 | 0.00 | 0.00 |
| Inner Mongolia | Zuozi | 3,070.19 | 0.00 | 0.00 | 0.00 |
| Jiangsu | Baoying | 0.00 | 1,722.16 | 0.00 | 0.00 |
| Jiangsu | Binhai | 0.00 | 2,256.24 | 0.00 | 0.00 |
| Jiangsu | Changshu | 1.70 | 1,301.19 | 5.10 | 0.00 |
| Jiangsu | Changshu City | 0.00 | 128.42 | 0.00 | 0.00 |
| Jiangsu | Chibi City | 0.00 | 2,830.30 | 0.00 | 0.00 |
| Jiangsu | Dafeng City | 0.00 | 2,572.61 | 0.00 | 0.00 |
| Jiangsu | Dantu | 0.00 | 907.43 | 94.40 | 0.00 |
| Jiangsu | Danyang | 0.00 | 1,204.24 | 13.61 | 0.00 |
| Jiangsu | Donghai | 0.00 | 1,431.31 | 929.54 | 369.95 |
| Jiangsu | Dongtai City | 0.00 | 2,517.33 | 0.00 | 0.00 |
| Jiangsu | Feng | 0.00 | 0.00 | 1,607.35 | 130.97 |
| Jiangsu | Funing | 0.00 | 1,724.71 | 0.00 | 0.00 |
| Jiangsu | Ganyu | 0.00 | 980.57 | 610.62 | 94.40 |
| Jiangsu | Gaochun | 0.00 | 6.80 | 914.23 | 0.85 |
| Jiangsu | Gaoyou | 0.00 | 2,311.52 | 0.00 | 0.00 |
| Jiangsu | Guannan | 0.00 | 1,182.12 | 49.33 | 0.00 |
| Jiangsu | Guanyun | 0.00 | 2,150.79 | 18.71 | 1.70 |
| Jiangsu | Haian | 0.00 | 1,330.95 | 0.00 | 0.00 |
| Jiangsu | Haimen City | 0.00 | 1,098.78 | 0.00 | 0.00 |
| Jiangsu | Hanjiang | 0.00 | 950.80 | 3.40 | 0.00 |
| Jiangsu | Hongze | 0.00 | 1,478.08 | 0.85 | 0.00 |
| Jiangsu | Huaian City | 0.00 | 1,855.68 | 11.06 | 0.00 |
| Jiangsu | Huaiyin City | 0.00 | 403.96 | 0.85 | 0.00 |
| Jiangsu | Huaiyin | 0.00 | 1,577.58 | 1.70 | 0.00 |
| Jiangsu | Jianhu | 0.00 | 1,369.22 | 0.00 | 0.00 |
| Jiangsu | Jiangdu City | 0.00 | 1,558.87 | 0.00 | 0.00 |
| Jiangsu | Jiangning | 0.00 | 1,601.40 | 250.03 | 0.00 |
| Jiangsu | Jiangpu | 0.00 | 773.91 | 105.46 | 0.00 |
| Jiangsu | JiangyinCity | 0.85 | 1,114.09 | 5.95 | 0.00 |
| Jiangsu | Jiangyan City | 0.00 | 1,409.19 | 0.00 | 0.00 |
| Jiangsu | Jinhu | 0.00 | 1,562.28 | 0.00 | 0.00 |
| Jiangsu | Jintan City | 0.00 | 1,114.94 | 29.77 | 0.00 |
| Jiangsu | Jingjiang City | 0.00 | 678.66 | 0.00 | 0.00 |
| Jiangsu | Jurong City | 0.00 | 1,177.87 | 431.18 | 0.00 |
| Jiangsu | Kunshan City | 68.04 | 1,033.30 | 0.00 | 0.00 |
| Jiangsu | Lishui | 0.85 | 1,163.41 | 71.44 | 0.00 |
| Jiangsu | Liyang | 0.00 | 1,572.48 | 207.51 | 0.00 |
| Jiangsu | Lianyungang City | 0.00 | 625.93 | 34.87 | 127.57 |
| Jiangsu | Lianshui | 0.00 | 1,608.20 | 403.96 | 0.00 |
| Jiangsu | Liuhe | 0.00 | 1,330.10 | 306.16 | 0.00 |
| Jiangsu | Nanjing City | 0.00 | 1,123.44 | 59.53 | 0.00 |
| Jiangsu | Nantong | 0.00 | 1,883.75 | 0.00 | 0.00 |
| Jiangsu | Nantong City | 0.00 | 174.34 | 0.00 | 0.00 |
| Jiangsu | Pei | 0.00 | 0.00 | 1,327.55 | 256.84 |
| Jiangsu | Pizhou | 0.00 | 0.00 | 2,202.66 | 274.70 |
| Jiangsu | Qidong City | 0.00 | 1,091.98 | 0.00 | 0.00 |
| Jiangsu | Rudong | 0.00 | 2,038.53 | 0.00 | 0.00 |
| Jiangsu | Rugao | 0.00 | 1,803.80 | 0.00 | 0.00 |
| Jiangsu | Sheyang | 0.00 | 3,017.39 | 0.00 | 0.00 |
| Jiangsu | Shuyang | 0.00 | 2,738.45 | 26.36 | 0.00 |
| Jiangsu | Sihong | 0.00 | 3,056.52 | 255.13 | 0.00 |
| Jiangsu | Siyang | 0.00 | 1,726.41 | 353.79 | 0.00 |
| Jiangsu | Suzhou City | 4.25 | 164.99 | 0.00 | 0.00 |
| Jiangsu | Suining | 0.00 | 79.09 | 2,027.47 | 22.11 |
| Jiangsu | Taicang | 2.55 | 713.53 | 0.00 | 0.00 |
| Jiangsu | Taixing City | 0.00 | 1,665.18 | 0.00 | 0.00 |
| Jiangsu | Taizhou City | 0.00 | 150.53 | 0.00 | 0.00 |
| Jiangsu | Wuxi City | 0.00 | 188.80 | 11.06 | 0.00 |
| Jiangsu | Wujiang City | 750.10 | 652.29 | 0.00 | 0.00 |
| Jiangsu | Xishan City | 0.00 | 1,075.82 | 0.00 | 0.00 |
| Jiangsu | Xishan City | 0.00 | 5.10 | 0.00 | 0.00 |
| Jiangsu | Xiangshui | 0.00 | 1,555.47 | 86.75 | 0.00 |
| Jiangsu | Xinyi City | 0.00 | 307.01 | 1,600.55 | 0.00 |
| Jiangsu | Suqian City | 0.00 | 545.99 | 1,541.86 | 0.00 |
| Jiangsu | Xuyi | 0.00 | 2,358.30 | 591.06 | 0.00 |
| Jiangsu | Xuzhou District | 0.00 | 0.00 | 161.59 | 46.77 |
| Jiangsu | Yancheng District | 0.00 | 2,045.33 | 0.00 | 0.00 |
| Jiangsu | Yangzhong City | 0.00 | 388.66 | 0.00 | 0.00 |
| Jiangsu | Yangzhou District | 0.00 | 182.85 | 0.00 | 0.00 |
| Jiangsu | Yizheng City | 0.00 | 863.21 | 196.45 | 0.00 |
| Jiangsu | Yixing City | 0.00 | 1,740.02 | 390.36 | 8.50 |
| Jiangsu | Zhangjiagang City | 0.00 | 871.71 | 0.00 | 0.00 |
| Jiangsu | Zhengjiang District | 0.00 | 244.93 | 27.21 | 0.00 |
| Jiangxi | Anfu | 2,779.77 | 19.85 | 0.00 | 0.00 |
| Jiangxi | Anyi | 6.11 | 609.24 | 93.91 | 0.00 |
| Jiangxi | Anyuan | 2,324.75 | 0.00 | 0.00 | 0.00 |
| Jiangxi | Boyang | 3,834.88 | 440.52 | 0.00 | 0.00 |
| Jiangxi | Chongyang | 1,123.05 | 413.03 | 0.00 | 0.00 |
| Jiangxi | Chongyi | 1,951.41 | 226.75 | 0.00 | 0.00 |
| Jiangxi | Dayu | 1,213.91 | 119.10 | 0.00 | 0.00 |
| Jiangxi | Dean | 0.00 | 493.96 | 438.23 | 0.76 |
| Jiangxi | Dexing City | 2,103.34 | 29.01 | 0.00 | 0.00 |
| Jiangxi | Dingnan | 1,286.44 | 0.00 | 0.00 | 0.00 |
| Jiangxi | Dongxiang | 1,122.29 | 146.58 | 0.00 | 0.00 |
| Jiangxi | Duchang | 1,652.14 | 384.02 | 0.00 | 0.00 |
| Jiangxi | Fenyi | 1,388.74 | 9.16 | 0.00 | 0.00 |
| Jiangxi | Fengcheng City | 703.91 | 2,164.42 | 7.63 | 0.00 |
| Jiangxi | Fengxin | 696.28 | 981.05 | 0.00 | 0.00 |
| Jiangxi | Gan | 2,896.58 | 104.59 | 0.00 | 0.00 |
| Jiangxi | Ganzhou City | 419.90 | 13.74 | 0.00 | 0.00 |
| Jiangxi | Gaoan | 141.24 | 2,307.95 | 0.00 | 0.00 |
| Jiangxi | Guangchang | 1,586.48 | 0.00 | 0.00 | 0.00 |
| Jiangxi | Guangfeng | 1,386.45 | 0.00 | 0.00 | 0.00 |
| Jiangxi | Guixi | 2,422.47 | 80.93 | 0.00 | 0.00 |
| Jiangxi | Hengfeng | 655.82 | 0.00 | 0.00 | 0.00 |
| Jiangxi | Hukou | 423.72 | 258.05 | 0.00 | 0.00 |
| Jiangxi | Huichang | 2,665.25 | 0.00 | 0.00 | 0.00 |
| Jiangxi | Jian City | 131.32 | 1.53 | 0.00 | 0.00 |
| Jiangxi | Jian | 3,069.12 | 39.70 | 0.00 | 0.00 |
| Jiangxi | Jishui | 2,139.22 | 590.16 | 0.00 | 0.00 |
| Jiangxi | Jinxi | 1,331.48 | 39.70 | 0.00 | 0.00 |
| Jiangxi | Jinxian | 650.47 | 1,291.78 | 0.00 | 0.00 |
| Jiangxi | Jingangshan City | 648.18 | 27.48 | 0.00 | 0.00 |
| Jiangxi | Jindezheng District | 2,965.29 | 109.94 | 0.00 | 0.00 |
| Jiangxi | Jingan | 66.42 | 1,262.77 | 96.20 | 0.00 |
| Jiangxi | Jiujiang District | 48.86 | 564.96 | 120.63 | 0.00 |
| Jiangxi | Jiujiang | 11.45 | 672.61 | 154.98 | 0.00 |
| Jiangxi | Lean | 2,039.21 | 389.37 | 0.00 | 0.00 |
| Jiangxi | Leping | 2,298.79 | 0.00 | 0.00 | 0.00 |
| Jiangxi | Lichuan | 1,720.85 | 0.00 | 0.00 | 0.00 |
| Jiangxi | Lianhua | 1,035.26 | 4.58 | 0.00 | 0.00 |
| Jiangxi | Linchuan City | 1,532.27 | 611.53 | 0.00 | 0.00 |
| Jiangxi | Longnan | 1,607.85 | 0.00 | 0.00 | 0.00 |
| Jiangxi | Nanchang District | 19.09 | 236.67 | 156.51 | 0.00 |
| Jiangxi | Nanchang | 32.83 | 1,907.13 | 0.00 | 0.00 |
| Jiangxi | Nancheng | 1,615.49 | 98.49 | 0.00 | 0.00 |
| Jiangxi | Nanfeng | 1,946.07 | 3.05 | 0.00 | 0.00 |
| Jiangxi | Nankang City | 1,830.02 | 0.00 | 0.00 | 0.00 |
| Jiangxi | Ningdu | 4,038.72 | 25.19 | 0.00 | 0.00 |
| Jiangxi | Ninggang | 588.63 | 8.40 | 0.00 | 0.00 |
| Jiangxi | Pengze | 687.12 | 917.68 | 0.00 | 0.00 |
| Jiangxi | Pingze District | 2,401.86 | 365.70 | 0.00 | 0.00 |
| Jiangxi | Qianshan | 1,729.24 | 474.11 | 0.00 | 0.00 |
| Jiangxi | Quannan | 1,497.92 | 802.40 | 649.71 | 6.11 |
| Jiangxi | Ruichang City | 0.00 | 802.40 | 649.71 | 6.11 |
| Jiangxi | Ruijin City | 2,420.94 | 0.00 | 0.00 | 0.00 |
| Jiangxi | Shanggao | 1,022.28 | 306.91 | 0.00 | 0.00 |
| Jiangxi | Shangrao City | 64.13 | 0.00 | 0.00 | 0.00 |
| Jiangxi | Shangrao | 1,528.45 | 0.00 | 0.00 | 0.00 |
| Jiangxi | Shicheng | 1,569.68 | 0.00 | 0.00 | 0.00 |
| Jiangxi | Suichuan | 2,947.73 | 103.83 | 0.00 | 0.00 |
| Jiangxi | Taihe | 2,127.01 | 556.56 | 0.00 | 0.00 |
| Jiangxi | Tonggu | 1,577.32 | 0.00 | 0.00 | 0.00 |
| Jiangxi | Wanan | 1,402.48 | 641.31 | 0.00 | 0.00 |
| Jiangxi | Wannian | 1,165.81 | 1.53 | 0.00 | 0.00 |
| Jiangxi | Wanzai | 1,708.63 | 0.00 | 0.00 | 0.00 |
| Jiangxi | Wuning | 1,893.39 | 1,622.36 | 71.00 | 0.00 |
| Jiangxi | Ziyuan | 2,115.56 | 892.49 | 0.00 | 0.00 |
| Jiangxi | Xiajiang | 429.83 | 873.40 | 0.00 | 0.00 |
| Jiangxi | Xingan | 283.24 | 961.20 | 1.53 | 0.00 |
| Jiangxi | Xinjian | 265.69 | 2,143.81 | 71.00 | 0.00 |
| Jiangxi | Xinyu District | 955.86 | 872.64 | 0.00 | 0.00 |
| Jiangxi | Xinfeng | 2,854.59 | 0.00 | 0.00 | 0.00 |
| Jiangxi | Xingzi | 346.61 | 323.71 | 64.13 | 0.00 |
| Jiangxi | Xingguo | 3,196.62 | 6.87 | 0.00 | 0.00 |
| Jiangxi | Xiushui | 4,305.17 | 284.01 | 0.00 | 0.00 |
| Jiangxi | Xunwu | 2,285.81 | 0.00 | 0.00 | 0.00 |
| Jiangxi | Yichun City | 2,507.21 | 61.08 | 0.00 | 0.00 |
| Jiangxi | Yifeng | 1,862.85 | 103.07 | 0.00 | 0.00 |
| Jiangxi | yihuang | 1,308.58 | 638.26 | 0.00 | 0.00 |
| Jiangxi | Yiyang | 1,562.81 | 15.27 | 0.00 | 0.00 |
| Jiangxi | Yingtan District | 123.68 | 0.00 | 0.00 | 0.00 |
| Jiangxi | Yongfeng | 2,425.52 | 301.57 | 0.00 | 0.00 |
| Jiangxi | Yongxin | 2,183.51 | 6.11 | 0.00 | 0.00 |
| Jiangxi | Yongxiu | 270.27 | 1,307.81 | 462.66 | 13.74 |
| Jiangxi | Yudu | 2,829.40 | 82.45 | 0.00 | 0.00 |
| Jiangxi | Yugan | 2,159.07 | 213.01 | 0.00 | 0.00 |
| Jiangxi | Yujiang | 942.11 | 0.00 | 0.00 | 0.00 |
| Jiangxi | Yushan | 1,788.80 | 0.00 | 0.00 | 0.00 |
| Jiangxi | Zhangshu City | 352.72 | 942.11 | 6.11 | 0.00 |
| Jiangxi | Zixi | 1,240.63 | 26.72 | 0.00 | 0.00 |
| Jilin | Dongfeng | 2,476.97 | 14.24 | 0.00 | 0.00 |
| Jilin | Helong District | 4,733.54 | 133.10 | 0.00 | 0.00 |
| Jilin | Huichun District | 2,749.98 | 2,532.68 | 0.00 | 0.00 |
| Jilin | Ji'an District | 3,151.77 | 5.57 | 0.00 | 0.00 |
| Jilin | Lishu | 4,038.92 | 112.05 | 0.00 | 0.00 |
| Jilin | Liaoyuan District | 151.68 | 78.62 | 0.00 | 0.00 |
| Jilin | Liu He | 3,225.44 | 22.91 | 0.00 | 0.00 |
| Jilin | Long Jing | 2,535.78 | 587.51 | 0.00 | 0.00 |
| Jilin | Tonghua District | 1,482.09 | 1.86 | 0.00 | 0.00 |
| Jilin | Tonghua | 2,805.70 | 2.48 | 0.00 | 0.00 |
| Jilin | Tumen District | 518.79 | 6.19 | 0.00 | 0.00 |
| Jilin | Yanji | 947.20 | 34.67 | 0.00 | 0.00 |
| Jilin | Antu | 7,335.56 | 0.00 | 0.00 | 0.00 |
| Jilin | Baicheng City | 997.35 | 0.00 | 0.00 | 0.00 |
| Jilin | Baishan District | 5,529.06 | 0.00 | 0.00 | 0.00 |
| Jilin | Daan District | 5,023.27 | 0.00 | 0.00 | 0.00 |
| Jilin | Dehui District | 3,517.65 | 0.00 | 0.00 | 0.00 |
| Jilin | Dunhua District | 11,708.79 | 0.00 | 0.00 | 0.00 |
| Jilin | Fuyu | 5,755.65 | 0.00 | 0.00 | 0.00 |
| Jilin | Fusong | 6,006.38 | 0.00 | 0.00 | 0.00 |
| Jilin | Gongzhuling District | 4,149.12 | 0.00 | 0.00 | 0.00 |
| Jilin | Huadian District | 6,393.93 | 0.00 | 0.00 | 0.00 |
| Jilin | Huinan | 2,233.66 | 0.00 | 0.00 | 0.00 |
| Jilin | Jilin District | 1,170.69 | 0.00 | 0.00 | 0.00 |
| Jilin | Jiaohe District | 10,894.07 | 0.00 | 0.00 | 0.00 |
| Jilin | Jingyu | 2,987.71 | 0.00 | 0.00 | 0.00 |
| Jilin | Jiutai District | 3,418.60 | 0.00 | 0.00 | 0.00 |
| Jilin | Meihekou V | 2,132.75 | 0.00 | 0.00 | 0.00 |
| Jilin | Nongan | 5,352.62 | 0.00 | 0.00 | 0.00 |
| Jilin | Qinshi District | 3,769.62 | 0.00 | 0.00 | 0.00 |
| Jilin | Qian Guo Luosi Mongolian Autonomous County | 6,408.17 | 0.00 | 0.00 | 0.00 |
| Jilin | Qianan | 3,591.94 | 0.00 | 0.00 | 0.00 |
| Jilin | Shuangliao District | 3,044.05 | 0.00 | 0.00 | 0.00 |
| Jilin | Shuangyang District | 2,054.75 | 0.00 | 0.00 | 0.00 |
| Jilin | Siping District | 416.65 | 0.00 | 0.00 | 0.00 |
| Jilin | Yaonan District | 6,189.63 | 0.00 | 0.00 | 0.00 |
| Jilin | Tongyu | 8,570.02 | 0.00 | 0.00 | 0.00 |
| Jilin | Wangqing | 8,910.51 | 0.00 | 0.00 | 0.00 |
| Jilin | Yitong Manchu Autonomous County | 2,496.78 | 0.00 | 0.00 | 0.00 |
| Jilin | Yongji | 5,090.13 | 0.00 | 0.00 | 0.00 |
| Jilin | Yushu District | 4,770.06 | 0.00 | 0.00 | 0.00 |
| Jilin | Changbai Korean Autonomous County | 2,358.10 | 0.00 | 0.00 | 0.00 |
| Jilin | Changchun District | 1,522.95 | 0.00 | 0.00 | 0.00 |
| Jilin | Changling | 5,732.74 | 0.00 | 0.00 | 0.00 |
| Jilin | Zhenglai | 5,517.30 | 0.00 | 0.00 | 0.00 |
| Liaoning | Anshan District | 0.00 | 439.21 | 257.05 | 0.00 |
| Liaoning | Beining City | 7.92 | 1,895.80 | 0.00 | 0.00 |
| Liaoning | Beipiao City | 172.08 | 4,899.71 | 0.00 | 0.00 |
| Liaoning | Benxi Manchu Autonomous County | 1,180.11 | 2,553.90 | 0.00 | 0.00 |
| Liaoning | Benxi District | 66.96 | 1,640.92 | 0.00 | 0.00 |
| Liaoning | Changtu | 2,840.46 | 2,080.13 | 0.00 | 0.00 |
| Liaoning | Chaoyang District | 64.80 | 514.81 | 0.00 | 0.00 |
| Liaoning | Chaoyang | 452.17 | 4,285.53 | 0.00 | 0.00 |
| Liaoning | Dalian District | 0.00 | 85.68 | 712.10 | 689.78 |
| Liaoning | Daqiao City | 0.00 | 988.58 | 755.30 | 0.00 |
| Liaoning | Dawa | 0.00 | 1,286.67 | 0.00 | 0.00 |
| Liaoning | Dandong District | 43.92 | 535.69 | 1.44 | 0.00 |
| Liaoning | Dengta City | 0.00 | 1,452.99 | 0.00 | 0.00 |
| Liaoning | Donggang city | 0.00 | 691.94 | 401.77 | 0.00 |
| Liaoning | Faku | 241.21 | 2,342.21 | 0.00 | 0.00 |
| Liaoning | Fengcheng City | 321.85 | 5,378.52 | 658.09 | 0.00 |
| Liaoning | Fushun District | 7.20 | 737.30 | 0.00 | 0.00 |
| Liaoning | Fushun | 98.64 | 2432.93 | 0.00 | 0.00 |
| Liaoning | Fuxin District | 2.16 | 435.61 | 0.00 | 0.00 |
| Liaoning | Gaizhou | 0.00 | 660.25 | 2,699.34 | 2.88 |
| Liaoning | Haicheng City | 0.00 | 1,686.28 | 1,331.31 | 0.00 |
| Liaoning | Gheishan | 218.88 | 2,552.46 | 0.00 | 0.00 |
| Liaoning | Huludao District | 155.52 | 2,153.57 | 0.00 | 0.00 |
| Liaoning | Huanren Manchu Autonomous County | 2,553.18 | 1,416.27 | 0.00 | 0.00 |
| Liaoning | Jianchang | 0.00 | 3,532.40 | 0.00 | 0.00 |
| Liaoning | Jianping | 3,165.19 | 2,330.69 | 0.00 | 0.00 |
| Liaoning | Jinzhou District | 2.88 | 588.25 | 0.00 | 0.00 |
| Liaoning | The Mongolian Autonomous County of Karakin | 139.68 | 2,354.45 | 0.00 | 0.00 |
| Liaoning | Kaiyuan City | 95.76 | 3,641.84 | 0.00 | 0.00 |
| Liaoning | Kangping | 1,836.76 | 658.09 | 0.00 | 0.00 |
| Liaoning | Kuandian Manchu Autonomous County | 3,696.56 | 3,186.79 | 0.00 | 0.00 |
| Liaoning | Liaoyang City | 0.00 | 638.65 | 2.88 | 0.00 |
| Liaoning | Liaoyang | 0.72 | 2,828.94 | 278.65 | 0.00 |
| Liaoning | Liaozhong | 0.00 | 1,834.60 | 0.00 | 0.00 |
| Liaoning | Linghai City | 206.64 | 2,260.85 | 0.00 | 0.00 |
| Liaoning | Lingyuan City | 0.00 | 3,648.32 | 0.00 | 0.00 |
| Liaoning | Panjin District | 0.00 | 180.72 | 0.00 | 0.00 |
| Liaoning | Panshan | 151.92 | 1,776.28 | 0.00 | 0.00 |
| Liaoning | Pulandian City | 0.00 | 262.09 | 1,949.80 | 784.10 |
| Liaoning | Qingyuan Manchu Autonomous County | 2,116.85 | 2,313.41 | 0.00 | 0.00 |
| Liaoning | Shenyang District | 3,842.73 | 0.00 | 0.00 | 0.00 |
| Liaoning | Suizhong | 409.69 | 2,611.50 | 0.00 | 0.00 |
| Liaoning | Taian | 0.72 | 1,552.35 | 0.00 | 0.00 |
| Liaoning | Tiefa City | 0.00 | 262.09 | 0.00 | 0.00 |
| Liaoning | Tieling District | 0.00 | 191.52 | 0.00 | 0.00 |
| Liaoning | Wafangdian City | 0.00 | 771.86 | 1,613.56 | 883.46 |
| Liaoning | Xifeng | 1,373.07 | 1,694.20 | 0.00 | 0.00 |
| Liaoning | New Bin Manchu Autonomous County | 3,722.48 | 1,232.67 | 0.00 | 0.00 |
| Liaoning | Xinming City | 25.20 | 3,704.48 | 0.00 | 0.00 |
| Liaoning | Xingcheng City | 257.05 | 1,845.40 | 0.00 | 0.00 |
| Liaoning | Xiuyan Manchu Autonomous County | 0.00 | 2,370.29 | 2,513.58 | 0.00 |
| Liaoning | Yi | 0.00 | 2,764.86 | 0.00 | 0.00 |
| Liaoning | Yingkou District | 0.00 | 558.01 | 0.00 | 0.00 |
| Liaoning | Zhangwu | 2,838.30 | 1,312.59 | 0.00 | 0.00 |
| Liaoning | Changhai | 0.00 | 8.64 | 33.84 | 12.24 |
| Liaoning | Zhuanghe City | 0.00 | 1,046.90 | 2,637.42 | 361.45 |
| Ningxia | Guyuan | 734.90 | 4,062.07 | 0.00 | 0.00 |
| Ningxia | Haiyuan | 5,987.69 | 985.12 | 0.00 | 0.00 |
| Ningxia | Helan | 1,611.53 | 0.00 | 0.00 | 0.00 |
| Ningxia | Huinong | 1,291.32 | 0.00 | 0.00 | 0.00 |
| Ningxia | Jingyuan | 0.00 | 906.38 | 65.62 | 0.00 |
| Ningxia | Linwu City | 4,748.86 | 0.00 | 0.00 | 0.00 |
| Ningxia | Longde | 0.00 | 1,251.08 | 0.00 | 0.00 |
| Ningxia | Pengyang | 0.00 | 3,264.18 | 0.00 | 0.00 |
| Ningxia | Pingluo | 2,678.89 | 0.00 | 0.00 | 0.00 |
| Ningxia | Qingtongxia | 2,427.80 | 0.00 | 0.00 | 0.00 |
| Ningxia | Shizuishan City | 683.28 | 0.00 | 0.00 | 0.00 |
| Ningxia | Taole | 1,110.22 | 0.00 | 0.00 | 0.00 |
| Ningxia | Tongxin | 8,972.78 | 5.25 | 0.00 | 0.00 |
| Ningxia | Wuzhong District | 1,265.95 | 0.00 | 0.00 | 0.00 |
| Ningxia | Xiji | 388.45 | 3,536.27 | 0.00 | 0.00 |
| Ningxia | Yanchi | 8,700.70 | 0.00 | 0.00 | 0.00 |
| Ningxia | Yinchuan District | 1,756.76 | 0.00 | 0.00 | 0.00 |
| Ningxia | Yongning | 1,237.96 | 0.00 | 0.00 | 0.00 |
| Ningxia | Zhongning | 2,713.01 | 0.00 | 0.00 | 0.00 |
| Ningxia | Zhongwei | 6,013.93 | 0.00 | 0.00 | 0.00 |
| Qinghai | Banma | 6,266.82 | 0.00 | 0.00 | 0.00 |
| Qinghai | Chenduo | 14,588.79 | 0.00 | 0.00 | 0.00 |
| Qinghai | Dari | 14,435.31 | 0.00 | 0.00 | 0.00 |
| Qinghai | Datong Hui and Tu Autonomous County | 3,075.33 | 0.00 | 0.00 | 0.00 |
| Qinghai | Delingha City | 27,091.46 | 0.00 | 0.00 | 0.00 |
| Qinghai | Dulan | 54,858.81 | 0.00 | 0.00 | 0.00 |
| Qinghai | Gande | 7,159.57 | 0.00 | 0.00 | 0.00 |
| Qinghai | Gangcha | 11,207.90 | 0.00 | 0.00 | 0.00 |
| Qinghai | Germu City | 123,621.36 | 0.00 | 0.00 | 0.00 |
| Qinghai | Gonghe | 15,425.21 | 0.00 | 0.00 | 0.00 |
| Qinghai | Guide | 3,267.53 | 0.00 | 0.00 | 0.00 |
| Qinghai | Guinan | 6,561.82 | 0.00 | 0.00 | 0.00 |
| Qinghai | Haixi Mongolian and Tibetan Autonomous Prefecture | 95,801.21 | 0.00 | 0.00 | 0.00 |
| Qinghai | Haiyan | 4,756.62 | 0.00 | 0.00 | 0.00 |
| Qinghai | Henan Mongolian Autonomous County | 6,492.12 | 0.00 | 0.00 | 0.00 |
| Qinghai | Huzhu Tu autonomous county | 3,328.79 | 0.00 | 0.00 | 0.00 |
| Qinghai | Hualong Hui Autonomous County | 2,864.11 | 0.00 | 0.00 | 0.00 |
| Qinghai | Huangyuan | 1,525.69 | 0.00 | 0.00 | 0.00 |
| Qinghai | Huangzhong | 2,457.87 | 0.00 | 0.00 | 0.00 |
| Qinghai | Jianzha | 1,605.96 | 0.00 | 0.00 | 0.00 |
| Qinghai | Jiuzhi | 8,236.07 | 0.00 | 0.00 | 0.00 |
| Qinghai | Ledu | 2,714.85 | 0.00 | 0.00 | 0.00 |
| Qinghai | Maduo | 26,457.10 | 0.00 | 0.00 | 0.00 |
| Qinghai | Maqin | 13,505.25 | 0.00 | 0.00 | 0.00 |
| Qinghai | Menyuan Hui Autonomous County | 5,685.27 | 0.00 | 0.00 | 0.00 |
| Qinghai | Minghe Hui Autonomous County | 1,884.76 | 0.00 | 0.00 | 0.00 |
| Qinghai | Rangqian | 11,865.49 | 0.00 | 0.00 | 0.00 |
| Qinghai | Pingan | 729.40 | 0.00 | 0.00 | 0.00 |
| Qinghai | Qilian | 13,715.05 | 0.00 | 0.00 | 0.00 |
| Qinghai | Qumalai | 38,781.64 | 0.00 | 0.00 | 0.00 |
| Qinghai | Tianjun | 24,475.88 | 0.00 | 0.00 | 0.00 |
| Qinghai | Tongde | 4,648.90 | 0.00 | 0.00 | 0.00 |
| Qinghai | Tingren | 3,206.99 | 0.00 | 0.00 | 0.00 |
| Qinghai | Wulan | 7,393.32 | 0.00 | 0.00 | 0.00 |
| Qinghai | Xining District | 331.61 | 0.00 | 0.00 | 0.00 |
| Qinghai | Xinghai | 1,1852.12 | 0.00 | 0.00 | 0.00 |
| Qinghai | Xunhua Salar Autonomous County | 1,729.17 | 0.00 | 0.00 | 0.00 |
| Qinghai | Yushu | 14,860.56 | 0.00 | 0.00 | 0.00 |
| Qinghai | Zaduo | 35,341.61 | 0.00 | 0.00 | 0.00 |
| Qinghai | Duoku | 6,525.21 | 0.00 | 0.00 | 0.00 |
| Qinghai | Zhiduo | 80,555.53 | 0.00 | 0.00 | 0.00 |
| Shaanxi | Ankang City | 0.00 | 835.31 | 2,459.37 | 230.70 |
| Shaanxi | Ansai | 0.00 | 3,020.95 | 1.41 | 0.00 |
| Shaanxi | Baihe | 0.00 | 660.35 | 690.68 | 48.68 |
| Shaanxi | Baishui | 0.00 | 0.00 | 4.94 | 965.83 |
| Shaanxi | Baoji District | 0.00 | 0.00 | 134.05 | 390.85 |
| Shaanxi | Baoji | 0.00 | 0.00 | 405.66 | 2,670.32 |
| Shaanxi | Bin | 0.00 | 0.00 | 71.26 | 1,131.62 |
| Shaanxi | Chenggu | 0.00 | 33.16 | 2,101.68 | 16.93 |
| Shaanxi | Chengcheng | 0.00 | 0.00 | 67.73 | 1,063.19 |
| Shaanxi | Chunhua | 0.00 | 0.00 | 0.00 | 972.18 |
| Shaanxi | Dali | 0.00 | 0.00 | 1,741.88 | 75.49 |
| Shaanxi | Danfeng | 0.00 | 0.00 | 178.49 | 2,200.45 |
| Shaanxi | Dingbian | 5,969.94 | 1,111.16 | 0.00 | 0.00 |
| Shaanxi | Feng | 0.00 | 0.00 | 924.91 | 2,177.17 |
| Shaanxi | Fengxiang | 0.00 | 0.00 | 0.00 | 1,250.15 |
| Shaanxi | Foping | 0.00 | 8.47 | 1,030.03 | 215.18 |
| Shaanxi | Fufeng | 0.00 | 0.00 | 0.00 | 747.12 |
| Shaanxi | Fugu | 0.00 | 2,386.71 | 1,008.87 | 0.00 |
| Shaanxi | Fuping | 0.00 | 0.00 | 63.50 | 1,177.48 |
| Shaanxi | Fu | 0.00 | 152.39 | 3,447.78 | 562.28 |
| Shaanxi | Ganquan | 0.00 | 934.79 | 1,413.12 | 0.00 |
| Shaanxi | Gaoling | 0.00 | 0.00 | 0.00 | 294.90 |
| Shaanxi | Hancheng | 0.00 | 0.00 | 28.93 | 1,583.85 |
| Shaanxi | Hanyin | 0.00 | 273.73 | 923.50 | 112.17 |
| Shaanxi | Hanzhong | 0.00 | 31.75 | 515.72 | 0.00 |
| Shaanxi | Heyang | 0.00 | 0.00 | 161.56 | 1,190.18 |
| Shaanxi | Hengshan | 0.00 | 4,278.86 | 138.28 | 0.00 |
| Shaanxi | Hu | 0.00 | 0.00 | 26.10 | 1,247.32 |
| Shaanxi | Hua | 0.00 | 0.00 | 278.67 | 862.83 |
| Shaanxi | Huayin City | 0.00 | 0.00 | 331.59 | 335.82 |
| Shaanxi | Huangling | 0.00 | 0.00 | 51.50 | 2,237.14 |
| Shaanxi | Huanglong | 0.00 | 0.00 | 150.98 | 2,620.93 |
| Shaanxi | Jia | 0.00 | 1,066.01 | 1,078.71 | 0.00 |
| Shaanxi | Jingyang | 0.00 | 0.00 | 0.00 | 789.45 |
| Shaanxi | Jingbian | 130.52 | 5,009.05 | 0.00 | 0.00 |
| Shaanxi | Langao | 0.00 | 34.57 | 1,658.63 | 212.36 |
| Shaanxi | Lantian | 0.00 | 0.00 | 1.41 | 2,002.91 |
| Shaanxi | Liquan | 0.00 | 0.00 | 0.00 | 1,012.39 |
| Shaanxi | Lintong District | 0.00 | 0.00 | 304.78 | 804.98 |
| Shaanxi | Linyou | 0.00 | 0.00 | 0.00 | 1,690.38 |
| Shaanxi | Liuba | 0.00 | 23.99 | 1,407.47 | 526.30 |
| Shaanxi | Long | 0.00 | 0.00 | 1,461.09 | 840.25 |
| Shaanxi | Luochuan | 0.00 | 0.00 | 289.96 | 1,534.46 |
| Shaanxi | Luonan | 0.00 | 0.00 | 0.00 | 2,826.23 |
| Shaanxi | Lueyang | 0.00 | 239.87 | 2,389.53 | 133.34 |
| Shaanxi | Mei | 0.00 | 14.82 | 56.44 | 757.71 |
| Shaanxi | Mizhi | 0.00 | 1,044.14 | 155.21 | 0.00 |
| Shaanxi | Mian | 0.00 | 64.20 | 2,056.53 | 206.71 |
| Shaanxi | Nanzhi | 0.00 | 589.09 | 2,209.63 | 0.00 |
| Shaanxi | Ningqiang | 16.93 | 1,827.25 | 1,358.09 | 0.00 |
| Shaanxi | Ningshan | 0.00 | 0.00 | 985.58 | 2,604.00 |
| Shaanxi | Pingli | 0.00 | 318.18 | 1,638.17 | 617.31 |
| Shaanxi | Pucheng | 0.00 | 0.00 | 841.66 | 747.12 |
| Shaanxi | Qishan | 0.00 | 0.00 | 0.00 | 839.55 |
| Shaanxi | Qianyang | 0.00 | 0.00 | 0.00 | 1,017.33 |
| Shaanxi | Qian | 0.00 | 0.00 | 0.00 | 998.99 |
| Shaanxi | Qingjian | 0.00 | 1,925.31 | 0.00 | 0.00 |
| Shaanxi | Sanyuan | 0.00 | 0.00 | 0.00 | 553.82 |
| Shaanxi | Shanyang | 0.00 | 143.22 | 1,125.98 | 2,213.15 |
| Shaanxi | Shangnan | 0.00 | 0.00 | 1,061.07 | 1,214.87 |
| Shaanxi | Shangzhou | 0.00 | 0.00 | 0.00 | 2,636.45 |
| Shaanxi | Shenmu | 0.00 | 7,560.84 | 305.48 | 0.00 |
| Shaanxi | Shiquan | 0.00 | 75.49 | 0.00 | 0.00 |
| Shaanxi | Suide | 0.00 | 1,909.08 | 0.00 | 0.00 |
| Shaanxi | Taibai | 658.94 | 532.65 | 635.66 | 874.11 |
| Shaanxi | Tongchuan District | 0.00 | 0.00 | 0.00 | 804.98 |
| Shaanxi | Tongguan | 0.00 | 0.00 | 71.96 | 351.34 |
| Shaanxi | Weinan | 0.00 | 0.00 | 771.11 | 483.97 |
| Shaanxi | Wubao | 0.00 | 440.23 | 0.00 | 0.00 |
| Shaanxi | Wuqi | 884.70 | 3,020.25 | 0.00 | 0.00 |
| Shaanxi | Wugong | 0.00 | 0.00 | 0.00 | 286.43 |
| Shaanxi | Xian District | 0.00 | 0.00 | 277.26 | 625.07 |
| Shaanxi | Xixiang | 0.00 | 229.99 | 2,941.23 | 0.00 |
| Shaanxi | Xianyang District | 0.00 | 0.00 | 94.54 | 635.66 |
| Shaanxi | Xingping City | 0.00 | 0.00 | 0.00 | 493.85 |
| Shaanxi | Xunyang | 0.00 | 1,228.98 | 2,005.03 | 249.75 |
| Shaanxi | Xunyi | 0.00 | 0.00 | 44.45 | 1,748.23 |
| Shaanxi | Yanan City | 0.00 | 2,688.66 | 926.32 | 0.00 |
| Shaanxi | Yanchuan | 0.00 | 2,024.08 | 0.00 | 0.00 |
| Shaanxi | Yanchang | 0.00 | 2,238.55 | 191.19 | 0.00 |
| Shaanxi | Yang | 0.00 | 10.58 | 3,138.06 | 11.29 |
| Shaanxi | Yao | 0.00 | 1,608.54 | 0.00 | 0.00 |
| Shaanxi | Yichuan | 0.00 | 54.32 | 1,872.40 | 1,058.96 |
| Shaanxi | Yijun | 0.00 | 0.00 | 0.00 | 1,533.76 |
| Shaanxi | Yongshou | 0.00 | 0.00 | 0.00 | 869.88 |
| Shaanxi | Yulin City | 826.85 | 6,113.86 | 366.86 | 0.00 |
| Shaanxi | Zhashui | 0.00 | 5.64 | 256.10 | 2,050.18 |
| Shaanxi | Changan | 0.00 | 0.00 | 17.64 | 1,575.38 |
| Shaanxi | Changwu | 0.00 | 0.00 | 138.28 | 426.83 |
| Shaanxi | Zhenan | 0.00 | 26.81 | 1,703.08 | 1,734.82 |
| Shaanxi | Zhenba | 0.00 | 1,988.10 | 1,315.05 | 0.00 |
| Shaanxi | Zhengping | 0.00 | 0.71 | 1,130.92 | 323.82 |
| Shaanxi | Zhidan | 0.00 | 3,849.21 | 19.75 | 0.00 |
| Shaanxi | Zhouzhi | 99.48 | 84.66 | 717.49 | 2,061.47 |
| Shaanxi | Zichang | 0.00 | 2,473.48 | 0.00 | 0.00 |
| Shaanxi | Zizhou | 0.00 | 2,111.56 | 0.00 | 0.00 |
| Shaanxi | Ankang City | 0.00 | 1,249.44 | 927.03 | 0.00 |
| Shandong | Anqiu City | 0.00 | 352.56 | 1,792.57 | 8.18 |
| Shandong | Binzhou City | 3.72 | 1,051.74 | 0.00 | 0.00 |
| Shandong | Boxing | 0.00 | 966.20 | 0.00 | 0.00 |
| Shandong | Cangshan | 0.00 | 0.00 | 1,865.46 | 54.30 |
| Shandong | Cao | 0.00 | 0.00 | 1,125.37 | 948.35 |
| Shandong | Changle | 0.00 | 43.88 | 1,026.45 | 39.42 |
| Shandong | Changyi | 0.00 | 1,430.33 | 15.62 | 0.00 |
| Shandong | Chengwu | 0.00 | 0.00 | 1,080.00 | 0.00 |
| Shandong | Chaping | 0.00 | 1,216.12 | 0.00 | 0.00 |
| Shandong | Dan | 0.00 | 0.00 | 1,727.11 | 5.95 |
| Shandong | Dezhou City | 26.03 | 275.21 | 0.00 | 0.00 |
| Shandong | Dingtao | 0.00 | 0.00 | 701.41 | 167.36 |
| Shandong | Donga | 0.00 | 676.86 | 182.23 | 0.00 |
| Shandong | Dongming | 0.00 | 0.00 | 1,312.81 | 65.45 |
| Shandong | Dongping | 0.00 | 0.00 | 1,322.48 | 45.37 |
| Shandong | Dongying District | 968.43 | 1,571.66 | 0.00 | 0.00 |
| Shandong | Feicheng City | 0.00 | 0.00 | 1,346.28 | 0.00 |
| Shandong | Fei | 0.00 | 0.00 | 2,028.35 | 3.72 |
| Shandong | Gaomi City | 0.00 | 1,132.07 | 624.05 | 19.34 |
| Shandong | Gaoqing | 0.00 | 990.75 | 0.00 | 0.00 |
| Shandong | Gaotang | 0.00 | 1,027.19 | 0.00 | 0.00 |
| Shandong | Guan | 0.00 | 1,239.18 | 0.00 | 0.00 |
| Shandong | Guangrao | 0.00 | 1,193.06 | 0.00 | 0.00 |
| Shandong | Haiyang City | 0.00 | 691.74 | 1,259.26 | 0.74 |
| Shandong | Heze City | 0.00 | 0.00 | 1,163.31 | 356.28 |
| Shandong | Yuantai | 0.00 | 0.00 | 556.36 | 0.00 |
| Shandong | Huiming | 0.00 | 0.00 | 1,509.92 | 0.00 |
| Shandong | Jimo City | 0.00 | 0.00 | 299.01 | 2,958.10 |
| Shandong | Jinan District | 0.00 | 517.69 | 557.11 | 955.79 |
| Shandong | Jining District | 0.00 | 182.23 | 806.28 | 0.00 |
| Shandong | Jiyang | 0.00 | 1,296.45 | 0.00 | 0.00 |
| Shandong | Jiaxiang | 0.00 | 0.00 | 1,070.33 | 20.08 |
| Shandong | Jiaonan City | 0.00 | 270.00 | 803.31 | 670.17 |
| Shandong | Jiaozhou City | 0.00 | 2.23 | 1,036.86 | 351.82 |
| Shandong | Jinxiang | 0.00 | 0.00 | 930.50 | 0.00 |
| Shandong | Junan | 0.00 | 756.45 | 1,110.50 | 0.00 |
| Shandong | Ju | 0.00 | 1,445.95 | 612.15 | 0.00 |
| Shandong | Juye | 0.00 | 0.00 | 1,337.36 | 0.00 |
| Shandong | Yecheng | 0.00 | 0.00 | 1,122.40 | 0.00 |
| Shandong | Kenli | 453.72 | 894.80 | 0.00 | 0.00 |
| Shandong | Laiwu | 0.00 | 0.00 | 1,578.35 | 659.01 |
| Shandong | Laixi | 0.00 | 0.00 | 1,714.47 | 2.23 |
| Shandong | Laiyang | 0.00 | 557.85 | 1,267.44 | 0.00 |
| Shandong | Laizhou | 69.92 | 393.47 | 534.05 | 0.00 |
| Shandong | Leling City | 2.23 | 1,245.13 | 0.00 | 0.00 |
| Shandong | Lijin | 169.59 | 1,133.56 | 0.00 | 0.00 |
| Shandong | Liangshan | 0.00 | 0.00 | 975.13 | 1.49 |
| Shandong | Laiocheng District | 0.00 | 1,347.03 | 0.00 | 0.00 |
| Shandong | Linqing City | 0.00 | 1,043.56 | 0.00 | 0.00 |
| Shandong | Linqu | 0.00 | 0.00 | 1,699.59 | 336.94 |
| Shandong | Linshu | 0.00 | 8.93 | 1,071.08 | 20.08 |
| Shandong | Linyi | 0.00 | 42.40 | 1,782.15 | 20.83 |
| Shandong | Linyii | 27.52 | 1,085.95 | 0.00 | 0.00 |
| Shandong | Ling | 412.07 | 1,136.53 | 0.00 | 0.00 |
| Shandong | Longkou City | 84.79 | 251.41 | 185.21 | 0.74 |
| Shandong | Mengyin | 0.00 | 0.00 | 1,689.92 | 2.23 |
| Shandong | Muping | 0.00 | 673.14 | 1,160.33 | 27.52 |
| Shandong | Ningjin | 272.98 | 609.92 | 0.00 | 0.00 |
| Shandong | Ningyang | 0.00 | 23.06 | 1,163.31 | 0.00 |
| Shandong | Penglai City | 126.45 | 744.55 | 231.32 | 0.00 |
| Shandong | Pingdu City | 0.00 | 2,099.01 | 1,298.68 | 0.74 |
| Shandong | Pingyi | 0.00 | 0.00 | 1,901.90 | 27.52 |
| Shandong | Pingyin | 0.00 | 150.25 | 583.89 | 111.57 |
| Shandong | Pingyuan | 0.00 | 1,208.68 | 0.00 | 0.00 |
| Shandong | Xixia City | 0.00 | 103.39 | 2,079.67 | 0.00 |
| Shandong | Qihe | 0.00 | 1,746.45 | 0.00 | 0.00 |
| Shandong | Qingdao District | 0.00 | 0.00 | 496.86 | 169.59 |
| Shandong | Qingzhou City | 0.00 | 179.26 | 717.03 | 775.79 |
| Shandong | Qingyun | 0.00 | 556.36 | 0.00 | 0.00 |
| Shandong | Qufu | 0.00 | 52.07 | 821.16 | 0.00 |
| Shandong | Rizhao City | 0.00 | 752.73 | 1,064.38 | 14.13 |
| Shandong | Rongcheng City | 0.00 | 334.71 | 587.60 | 79.59 |
| Shandong | Rushan City | 0.00 | 406.12 | 687.27 | 3.72 |
| Shandong | Shanghe | 0.00 | 1,220.58 | 0.00 | 0.00 |
| Shandong | Zi | 0.00 | 1,135.79 | 319.09 | 0.00 |
| Shandong | Shouguang City | 0.00 | 2,065.54 | 16.36 | 0.00 |
| Shandong | Sishui | 0.00 | 0.00 | 1,207.94 | 0.00 |
| Shandong | Taian District | 0.00 | 0.00 | 2,055.87 | 154.71 |
| Shandong | Tancheng | 0.00 | 22.31 | 1,335.13 | 20.08 |
| Shandong | Tezhou City | 0.00 | 2.23 | 1,552.32 | 2.98 |
| Shandong | Weihai District | 0.00 | 141.32 | 206.03 | 17.85 |
| Shandong | huishan | 0.00 | 194.88 | 1,527.77 | 9.67 |
| Shandong | Weifang District | 0.00 | 1,106.04 | 382.31 | 0.00 |
| Shandong | Wendeng | 0.00 | 885.87 | 957.27 | 26.03 |
| Shandong | Wenshang | 0.00 | 0.00 | 958.02 | 0.00 |
| Shandong | Wudi | 593.55 | 1,276.37 | 0.00 | 0.00 |
| Shandong | Wulian | 0.00 | 122.73 | 1,473.47 | 7.44 |
| Shandong | Wucheng | 3.72 | 803.31 | 0.00 | 0.00 |
| Shandong | Xiajin | 0.00 | 966.20 | 0.00 | 0.00 |
| Shandong | Xintai | 0.00 | 0.00 | 2,070.75 | 17.85 |
| Shandong | Yantai District | 0.00 | 303.47 | 257.36 | 0.00 |
| Shandong | Yunzhou | 0.00 | 434.38 | 243.97 | 0.00 |
| Shandong | Yanggu | 0.00 | 1020.50 | 46.12 | 0.00 |
| Shandong | Yangxin | 13.39 | 865.79 | 0.00 | 0.00 |
| Shandong | Yinan | 0.00 | 463.39 | 1,438.52 | 0.00 |
| Shandong | Yishui | 0.00 | 609.92 | 1,944.30 | 0.00 |
| Shandong | Yiyuan | 0.00 | 0.00 | 1,843.89 | 0.00 |
| Shandong | Yutai | 0.00 | 0.00 | 685.04 | 0.00 |
| Shandong | Yucheng | 0.00 | 1,055.46 | 0.00 | 0.00 |
| Shandong | Yuncheng | 0.00 | 1,754.63 | 0.00 | 0.00 |
| Shandong | Zaozhuang District | 0.00 | 49.09 | 3,024.30 | 194.13 |
| Shandong | Zhanhua | 746.78 | 841.99 | 0.00 | 0.00 |
| Shandong | Zhangqiu City | 0.00 | 598.76 | 278.18 | 987.03 |
| Shandong | Changdao | 24.55 | 0.74 | 0.00 | 0.00 |
| Shandong | Changqing | 0.00 | 328.02 | 951.32 | 18.60 |
| Shandong | Zhaoyuan City | 7.44 | 290.08 | 1,176.70 | 0.00 |
| Shandong | Zibo District | 0.00 | 376.36 | 1,360.42 | 1,487.61 |
| Shandong | Zhoucheng City | 0.00 | 277.44 | 1,445.21 | 5.21 |
| Shandong | Zhouping | 0.00 | 1,071.08 | 122.73 | 171.07 |
| Shanghai | Chongming | 0.00 | 1,080.70 | 0.00 | 0.00 |
| Shanghai | Fengxian | 0.00 | 311.15 | 0.00 | 0.00 |
| Shanghai | JIAding District | 0.00 | 548.25 | 0.00 | 0.00 |
| Shanghai | JInshanDistrict | 0.00 | 643.93 | 0.00 | 0.00 |
| Shanghai | Minxing District | 0.00 | 517.47 | 0.00 | 0.00 |
| Shanghai | Nanhui | 0.00 | 507.49 | 0.00 | 0.00 |
| Shanghai | Qingpu | 0.00 | 771.22 | 0.00 | 0.00 |
| Shanghai | Shanghai District | 0.00 | 1,249.59 | 0.00 | 0.00 |
| Shanghai | Songjiang District | 0.00 | 677.21 | 0.00 | 0.00 |
| Shanxi | Anze | 0.00 | 0.00 | 1,867.00 | 0.00 |
| Shanxi | Baode | 0.00 | 406.78 | 539.11 | 0.00 |
| Shanxi | Daning | 0.00 | 411.34 | 481.74 | 0.00 |
| Shanxi | Datong District | 1,119.16 | 938.84 | 0.00 | 0.00 |
| Shanxi | Datong | 503.52 | 962.57 | 0.00 | 0.00 |
| Shanxi | Dai | 629.77 | 1,033.18 | 0.00 | 0.00 |
| Shanxi | Dingxiang | 0.00 | 828.55 | 0.00 | 0.00 |
| Shanxi | Fanzhi | 1,246.93 | 1,112.24 | 0.00 | 0.00 |
| Shanxi | Fangshan | 383.13 | 1,002.13 | 0.00 | 0.00 |
| Shanxi | Fenxi | 0.00 | 0.00 | 780.31 | 27.38 |
| Shanxi | Fenyang City | 0.00 | 11.08 | 1,123.20 | 0.00 |
| Shanxi | Fushan | 0.00 | 0.00 | 0.00 | 850.06 |
| Shanxi | Gaoping City | 0.00 | 0.00 | 0.00 | 910.03 |
| Shanxi | Gujiao City | 0.00 | 678.61 | 782.91 | 0.00 |
| Shanxi | Gu | 0.00 | 0.00 | 78.88 | 1,009.77 |
| Shanxi | Guangling | 636.29 | 575.56 | 0.00 | 0.00 |
| Shanxi | Heshun | 0.00 | 354.63 | 1,747.70 | 0.00 |
| Shanxi | Hejin City | 0.00 | 0.00 | 1.30 | 553.45 |
| Shanxi | Heqi | 0.00 | 0.00 | 1,289.43 | 0.00 |
| Shanxi | Hongdong | 0.00 | 0.00 | 816.16 | 582.13 |
| Shanxi | Houma City | 0.00 | 0.00 | 207.95 | 0.00 |
| Shanxi | Huguan | 0.00 | 0.00 | 127.12 | 823.98 |
| Shanxi | Huanren | 6,862.15 | 395.20 | 0.00 | 0.00 |
| Shanxi | Hunyuan | 114.08 | 1,819.41 | 0.00 | 0.00 |
| Shanxi | Huozhou City | 0.00 | 4.56 | 506.51 | 213.82 |
| Shanxi | Ji | 0.00 | 46.94 | 1,247.71 | 361.80 |
| Shanxi | Jishan | 0.00 | 0.00 | 0.00 | 638.85 |
| Shanxi | Jiang | 0.00 | 0.00 | 0.00 | 961.53 |
| Shanxi | Jiaocheng | 2.61 | 859.84 | 904.16 | 0.00 |
| Shanxi | Jiaokou | 0.00 | 207.30 | 963.49 | 0.00 |
| Shanxi | Jiexiu city | 0.00 | 0.00 | 382.00 | 286.83 |
| Shanxi | Jincheng District | 0.00 | 0.00 | 0.00 | 2030.62 |
| Shanxi | Jingle | 26.08 | 1,946.53 | 0.00 | 0.00 |
| Shanxi | Kelan | 749.07 | 1,169.43 | 0.00 | 0.00 |
| Shanxi | Lan | 0.65 | 1,441.97 | 0.00 | 0.00 |
| Shanxi | Lishi City | 0.00 | 1,090.60 | 183.18 | 0.00 |
| Shanxi | Licheng | 0.00 | 5.22 | 913.94 | 144.07 |
| Shanxi | Linfen City | 0.00 | 0.00 | 449.80 | 784.22 |
| Shanxi | Lin | 0.00 | 2,284.86 | 568.44 | 0.00 |
| Shanxi | Linyi | 0.00 | 0.00 | 24.12 | 1,224.89 |
| Shanxi | Linqiu | 595.88 | 2,042.30 | 0.65 | 0.00 |
| Shanxi | Linshi | 0.00 | 20.21 | 825.94 | 295.96 |
| Shanxi | Lingchuan | 0.00 | 0.00 | 3.91 | 1,561.92 |
| Shanxi | Liulin | 0.00 | 0.00 | 1,235.32 | 0.00 |
| Shanxi | Loufan | 0.00 | 1,217.07 | 44.98 | 0.00 |
| Shanxi | Lucheng City | 0.00 | 0.00 | 466.10 | 0.00 |
| Shanxi | Ningwu | 824.24 | 1,074.05 | 0.00 | 0.00 |
| Shanxi | Pianguan | 506.12 | 1,131.41 | 0.00 | 0.00 |
| Shanxi | Pingding | 0.00 | 114.08 | 1,241.84 | 0.00 |
| Shanxi | Pinglu | 0.00 | 0.00 | 391.78 | 683.83 |
| Shanxi | Pingshun | 0.00 | 0.00 | 439.37 | 973.26 |
| Shanxi | Pingyao | 0.00 | 0.00 | 869.61 | 344.85 |
| Shanxi | Pu | 0.00 | 0.65 | 1,149.27 | 279.66 |
| Shanxi | Qi | 0.00 | 1.96 | 709.25 | 118.64 |
| Shanxi | Qinshui | 0.00 | 0.00 | 2,533.23 | 0.00 |
| Shanxi | Qin | 0.00 | 0.00 | 280.96 | 970.01 |
| Shanxi | Qinyuan | 0.00 | 20.21 | 1,308.33 | 1,123.20 |
| Shanxi | Qingxu | 0.00 | 159.71 | 427.64 | 0.00 |
| Shanxi | Quwo | 0.00 | 0.00 | 0.00 | 404.82 |
| Shanxi | Ruicheng | 0.00 | 0.00 | 367.66 | 719.68 |
| Shanxi | Shanyin | 819.71 | 811.96 | 0.00 | 0.00 |
| Shanxi | Shenchi | 211.45 | 1,255.95 | 0.00 | 0.00 |
| Shanxi | Shilou | 0.00 | 1,619.93 | 0.00 | 0.00 |
| Shanxi | Shouyang | 0.00 | 2,029.97 | 7.17 | 0.00 |
| Shanxi | Suzhou District | 1,570.26 | 2,434.27 | 0.00 | 0.00 |
| Shanxi | Taigu | 0.00 | 91.92 | 897.65 | 1.96 |
| Shanxi | Taiyuan District | 0.00 | 911.99 | 486.96 | 0.00 |
| Shanxi | Tianzheng | 693.19 | 906.54 | 0.00 | 0.00 |
| Shanxi | Tunliu | 0.00 | 0.00 | 348.76 | 756.19 |
| Shanxi | Wanrong | 0.00 | 0.00 | 0.65 | 971.96 |
| Shanxi | Wenshui | 0.00 | 85.40 | 844.84 | 0.00 |
| Shanxi | Wenxi | 0.00 | 0.00 | 0.00 | 1,090.60 |
| Shanxi | Wutai | 1,059.18 | 2,684.46 | 0.00 | 0.00 |
| Shanxi | Wuzhai | 432.88 | 928.26 | 0.00 | 0.00 |
| Shanxi | Wuxiang | 0.00 | 3.26 | 1,284.21 | 243.15 |
| Shanxi | Xiyang | 0.00 | 204.69 | 1,584.08 | 80.18 |
| Shanxi | Xi County | 0.00 | 508.47 | 855.93 | 0.00 |
| Shanxi | Xia | 0.00 | 0.00 | 184.48 | 1,080.83 |
| Shanxi | Xiangning | 0.00 | 0.00 | 34.55 | 1,852.66 |
| Shanxi | Xiangfen | 0.00 | 0.00 | 67.14 | 893.08 |
| Shanxi | Xiangyuan | 0.00 | 0.00 | 391.78 | 705.99 |
| Shanxi | Xiaoyi | 0.00 | 0.00 | 884.61 | 26.08 |
| Shanxi | Xinzhou City | 0.00 | 1,895.68 | 0.00 | 0.00 |
| Shanxi | Xinjiang | 0.00 | 0.00 | 0.00 | 552.15 |
| Shanxi | Xing | 0.00 | 2,067.78 | 996.73 | 0.00 |
| Shanxi | Yangcheng | 0.00 | 0.00 | 0.00 | 1,814.85 |
| Shanxi | Yanggao | 321.61 | 1,340.04 | 0.00 | 0.00 |
| Shanxi | Yangqu | 0.00 | 1,999.98 | 6.52 | 0.00 |
| Shanxi | Yangquan District | 0.00 | 496.74 | 140.16 | 0.00 |
| Shanxi | Yicheng | 0.00 | 0.00 | 3.26 | 1,036.50 |
| Shanxi | Ying | 601.09 | 1,041.66 | 0.00 | 0.00 |
| Shanxi | Yonghe | 0.00 | 0.00 | 1,138.19 | 0.00 |
| Shanxi | Yongji | 0.00 | 0.00 | 505.21 | 542.37 |
| Shanxi | Youyu | 970.01 | 1,026.72 | 0.00 | 0.00 |
| Shanxi | Yu | 0.00 | 2,274.43 | 134.94 | 0.00 |
| Shanxi | Yuci City | 0.00 | 1,086.04 | 166.88 | 0.00 |
| Shanxi | Yushe | 0.00 | 0.00 | 1,606.25 | 0.00 |
| Shanxi | Yuanqu | 0.00 | 0.00 | 0.65 | 1,516.28 |
| Shanxi | Yuanping | 994.65 | 1,466.87 | 0.00 | 0.00 |
| Shanxi | Yuncheng City | 0.00 | 0.00 | 171.45 | 916.55 |
| Shanxi | Changzhi District | 0.00 | 0.00 | 36.51 | 308.99 |
| Shanxi | Changzhi | 0.00 | 0.00 | 10.43 | 438.07 |
| Shanxi | Changzi | 0.00 | 0.00 | 4.56 | 959.58 |
| Shanxi | Zhongyang | 0.00 | 1,183.82 | 185.79 | 0.00 |
| Shanxi | Zuoquan | 0.00 | 42.37 | 1,872.87 | 5.87 |
| Shanxi | Zuoyun | 412.64 | 908.08 | 0.00 | 0.00 |
| Sichuan | Aba | 1,0184.63 | 0.00 | 0.00 | 0.00 |
| Sichuan | An | 0.00 | 112.88 | 1,083.09 | 213.22 |
| Sichuan | Anyue | 0.00 | 953.24 | 1,726.46 | 0.00 |
| Sichuan | Batang | 8,132.06 | 0.00 | 0.00 | 0.00 |
| Sichuan | Bazhong City | 0.00 | 445.63 | 2,096.10 | 50.17 |
| Sichuan | Baiyu | 10,437.69 | 0.00 | 0.00 | 0.00 |
| Sichuan | Baoxing | 2,564.60 | 222.08 | 320.94 | 0.00 |
| Sichuan | Beichuan | 37.63 | 506.13 | 2,355.07 | 0.00 |
| Sichuan | Butuo | 1,594.39 | 0.00 | 0.00 | 0.00 |
| Sichuan | Cangxi | 0.00 | 365.21 | 1,969.93 | 6.64 |
| Sichuan | Chengdu District | 0.00 | 1,034.40 | 334.22 | 0.74 |
| Sichuan | Chongzhou City | 6.64 | 635.99 | 157.15 | 306.19 |
| Sichuan | Dazhou City | 0.00 | 39.84 | 219.87 | 27.30 |
| Sichuan | DA | 0.00 | 774.69 | 1,573.73 | 496.54 |
| Sichuan | Dayi | 39.10 | 377.75 | 434.57 | 373.33 |
| Sichuan | Dazhu | 0.00 | 1,432.81 | 554.83 | 112.88 |
| Sichuan | Danba | 4,681.36 | 0.00 | 0.00 | 0.00 |
| Sichuan | Danling | 82.63 | 356.36 | 22.13 | 0.00 |
| Sichuan | Daofu | 7,122.75 | 0.00 | 0.00 | 0.00 |
| Sichuan | Daocheng | 7,221.61 | 0.00 | 0.00 | 0.00 |
| Sichuan | Derong | 1,827.54 | 0.00 | 0.00 | 0.00 |
| Sichuan | Dechang | 2,218.57 | 0.00 | 0.00 | 0.00 |
| Sichuan | Dege | 11,216.08 | 0.00 | 0.00 | 0.00 |
| Sichuan | Deyang District | 0.00 | 72.30 | 1,032.19 | 0.00 |
| Sichuan | Dujiangyan City | 14.76 | 35.41 | 514.99 | 635.99 |
| Sichuan | Ebian Yi Autonomous County | 516.46 | 1,842.29 | 0.00 | 0.00 |
| Sichuan | Emeishan City | 605.74 | 526.79 | 0.00 | 0.00 |
| Sichuan | Fushun | 35.39 | 2063.58 | 0.00 | 0.00 |
| Sichuan | Ganluo | 408.74 | 1,722.03 | 0.00 | 0.00 |
| Sichuan | Ganzi | 7,422.29 | 0.00 | 0.00 | 0.00 |
| Sichuan | Gao | 1,119.98 | 159.37 | 0.00 | 0.00 |
| Sichuan | Gong | 1,081.62 | 30.99 | 0.00 | 0.00 |
| Sichuan | Gulin | 2,940.88 | 184.45 | 0.00 | 0.00 |
| Sichuan | Guangan City | 0.00 | 17.71 | 1,142.86 | 419.81 |
| Sichuan | Guanghan City | 0.00 | 491.38 | 78.94 | 0.00 |
| Sichuan | Guangyuan District | 0.00 | 812.32 | 4,076.36 | 133.54 |
| Sichuan | Hanyuan | 866.92 | 1,286.73 | 0.00 | 0.00 |
| Sichuan | Hejiang | 121.74 | 1,829.75 | 417.60 | 0.00 |
| Sichuan | Heishui | 4,162.68 | 0.00 | 0.00 | 0.00 |
| Sichuan | Hongyuan | 8,555.56 | 0.00 | 0.00 | 0.00 |
| Sichuan | Hongya | 922.99 | 995.30 | 0.00 | 0.00 |
| Sichuan | Huaying City | 0.00 | 39.10 | 315.78 | 66.40 |
| Sichuan | Huidong | 3,069.26 | 0.00 | 0.00 | 0.00 |
| Sichuan | Huili | 4,335.33 | 0.00 | 0.00 | 0.00 |
| Sichuan | Jiajiang | 442.68 | 284.79 | 0.00 | 0.00 |
| Sichuan | Qianwei | 835.93 | 529.00 | 0.00 | 0.00 |
| Sichuan | Jianyang City | 0.00 | 1,320.67 | 895.69 | 0.00 |
| Sichuan | Jiange | 0.00 | 132.80 | 2,842.75 | 120.26 |
| Sichuan | Jiangan | 446.37 | 433.09 | 0.00 | 0.00 |
| Sichuan | Jiangyou City | 0.00 | 1,747.85 | 1,008.58 | 0.00 |
| Sichuan | Jinchuan | 5495.89 | 0.00 | 0.00 | 0.00 |
| Sichuan | Jintang | 0.00 | 325.37 | 821.17 | 11.07 |
| Sichuan | Jinyang | 1,542.75 | 3.69 | 0.00 | 0.00 |
| Sichuan | Jingyan | 86.32 | 750.35 | 0.00 | 0.00 |
| Sichuan | Jiulong | 6,612.19 | 0.00 | 0.00 | 0.00 |
| Sichuan | Jiuzhaigou | 5,346.86 | 86.32 | 1.48 | 0.00 |
| Sichuan | Junlian | 1,040.30 | 191.09 | 0.00 | 0.00 |
| Sichuan | Kaijiang | 0.00 | 757.72 | 289.96 | 0.00 |
| Sichuan | Kangding | 11,314.94 | 636.72 | 1,263.12 | 10.33 |
| Sichuan | Langzhong City | 0.00 | 848.44 | 1,683.11 | 13.76 |
| Sichuan | Leshan District | 1,342.80 | 1,218.85 | 0.00 | 0.00 |
| Sichuan | Lezhi | 0.00 | 8.12 | 1,412.15 | 0.00 |
| Sichuan | Leibo | 2,131.51 | 527.53 | 0.00 | 0.00 |
| Sichuan | Litang | 14,047.76 | 0.00 | 0.00 | 0.00 |
| Sichuan | Li | 4,250.48 | 101.08 | 15.49 | 0.00 |
| Sichuan | Linshui | 0.00 | 644.10 | 956.93 | 309.14 |
| Sichuan | Longchang | 0.00 | 784.28 | 0.00 | 0.00 |
| Sichuan | Lushan | 204.37 | 261.18 | 576.96 | 196.26 |
| Sichuan | Luhuo | 4,936.64 | 0.00 | 0.00 | 0.00 |
| Sichuan | Luding | 1,744.90 | 397.68 | 0.00 | 0.00 |
| Sichuan | Lu | 16.97 | 2,146.27 | 14.02 | 0.00 |
| Sichuan | Luzhou District | 14.76 | 146.08 | 0.00 | 0.00 |
| Sichuan | Mabian Yi Autonomous County | 767.31 | 1,558.24 | 0.00 | 0.00 |
| Sichuan | Maerkang | 6711.79 | 0.00 | 0.00 | 0.00 |
| Sichuan | Mao | 1,379.69 | 1,610.62 | 897.17 | 6.64 |
| Sichuan | Meishan | 0.74 | 1,336.90 | 3.69 | 0.00 |
| Sichuan | Meigu | 1,502.17 | 948.81 | 0.00 | 0.00 |
| Sichuan | Mianyang District | 0.00 | 905.28 | 661.07 | 0.00 |
| Sichuan | Mianzhu City | 3.69 | 114.36 | 523.10 | 605.00 |
| Sichuan | Mianning | 4,286.63 | 0.00 | 0.00 | 0.00 |
| Sichuan | Mingshan | 49.43 | 551.14 | 10.33 | 0.00 |
| Sichuan | Muli Tibetan Autonomous County | 12,941.80 | 0.00 | 0.00 | 0.00 |
| Sichuan | Muchuan | 734.11 | 658.86 | 2.21 | 0.00 |
| Sichuan | Naxi | 320.21 | 920.78 | 25.82 | 0.00 |
| Sichuan | Nanbu | 194.04 | 1,434.29 | 689.11 | 0.74 |
| Sichuan | Nanchong City | 0.00 | 0.00 | 107.72 | 2.95 |
| Sichuan | Nanjiang | 0.00 | 1,164.25 | 2,236.28 | 64.93 |
| Sichuan | Nanxi | 104.03 | 571.06 | 0.00 | 0.00 |
| Sichuan | Neijiang District | 0.00 | 1,535.37 | 1.48 | 0.00 |
| Sichuan | Ningnan | 1,631.28 | 0.00 | 0.00 | 0.00 |
| Sichuan | Panzhihua City | 14.76 | 0.00 | 0.00 | 0.00 |
| Sichuan | Panzhihua District | 1,924.93 | 16.97 | 0.00 | 0.00 |
| Sichuan | Pengshan | 475.88 | 0.00 | 0.00 | 0.00 |
| Sichuan | Pengzhou City | 22.87 | 121.74 | 998.98 | 303.97 |
| Sichuan | Pengan | 0.00 | 0.00 | 907.50 | 422.02 |
| Sichuan | Pengxi | 0.00 | 0.00 | 1,937.47 | 20.66 |
| Sichuan | Pi | 0.00 | 130.59 | 291.43 | 0.00 |
| Sichuan | Pingchang | 0.00 | 30.99 | 1,706.54 | 521.63 |
| Sichuan | Pingwu | 882.41 | 2,054.78 | 3,114.26 | 22.87 |
| Sichuan | Pingshan | 592.46 | 606.47 | 185.93 | 0.00 |
| Sichuan | Pujiang | 0.00 | 559.99 | 8.12 | 0.00 |
| Sichuan | Puge | 1,858.52 | 0.00 | 0.00 | 0.00 |
| Sichuan | Qingchuan | 1.48 | 394.72 | 2,591.90 | 0.00 |
| Sichuan | Qingshen | 0.00 | 392.51 | 0.00 | 0.00 |
| Sichuan | Qionglai | 0.00 | 957.67 | 392.51 | 19.18 |
| Sichuan | Qu | 0.00 | 190.35 | 1,626.12 | 187.40 |
| Sichuan | Rangtang | 6,310.43 | 0.00 | 0.00 | 0.00 |
| Sichuan | Renshou | 157.89 | 2,350.64 | 74.52 | 0.00 |
| Sichuan | Rong | 0.00 | 1,848.93 | 76.73 | 0.00 |
| Sichuan | Ruoergai | 10,553.53 | 376.28 | 2,293.09 | 0.00 |
| Sichuan | Santai | 0.00 | 501.39 | 3,055.55 | 0.00 |
| Sichuan | Seda | 8,644.10 | 0.00 | 0.00 | 0.00 |
| Sichuan | Shehong | 0.00 | 126.16 | 1,384.12 | 0.00 |
| Sichuan | Shifang | 27.30 | 172.65 | 364.47 | 317.26 |
| Sichuan | Shimian | 2,577.14 | 47.96 | 0.00 | 0.00 |
| Sichuan | Shiqu | 21,514.32 | 0.00 | 0.00 | 0.00 |
| Sichuan | Shuangliu | 0.00 | 1,046.94 | 56.81 | 0.00 |
| Sichuan | Songfan | 7,335.23 | 469.98 | 677.30 | 0.00 |
| Sichuan | Suining District | 0.00 | 0.00 | 1,849.67 | 10.33 |
| Sichuan | Tianquan | 1,014.48 | 1,348.70 | 0.00 | 0.00 |
| Sichuan | Tongjiang | 0.00 | 2,670.11 | 1,449.04 | 48.69 |
| Sichuan | Wanyuan City | 92.96 | 3,266.99 | 743.71 | 0.00 |
| Sichuan | Wangcang | 0.00 | 1,232.13 | 1,797.29 | 7.38 |
| Sichuan | Weiyuan | 0.00 | 1,135.48 | 160.84 | 0.00 |
| Sichuan | Wenjiang | 0.00 | 213.22 | 73.78 | 0.00 |
| Sichuan | Wenchuan | 1,629.07 | 627.87 | 529.00 | 1,334.68 |
| Sichuan | Wusheng | 0.00 | 0.00 | 967.26 | 4.43 |
| Sichuan | Xichang | 2,570.50 | 0.00 | 0.00 | 0.00 |
| Sichuan | Xichong | 73.78 | 804.94 | 266.35 | 0.00 |
| Sichuan | Xide | 2,113.07 | 0.00 | 0.00 | 0.00 |
| Sichuan | Xiangcheng | 4,913.77 | 0.00 | 0.00 | 0.00 |
| Sichuan | Xiaojin | 5,610.99 | 0.00 | 0.00 | 0.00 |
| Sichuan | Xindu | 0.00 | 381.44 | 121.00 | 0.00 |
| Sichuan | Xinjin | 0.00 | 321.68 | 0.00 | 0.00 |
| Sichuan | Xinlong | 8,588.76 | 0.00 | 0.00 | 0.00 |
| Sichuan | Xingwen | 1,333.21 | 50.91 | 0.00 | 0.00 |
| Sichuan | Xuyong | 2,583.05 | 286.27 | 0.00 | 0.00 |
| Sichuan | Xuanhan | 47.96 | 2,934.24 | 1,281.56 | 28.04 |
| Sichuan | Yaan City | 404.32 | 610.90 | 49.43 | 0.00 |
| Sichuan | Yajing | 7,717.42 | 0.00 | 0.00 | 0.00 |
| Sichuan | Yanbian | 3,115.00 | 70.09 | 0.00 | 0.00 |
| Sichuan | Yanting | 16.97 | 1,193.03 | 447.11 | 0.00 |
| Sichuan | Yanyuan | 8,151.98 | 0.00 | 0.00 | 0.00 |
| Sichuan | Yilong | 0.00 | 0.00 | 1,683.67 | 53.86 |
| Sichuan | Yibin City | 679.52 | 404.32 | 0.00 | 0.00 |
| Sichuan | Yibin | 871.34 | 1,966.98 | 176.33 | 0.00 |
| Sichuan | Yingjing | 282.58 | 1,475.61 | 5.90 | 0.00 |
| Sichuan | yingshan | 0.00 | 1.48 | 1,417.32 | 237.57 |
| Sichuan | Yuechi | 0.00 | 1.48 | 630.82 | 832.24 |
| Sichuan | Yuexi | 1,785.48 | 425.71 | 0.00 | 0.00 |
| Sichuan | Changning | 807.89 | 164.53 | 0.00 | 0.00 |
| Sichuan | Zhaojue | 2,625.84 | 0.00 | 0.00 | 0.00 |
| Sichuan | Zhongjiang | 0.00 | 91.49 | 2,093.15 | 6.64 |
| Sichuan | Ziyang | 0.00 | 922.25 | 666.97 | 0.00 |
| Sichuan | Zizhong | 0.00 | 1,663.74 | 79.68 | 0.00 |
| Sichuan | Zitong | 0.00 | 498.02 | 947.34 | 6.64 |
| Sichuan | Zigong District | 0.00 | 810.85 | 0.00 | 0.00 |
| Taiwan | Taiwan | 36,192.80 | 0.00 | 0.00 | 0.00 |
| Tianjin | Baodi | 0.00 | 1,633.18 | 0.00 | 0.00 |
| Tianjin | Ji | 0.00 | 829.72 | 871.60 | 10.65 |
| Tianjin | Jinghai | 0.00 | 1,566.46 | 0.00 | 0.00 |
| Tianjin | Ninghe | 0.00 | 1,528.85 | 0.00 | 0.00 |
| Tianjin | Tianjin District | 0.00 | 3,837.02 | 0.00 | 0.00 |
| Tianjin | Wuqing | 0.00 | 1,693.51 | 0.00 | 0.00 |
| Xinjiang | Aheqi | 13,933.23 | 0.00 | 0.00 | 0.00 |
| Xinjiang | Asuke City | 18,550.65 | 0.00 | 0.00 | 0.00 |
| Xinjiang | Aketao | 22,936.27 | 0.00 | 0.00 | 0.00 |
| Xinjiang | Aletai City | 13,069.94 | 0.00 | 0.00 | 0.00 |
| Xinjiang | Atushen City | 14,146.59 | 0.00 | 0.00 | 0.00 |
| Xinjiang | Awati | 12,902.02 | 0.00 | 0.00 | 0.00 |
| Xinjiang | Bachu | 22,251.43 | 0.00 | 0.00 | 0.00 |
| Xinjiang | Barkol Kazakh Autonomous County | 40,465.58 | 0.00 | 0.00 | 0.00 |
| Xinjiang | Baicheng | 16,358.50 | 0.00 | 0.00 | 0.00 |
| Xinjiang | Bohu | 3,790.99 | 0.00 | 0.00 | 0.00 |
| Xinjiang | Bole City | 8,177.27 | 0.00 | 0.00 | 0.00 |
| Xinjiang | Buerjin | 11,948.51 | 0.00 | 0.00 | 0.00 |
| Xinjiang | Cele | 29,691.83 | 0.00 | 0.00 | 0.00 |
| Xinjiang | Qabqal Xibe Autonomous County | 4,720.80 | 0.00 | 0.00 | 0.00 |
| Xinjiang | Changji City | 8,613.20 | 0.00 | 0.00 | 0.00 |
| Xinjiang | Emin | 10,564.34 | 0.00 | 0.00 | 0.00 |
| Xinjiang | Fuhai | 37,116.44 | 0.00 | 0.00 | 0.00 |
| Xinjiang | Fukang City | 9,320.43 | 0.00 | 0.00 | 0.00 |
| Xinjiang | Fuyun | 35,333.22 | 0.00 | 0.00 | 0.00 |
| Xinjiang | Jiashi | 7,018.31 | 0.00 | 0.00 | 0.00 |
| Xinjiang | Gongliu | 4,319.77 | 0.00 | 0.00 | 0.00 |
| Xinjiang | Habahe | 9,177.54 | 0.00 | 0.00 | 0.00 |
| Xinjiang | Hami City | 81,762.19 | 0.00 | 0.00 | 0.00 |
| Xinjiang | HeBuxaier Mongolian Autonomous County | 32,127.63 | 0.00 | 0.00 | 0.00 |
| Xinjiang | Hejing | 36,631.78 | 0.00 | 0.00 | 0.00 |
| Xinjiang | Heshuo | 13,353.09 | 0.00 | 0.00 | 0.00 |
| Xinjiang | Hetian | 40,509.70 | 0.00 | 0.00 | 0.00 |
| Xinjiang | Hutubi | 9,475.18 | 0.00 | 0.00 | 0.00 |
| Xinjiang | Huocheng | 5,775.06 | 0.00 | 0.00 | 0.00 |
| Xinjiang | Jimunai | 7,898.07 | 0.00 | 0.00 | 0.00 |
| Xinjiang | Jimusaer | 8,078.50 | 0.00 | 0.00 | 0.00 |
| Xinjiang | Jinghe | 12,393.00 | 0.00 | 0.00 | 0.00 |
| Xinjiang | Keshen City | 77.70 | 0.00 | 0.00 | 0.00 |
| Xinjiang | Keping | 8,906.89 | 0.00 | 0.00 | 0.00 |
| Xinjiang | Kela Mayi City | 8,496.65 | 0.00 | 0.00 | 0.00 |
| Xinjiang | Kuche | 15,145.54 | 0.00 | 0.00 | 0.00 |
| Xinjiang | Kuerle City | 7,546.43 | 0.00 | 0.00 | 0.00 |
| Xinjiang | Kuitun City | 1,162.91 | 0.00 | 0.00 | 0.00 |
| Xinjiang | Luntai City | 14,755.70 | 0.00 | 0.00 | 0.00 |
| Xinjiang | Luopu | 13,282.63 | 0.00 | 0.00 | 0.00 |
| Xinjiang | Manasi | 11,207.70 | 0.00 | 0.00 | 0.00 |
| Xinjiang | Maigaiti | 9,594.37 | 0.00 | 0.00 | 0.00 |
| Xinjiang | Miquan City | 3,457.79 | 0.00 | 0.00 | 0.00 |
| Xinjiang | Mingfeng | 54,901.25 | 0.00 | 0.00 | 0.00 |
| Xinjiang | Moyu | 25,099.45 | 0.00 | 0.00 | 0.00 |
| Xinjiang | Mulei Kazakh Autonomous County | 13,914.80 | 0.00 | 0.00 | 0.00 |
| Xinjiang | Nileke | 10,967.34 | 0.00 | 0.00 | 0.00 |
| Xinjiang | Pishan | 38,539.46 | 0.00 | 0.00 | 0.00 |
| Xinjiang | Qitai | 18,888.46 | 0.00 | 0.00 | 0.00 |
| Xinjiang | Qiemo | 133,751.54 | 0.00 | 0.00 | 0.00 |
| Xinjiang | Qinghe | 16,837.23 | 0.00 | 0.00 | 0.00 |
| Xinjiang | Ruoqiang | 197,201.46 | 0.00 | 0.00 | 0.00 |
| Xinjiang | Shawan | 13,023.19 | 0.00 | 0.00 | 0.00 |
| Xinjiang | Shaya | 31,916.91 | 0.00 | 0.00 | 0.00 |
| Xinjiang | Shache | 8,101.54 | 0.00 | 0.00 | 0.00 |
| Xinjiang | Yan Shan | 40,642.72 | 0.00 | 0.00 | 0.00 |
| Xinjiang | Shihezi City | 526.14 | 0.00 | 0.00 | 0.00 |
| Xinjiang | Shufu | 3,609.25 | 0.00 | 0.00 | 0.00 |
| Xinjiang | Shule | 2,171.08 | 0.00 | 0.00 | 0.00 |
| Xinjiang | Tacheng City | 4,360.60 | 0.00 | 0.00 | 0.00 |
| Xinjiang | Taxkorgan Tajik Autonomous County | 23,019.90 | 0.00 | 0.00 | 0.00 |
| Xinjiang | Tekesi | 8,714.61 | 0.00 | 0.00 | 0.00 |
| Xinjiang | Tulufan City | 16,183.99 | 0.00 | 0.00 | 0.00 |
| Xinjiang | Tuokexun | 14,932.84 | 0.00 | 0.00 | 0.00 |
| Xinjiang | Tuoli | 22,199.40 | 0.00 | 0.00 | 0.00 |
| Xinjiang | Weili | 60,491.93 | 0.00 | 0.00 | 0.00 |
| Xinjiang | Wenquan | 6,423.68 | 0.00 | 0.00 | 0.00 |
| Xinjiang | Wensu | 15,515.61 | 0.00 | 0.00 | 0.00 |
| Xinjiang | Urumqi City | 12,277.76 | 0.00 | 0.00 | 0.00 |
| Xinjiang | Wuqia | 19,616.10 | 0.00 | 0.00 | 0.00 |
| Xinjiang | Wushen | 8,739.63 | 0.00 | 0.00 | 0.00 |
| Xinjiang | Wusu City | 16,091.80 | 0.00 | 0.00 | 0.00 |
| Xinjiang | Xinhe | 5,138.95 | 0.00 | 0.00 | 0.00 |
| Xinjiang | Xinyuan | 7,274.47 | 0.00 | 0.00 | 0.00 |
| Xinjiang | Yanqi Hui Autonomous County | 1,430.27 | 0.00 | 0.00 | 0.00 |
| Xinjiang | Yecheng | 27,509.56 | 0.00 | 0.00 | 0.00 |
| Xinjiang | Yining City | 117.21 | 0.00 | 0.00 | 0.00 |
| Xinjiang | Yining | 5,268.67 | 0.00 | 0.00 | 0.00 |
| Xinjiang | Yiwu | 20,719.75 | 0.00 | 0.00 | 0.00 |
| Xinjiang | Yingjisha | 3,418.28 | 0.00 | 0.00 | 0.00 |
| Xinjiang | Yutian | 37,435.15 | 0.00 | 0.00 | 0.00 |
| Xinjiang | Yuming | 6,574.48 | 0.00 | 0.00 | 0.00 |
| Xinjiang | Yuepuhu | 2,946.14 | 0.00 | 0.00 | 0.00 |
| Xinjiang | Zepu | 898.85 | 0.00 | 0.00 | 0.00 |
| Xinjiang | Zhaosu | 11,087.85 | 0.00 | 0.00 | 0.00 |
| Xizang | Anduo | 26,422.37 | 0.00 | 0.00 | 0.00 |
| Xizang | Angren | 27,719.02 | 0.00 | 0.00 | 0.00 |
| Xizang | Basu | 12,580.02 | 0.00 | 0.00 | 0.00 |
| Xizang | Baqing | 10,609.68 | 0.00 | 0.00 | 0.00 |
| Xizang | Bailang | 2,472.43 | 0.00 | 0.00 | 0.00 |
| Xizang | Bange | 105,194.85 | 0.00 | 0.00 | 0.00 |
| Xizang | Bieu | 11,648.94 | 0.00 | 0.00 | 0.00 |
| Xizang | Bishi | 2,625.38 | 0.00 | 0.00 | 0.00 |
| Xizang | Bianba | 9,036.99 | 0.00 | 0.00 | 0.00 |
| Xizang | Bomi | 16,782.56 | 0.00 | 0.00 | 0.00 |
| Xizang | Chaya | 8,464.02 | 0.00 | 0.00 | 0.00 |
| Xizang | Chayu | 31,286.66 | 76.10 | 0.00 | 0.00 |
| Xizang | Cuomei | 4,283.86 | 0.00 | 0.00 | 0.00 |
| Xizang | Cuoqin | 22,598.83 | 0.00 | 0.00 | 0.00 |
| Xizang | Cuona | 34,199.27 | 411.82 | 0.00 | 0.00 |
| Xizang | Damu | 1,366.78 | 0.00 | 0.00 | 0.00 |
| Xizang | Dangxiong | 10,171.00 | 0.00 | 0.00 | 0.00 |
| Xizang | Dingqing | 11,443.78 | 0.00 | 0.00 | 0.00 |
| Xizang | Dingjie | 5,668.54 | 0.00 | 0.00 | 0.00 |
| Xizang | Dingri | 13,813.25 | 0.00 | 0.00 | 0.00 |
| Xizang | Duilongdeqing | 2,695.51 | 0.00 | 0.00 | 0.00 |
| Xizang | Geer | 18,946.13 | 0.00 | 0.00 | 0.00 |
| Xizang | Gaize | 100,338.76 | 0.00 | 0.00 | 0.00 |
| Xizang | Gangba | 3,980.22 | 0.00 | 0.00 | 0.00 |
| Xizang | Geji | 50,826.66 | 0.00 | 0.00 | 0.00 |
| Xizang | Gongbujiangda | 13,000.05 | 0.00 | 0.00 | 0.00 |
| Xizang | Gongga | 2,271.74 | 0.00 | 0.00 | 0.00 |
| Xizang | Gongjue | 6,316.12 | 0.00 | 0.00 | 0.00 |
| Xizang | Jilong | 9,160.84 | 0.00 | 0.00 | 0.00 |
| Xizang | Jiacha | 4,383.09 | 0.00 | 0.00 | 0.00 |
| Xizang | Jiali | 13,398.45 | 0.00 | 0.00 | 0.00 |
| Xizang | Jiangda | 5,241.80 | 0.00 | 0.00 | 0.00 |
| Xizang | Jiangzi | 3,714.62 | 0.00 | 0.00 | 0.00 |
| Xizang | Kangma | 5,781.20 | 0.00 | 0.00 | 0.00 |
| Xizang | Lasa District | 523.73 | 0.00 | 0.00 | 0.00 |
| Xizang | Lazi | 4,378.61 | 0.00 | 0.00 | 0.00 |
| Xizang | Lang | 4,188.37 | 0.00 | 0.00 | 0.00 |
| Xizang | Langkazi | 8,243.93 | 0.00 | 0.00 | 0.00 |
| Xizang | Leiwuqi | 6,217.64 | 0.00 | 0.00 | 0.00 |
| Xizang | Linzhi | 8,754.98 | 0.00 | 0.00 | 0.00 |
| Xizang | Linzhou | 4,562.89 | 0.00 | 0.00 | 0.00 |
| Xizang | Longger | 21,584.19 | 0.00 | 0.00 | 0.00 |
| Xizang | Longzi | 9,921.07 | 0.00 | 0.00 | 0.00 |
| Xizang | Luolong | 8,165.60 | 0.00 | 0.00 | 0.00 |
| Xizang | Luozha | 4,708.37 | 0.00 | 0.00 | 0.00 |
| Xizang | Mangkang | 7,544.88 | 0.00 | 0.00 | 0.00 |
| Xizang | Milin | 9,413.01 | 0.00 | 0.00 | 0.00 |
| Xizang | Motuo | 30,647.29 | 72.37 | 0.00 | 0.00 |
| Xizang | Mozhugongka | 5,539.48 | 0.00 | 0.00 | 0.00 |
| Xizang | Naqu | 16,371.48 | 0.00 | 0.00 | 0.00 |
| Xizang | Nadong | 2,207.58 | 0.00 | 0.00 | 0.00 |
| Xizang | Nanmulin | 8,151.42 | 0.00 | 0.00 | 0.00 |
| Xizang | Nima | 184,113.55 | 0.00 | 0.00 | 0.00 |
| Xizang | Nimu | 3,257.29 | 0.00 | 0.00 | 0.00 |
| Xizang | Nielamu | 7,619.48 | 0.00 | 0.00 | 0.00 |
| Xizang | Nierong | 9820.35 | 0.00 | 0.00 | 0.00 |
| Xizang | Pulan | 12,162.23 | 0.00 | 0.00 | 0.00 |
| Xizang | Qiongjie | 1,037.02 | 0.00 | 0.00 | 0.00 |
| Xizang | Qushui | 1,638.34 | 0.00 | 0.00 | 0.00 |
| Xizang | Qusong | 1,937.51 | 0.00 | 0.00 | 0.00 |
| Xizang | Renbu | 2,108.36 | 0.00 | 0.00 | 0.00 |
| Xizang | Rikeze City | 3,684.78 | 0.00 | 0.00 | 0.00 |
| Xizang | Ritu | 76,188.13 | 0.00 | 0.00 | 0.00 |
| Xizang | Saga | 12,386.05 | 0.00 | 0.00 | 0.00 |
| Xizang | Sajia | 6,040.08 | 0.00 | 0.00 | 0.00 |
| Xizang | Sangri | 2,766.38 | 0.00 | 0.00 | 0.00 |
| Xizang | Shenzha | 23,899.20 | 0.00 | 0.00 | 0.00 |
| Xizang | Shengda | 8,211.11 | 0.00 | 0.00 | 0.00 |
| Xizang | Suo | 6,097.53 | 0.00 | 0.00 | 0.00 |
| Xizang | Suibei | 10,868.56 | 0.00 | 0.00 | 0.00 |
| Xizang | Xietongmen | 14,104.96 | 0.00 | 0.00 | 0.00 |
| Xizang | Yadong | 4,219.70 | 0.00 | 0.00 | 0.00 |
| Xizang | Yanjing | 4,112.27 | 0.00 | 0.00 | 0.00 |
| Xizang | Zharang | 2,164.31 | 0.00 | 0.00 | 0.00 |
| Xizang | Zhada | 25,433.10 | 0.00 | 0.00 | 0.00 |
| Xizang | Zhongba | 25,140.64 | 0.00 | 0.00 | 0.00 |
| Xizang | Zuogong | 9,222.76 | 0.00 | 0.00 | 0.00 |
| Yunnan | Anning City | 1,298.36 | 3.17 | 0.00 | 0.00 |
| Yunnan | Baoshan City | 4,949.46 | 0.00 | 0.00 | 0.00 |
| Yunnan | Binchuan | 1,490.86 | 1,134.38 | 0.00 | 0.00 |
| Yunnan | Cangyuan Wa Autonomous County | 2,507.21 | 0.00 | 0.00 | 0.00 |
| Yunnan | Changning | 3,859.44 | 0.00 | 0.00 | 0.00 |
| Yunnan | Chenggong | 518.87 | 0.00 | 0.00 | 0.00 |
| Yunnan | Chengjiang | 731.17 | 0.00 | 0.00 | 0.00 |
| Yunnan | Chuxiong City | 4,385.44 | 0.00 | 0.00 | 0.00 |
| Yunnan | Daguan | 308.95 | 1,490.86 | 0.00 | 0.00 |
| Yunnan | Dali City | 1,300.74 | 162.39 | 0.00 | 0.00 |
| Yunnan | Dayao | 2,840.71 | 1,363.32 | 0.00 | 0.00 |
| Yunnan | Deqin | 7,615.89 | 0.00 | 0.00 | 0.00 |
| Yunnan | Eshan Yi Autonomous County | 1,949.52 | 0.00 | 0.00 | 0.00 |
| Yunnan | Eryuan | 2,909.63 | 52.28 | 0.00 | 0.00 |
| Yunnan | Fengqing | 3,403.15 | 0.00 | 0.00 | 0.00 |
| Yunnan | Fugong | 2,841.50 | 0.00 | 0.00 | 0.00 |
| Yunnan | Fuming | 907.03 | 147.34 | 0.00 | 0.00 |
| Yunnan | Funing | 5,343.17 | 0.00 | 0.00 | 0.00 |
| Yunnan | Fuyuan | 3,311.26 | 0.00 | 0.00 | 0.00 |
| Yunnan | Gejiu City | 1,570.07 | 0.00 | 0.00 | 0.00 |
| Yunnan | Gengma Dai and Wa Autonomous County | 3,721.60 | 0.00 | 0.00 | 0.00 |
| Yunnan | Gongshan Dulong Nu Nu Autonomous County | 4,613.58 | 0.00 | 0.00 | 0.00 |
| Yunnan | Guangnan | 7,912.16 | 0.00 | 0.00 | 0.00 |
| Yunnan | Estuary Yao Autonomous County | 1,311.83 | 0.00 | 0.00 | 0.00 |
| Yunnan | Heqing | 2,389.18 | 27.73 | 0.00 | 0.00 |
| Yunnan | Honghe | 2,032.70 | 0.00 | 0.00 | 0.00 |
| Yunnan | Huaning | 1,276.18 | 0.00 | 0.00 | 0.00 |
| Yunnan | Huaping | 2,080.23 | 128.33 | 0.00 | 0.00 |
| Yunnan | Huize | 5,639.44 | 406.38 | 0.00 | 0.00 |
| Yunnan | Jianshui | 3,860.23 | 0.00 | 0.00 | 0.00 |
| Yunnan | Jianchuan | 2,345.61 | 0.00 | 0.00 | 0.00 |
| Yunnan | Jiangcheng Hani and Yi Autonomous County | 3,467.31 | 0.00 | 0.00 | 0.00 |
| Yunnan | Jiangchuan | 834.94 | 0.00 | 0.00 | 0.00 |
| Yunnan | Jinping Miao and Yao Nationality Dai Autonomous County | 3,589.31 | 0.00 | 0.00 | 0.00 |
| Yunnan | Jinning | 1,366.49 | 0.00 | 0.00 | 0.00 |
| Yunnan | Jingdong Yi Autonomous County | 4,493.17 | 0.00 | 0.00 | 0.00 |
| Yunnan | Jinggu Dai and Yi Autonomous County | 7,569.15 | 0.00 | 0.00 | 0.00 |
| Yunnan | Jinghong City | 6,985.33 | 0.00 | 0.00 | 0.00 |
| Yunnan | Kaiyuan City | 1,964.57 | 0.00 | 0.00 | 0.00 |
| Yunnan | Kunming District | 2,113.50 | 91.10 | 0.00 | 0.00 |
| Yunnan | Lanping Bai Pumi Autonomous County | 4,557.34 | 0.00 | 0.00 | 0.00 |
| Yunnan | Lancang Lahu Autonomous County | 8,603.72 | 0.00 | 0.00 | 0.00 |
| Yunnan | Lijiang Naxi Autonomous County | 7,785.42 | 0.00 | 0.00 | 0.00 |
| Yunnan | Lianghe | 1,150.23 | 0.00 | 0.00 | 0.00 |
| Yunnan | Lincang | 2,626.03 | 0.00 | 0.00 | 0.00 |
| Yunnan | Longling | 2,832.79 | 0.00 | 0.00 | 0.00 |
| Yunnan | Longchuan | 1,869.51 | 0.00 | 0.00 | 0.00 |
| Yunnan | Lushui | 3,131.44 | 0.00 | 0.00 | 0.00 |
| Yunnan | Luxi | 1,676.22 | 0.00 | 0.00 | 0.00 |
| Yunnan | Ludian | 1,360.94 | 207.55 | 0.00 | 0.00 |
| Yunnan | Luliang | 2,050.92 | 0.00 | 0.00 | 0.00 |
| Yunnan | Lufeng | 3,515.64 | 71.30 | 0.00 | 0.00 |
| Yunnan | Luquan Yi and Miao Autonomous County | 4,307.80 | 10.30 | 0.00 | 0.00 |
| Yunnan | Luxi | 2,930.22 | 0.00 | 0.00 | 0.00 |
| Yunnan | Luoping | 3,084.70 | 32.48 | 0.00 | 0.00 |
| Yunnan | Lvchun | 3,119.55 | 0.00 | 0.00 | 0.00 |
| Yunnan | Malipo | 2,346.40 | 0.00 | 0.00 | 0.00 |
| Yunnan | Maguan | 2,680.69 | 0.00 | 0.00 | 0.00 |
| Yunnan | Malong | 1,700.78 | 0.00 | 0.00 | 0.00 |
| Yunnan | Mengzi | 2,143.60 | 0.00 | 0.00 | 0.00 |
| Yunnan | Menghai | 5,340.79 | 0.00 | 0.00 | 0.00 |
| Yunnan | Mengla | 6,790.45 | 0.00 | 0.00 | 0.00 |
| Yunnan | Mongolian Dai Lahu and Wa Nationality Autonomous County | 1,919.42 | 0.00 | 0.00 | 0.00 |
| Yunnan | Midu | 1,536.80 | 1.58 | 0.00 | 0.00 |
| Yunnan | Mile | 3,956.87 | 0.79 | 0.00 | 0.00 |
| Yunnan | Mojiang Hani Autonomous County | 5,297.22 | 0.00 | 0.00 | 0.00 |
| Yunnan | Muding | 1,517.79 | 0.00 | 0.00 | 0.00 |
| Yunnan | Nanhua | 2,347.19 | 0.00 | 0.00 | 0.00 |
| Yunnan | Nanjian Yi Autonomous County | 1,779.21 | 0.00 | 0.00 | 0.00 |
| Yunnan | Ninglang Yi Autonomous County | 6,170.98 | 0.00 | 0.00 | 0.00 |
| Yunnan | Pingbian Miao Autonomous County | 1,880.60 | 0.00 | 0.00 | 0.00 |
| Yunnan | Pu'er Hani and Yi Autonomous County | 3,704.96 | 0.00 | 0.00 | 0.00 |
| Yunnan | Qiaojia | 3,309.67 | 0.00 | 0.00 | 0.00 |
| Yunnan | Qiubei | 5,095.22 | 0.00 | 0.00 | 0.00 |
| Yunnan | Qujing | 4,366.42 | 0.00 | 0.00 | 0.00 |
| Yunnan | Qujing District | 1,954.28 | 0.00 | 0.00 | 0.00 |
| Yunnan | Ruii | 878.51 | 0.00 | 0.00 | 0.00 |
| Yunnan | Shizong | 2,774.17 | 1.58 | 0.00 | 0.00 |
| Yunnan | Shidian | 1,999.43 | 0.00 | 0.00 | 0.00 |
| Yunnan | Shilin Yi Autonomous County | 1,754.65 | 0.00 | 0.00 | 0.00 |
| Yunnan | Shiping | 2,969.83 | 0.00 | 0.00 | 0.00 |
| Yunnan | Shuangbai | 3,967.96 | 0.00 | 0.00 | 0.00 |
| Yunnan | Shuangjiang Lahu Wa Wa Bulang Dai Autonomous Region | 2,176.08 | 0.00 | 0.00 | 0.00 |
| Yunnan | Shuifu | 54.66 | 381.03 | 15.05 | 0.00 |
| Yunnan | Simao | 3,871.32 | 0.00 | 0.00 | 0.00 |
| Yunnan | Songming | 1,419.56 | 0.00 | 0.00 | 0.00 |
| Yunnan | Suijiang | 262.21 | 514.12 | 41.98 | 0.00 |
| Yunnan | Tengchong | 5,827.18 | 0.00 | 0.00 | 0.00 |
| Yunnan | Tonghai | 726.42 | 0.00 | 0.00 | 0.00 |
| Yunnan | Wanding City | 99.02 | 0.00 | 0.00 | 0.00 |
| Yunnan | Weixin | 1,478.18 | 0.00 | 0.00 | 0.00 |
| Yunnan | Weishan Yi Hui Autonomous County | 2,225.20 | 0.00 | 0.00 | 0.00 |
| Yunnan | Weixi Lisu Autonomous County | 4,687.25 | 0.00 | 0.00 | 0.00 |
| Yunnan | Wenshan | 3,001.52 | 0.00 | 0.00 | 0.00 |
| Yunnan | Wuding | 2,832.00 | 228.94 | 0.00 | 0.00 |
| Yunnan | Xichou | 1,520.17 | 0.00 | 0.00 | 0.00 |
| Yunnan | Xilin | 2,974.59 | 0.00 | 0.00 | 0.00 |
| Yunnan | Ximeng Wa Autonomous County | 1,343.51 | 0.00 | 0.00 | 0.00 |
| Yunnan | Xiangyun | 2,374.12 | 118.03 | 0.00 | 0.00 |
| Yunnan | Xinping Yi Nationality Dai Autonomous County | 4,288.79 | 0.00 | 0.00 | 0.00 |
| Yunnan | Xuanwei City | 5,605.37 | 625.81 | 0.00 | 0.00 |
| Yunnan | Xundian Hui and Yi Autonomous County | 3,649.51 | 0.00 | 0.00 | 0.00 |
| Yunnan | Yanjin | 671.76 | 1,437.78 | 0.00 | 0.00 |
| Yunnan | Yanshan | 3,865.77 | 0.00 | 0.00 | 0.00 |
| Yunnan | Yangbi Yi Autonomous County | 1,911.50 | 8.71 | 0.00 | 0.00 |
| Yunnan | Yaoan | 1,763.36 | 0.00 | 0.00 | 0.00 |
| Yunnan | Yiliang | 1,928.13 | 0.00 | 0.00 | 0.00 |
| Yunnan | Yiiliang | 282.80 | 2,637.12 | 0.00 | 0.00 |
| Yunnan | Yimen | 1,580.37 | 0.00 | 0.00 | 0.00 |
| Yunnan | Yingjiang | 4,413.95 | 0.00 | 0.00 | 0.00 |
| Yunnan | Yongde | 3,260.56 | 0.00 | 0.00 | 0.00 |
| Yunnan | Yongping | 2,836.75 | 0.00 | 0.00 | 0.00 |
| Yunnan | Yongren | 1,935.26 | 265.38 | 0.00 | 0.00 |
| Yunnan | Yongshan | 1,871.10 | 1,032.99 | 0.00 | 0.00 |
| Yunnan | Yongsheng | 4,263.44 | 873.76 | 0.00 | 0.00 |
| Yunnan | Yuxi City | 977.53 | 0.00 | 0.00 | 0.00 |
| Yunnan | Yuanjiang Hani, Yi and Dai Autonomous County | 2,840.71 | 0.00 | 0.00 | 0.00 |
| Yunnan | Yuanmei | 2,037.45 | 43.57 | 0.00 | 0.00 |
| Yunnan | Yuanyang | 2,254.51 | 0.00 | 0.00 | 0.00 |
| Yunnan | Yunlong | 4,540.70 | 0.00 | 0.00 | 0.00 |
| Yunnan | Yun | 3,678.82 | 0.00 | 0.00 | 0.00 |
| Yunnan | Zhaotong City | 687.60 | 1,565.32 | 0.00 | 0.00 |
| Yunnan | Zhengkang | 2569.00 | 0.00 | 0.00 | 0.00 |
| Yunnan | Zhengxiong | 1,276.97 | 2,587.22 | 0.00 | 0.00 |
| Yunnan | Zhenyuan Yi Hani Lahu Autonomous County | 4,118.48 | 0.00 | 0.00 | 0.00 |
| Yunnan | Zhongdian | 11,911.81 | 0.00 | 0.00 | 0.00 |
| Zhejiang | Anji | 1,013.30 | 0.00 | 0.00 | 0.00 |
| Zhejiang | Cangnan | 1,240.02 | 0.00 | 0.00 | 0.00 |
| Zhejiang | Changshan | 4,562.45 | 625.24 | 0.00 | 0.00 |
| Zhejiang | Chunan | 620.01 | 304.34 | 6.98 | 0.00 |
| Zhejiang | Cixi | 0.00 | 673.21 | 200.57 | 224.98 |
| Zhejiang | Deqing | 0.00 | 1,885.32 | 25.29 | 0.00 |
| Zhejiang | Dongyang City | 2.00 |  |  |  |
| Zhejiang | Dongtou | 628.73 | 771.74 | 17.44 | 0.00 |
| Zhejiang | Fenghua City | 114.24 | 1,178.11 | 729.01 | 95.92 |
| Zhejiang | Fuyang City | 30.52 | 762.15 | 0.00 | 0.00 |
| Zhejiang | Haining City | 24.42 | 554.61 | 0.87 | 0.00 |
| Zhejiang | Haiyan | 0.00 | 387.18 | 116.85 | 15.70 |
| Zhejiang | Hangzhou District | 0.00 | 1,540.00 | 168.30 | 131.68 |
| Zhejiang | Huzhou District | 0.00 | 608.68 | 0.00 | 0.00 |
| Zhejiang | Jiashan | 0.00 | 1,182.47 | 0.00 | 0.00 |
| Zhejiang | Jiaxing District | 2,131.23 | 524.96 | 0.00 | 0.00 |
| Zhejiang | Jiande City | 2,308.26 | 0.00 | 0.00 | 0.00 |
| Zhejiang | Jiangshan City | 267.71 | 112.49 | 0.87 | 0.00 |
| Zhejiang | Jinhua District | 1,287.11 | 709.83 | 0.00 | 0.00 |
| Zhejiang | Jinhua | 779.59 | 898.19 | 0.00 | 0.00 |
| Zhejiang | Jinyun | 2,211.46 | 97.67 | 0.00 | 0.00 |
| Zhejiang | Jingning She Autonomous County | 2,562.02 | 48.83 | 0.00 | 0.00 |
| Zhejiang | Kaihua | 911.27 | 646.17 | 2.62 | 0.00 |
| Zhejiang | Lanxi City | 927.84 | 449.97 | 0.00 | 0.00 |
| Zhejiang | Leqing City | 1,363.85 | 389.80 | 0.00 | 0.00 |
| Zhejiang | Lishui City | 315.67 | 2,543.70 | 729.01 | 78.48 |
| Zhejiang | Linan City | 1,363.85 | 861.56 | 0.00 | 0.00 |
| Zhejiang | Linhai City | 3,459.33 | 13.08 | 0.00 | 0.00 |
| Zhejiang | Longquan City | 1,526.00 | 0.00 | 0.00 | 0.00 |
| Zhejiang | Longyou | 733.38 | 227.60 | 0.00 | 0.00 |
| Zhejiang | Ningbo District | 1,656.85 | 204.05 | 0.00 | 0.00 |
| Zhejiang | Ninghai | 688.90 | 59.30 | 0.00 | 0.00 |
| Zhejiang | Ouhai District | 230.22 | 1,294.96 | 0.00 | 0.00 |
| Zhejiang | Panan | 0.00 | 632.22 | 0.00 | 0.00 |
| Zhejiang | Pinghu City | 1,050.79 | 0.00 | 0.00 | 0.00 |
| Zhejiang | Pingyang | 79.35 | 913.88 | 55.81 | 0.00 |
| Zhejiang | Pujiang | 1,771.96 | 1,075.21 | 0.00 | 0.00 |
| Zhejiang | Qingtian | 2,192.28 | 0.00 | 0.00 | 0.00 |
| Zhejiang | Qingyuan | 296.49 | 0.00 | 0.00 | 0.00 |
| Zhejiang | Quzhou District | 1,495.53 | 33.14 | 0.00 | 0.00 |
| Zhejiang | Ruian City | 703.73 | 83.71 | 0.00 | 0.00 |
| Zhejiang | Sanmen | 264.22 | 880.75 | 172.66 | 0.00 |
| Zhejiang | Shangyu City | 0.00 | 117.72 | 2.62 | 0.00 |
| Zhejiang | Shaoxing District | 329.63 | 1,454.54 | 286.02 | 0.00 |
| Zhejiang | Shengzhou City | 1585.35 | 43.60 | 0.00 | 0.00 |
| Zhejiang | Songyang | 0.00 | 0.00 | 0.00 | 0.00 |
| Zhejiang | Suichang | 1,102.24 | 558.10 | 0.00 | 0.00 |
| Zhejiang | Taizhou District | 0.00 | 0.00 | 0.00 | 0.00 |
| Zhejiang | Taishun | 1,431.87 | 189.23 | 0.00 | 0.00 |
| Zhejiang | Tiantai | 1,163.28 | 943.53 | 60.17 | 0.00 |
| Zhejiang | Tonglu | 0.00 | 844.99 | 0.00 | 0.00 |
| Zhejiang | Tongxiang City | 614.78 | 141.27 | 0.00 | 0.00 |
| Zhejiang | Wenling City | 183.13 | 0.00 | 0.00 | 0.00 |
| Zhejiang | Wenzhou District | 1461.52 | 0.00 | 0.00 | 0.00 |
| Zhejiang | Wenchang | 1,678.65 | 109.88 | 0.00 | 0.00 |
| Zhejiang | Wuyi | 1,662.08 | 705.47 | 0.00 | 0.00 |
| Zhejiang | Xianju | 988.88 | 173.53 | 0.00 | 0.00 |
| Zhejiang | Xiangshan | 9.59 | 1,032.48 | 191.85 | 9.59 |
| Zhejiang | XiaoshanCity | 639.20 | 795.29 | 0.00 | 0.00 |
| Zhejiang | Xinchang | 109.00 | 1,154.56 | 12.21 | 0.00 |
| Zhejiang | Yiwu City | 902.55 | 589.49 | 68.02 | 0.00 |
| Zhejiang | Yin | 2,410.28 | 632.22 | 0.00 | 0.00 |
| Zhejiang | Yongjia | 535.42 | 697.62 | 0.00 | 0.00 |
| Zhejiang | Yongkang City | 0.00 | 1,135.38 | 420.32 | 72.38 |
| Zhejiang | Yuhang City | 230.22 | 982.77 | 400.26 | 0.00 |
| Zhejiang | Yuyao City | 351.43 | 14.82 | 0.00 | 0.00 |
| Zhejiang | Yuhuan | 925.22 | 81.97 | 0.00 | 0.00 |
| Zhejiang | Yunhe | 0.00 | 961.85 | 346.19 | 383.69 |
| Zhejiang | Changxing | 6.98 | 4.36 | 0.00 | 0.00 |
| Zhejiang | Zhoushan District | 578.15 | 1,866.14 | 286.02 | 2.62 |
| Zhejiang | Zhuji City | 0.00 | 0.00 | 0.00 | 0.00 |
